# Genome-wide modulation of alternative splicing by a predicted alpha helix in U2AF2

**DOI:** 10.1093/nar/gkaf1347

**Published:** 2025-12-17

**Authors:** Dingwu Xue, Liqiang Ai, Xiaoqin Wang, Yuxin Liu, Yuxuan Zhao, Jingxuan Ma, Ranhui Duan, Long Ma

**Affiliations:** Furong Laboratory, Center for Medical Genetics, School of Life Sciences, Central South University, Changsha, Hunan 410078, China; Furong Laboratory, Center for Medical Genetics, School of Life Sciences, Central South University, Changsha, Hunan 410078, China; Furong Laboratory, Center for Medical Genetics, School of Life Sciences, Central South University, Changsha, Hunan 410078, China; Furong Laboratory, Center for Medical Genetics, School of Life Sciences, Central South University, Changsha, Hunan 410078, China; Furong Laboratory, Center for Medical Genetics, School of Life Sciences, Central South University, Changsha, Hunan 410078, China; Furong Laboratory, Center for Medical Genetics, School of Life Sciences, Central South University, Changsha, Hunan 410078, China; Furong Laboratory, Center for Medical Genetics, School of Life Sciences, Central South University, Changsha, Hunan 410078, China; Furong Laboratory, Center for Medical Genetics, School of Life Sciences, Central South University, Changsha, Hunan 410078, China; Hunan Key Laboratory of Medical Genetics, Hunan Key Laboratory of Animal Models for Human Diseases, Central South University, Changsha, Hunan 410078, China; The Key Laboratory of Precision Molecular Medicine of Hunan Province, MOE Key Lab of Rare Pediatric Diseases, Central South University, Changsha, Hunan 410078, China

## Abstract

Pre-mRNA alternative splicing generates diverse transcript isoforms from the same pre-mRNAs. The binding of 3’ splice site polypyrimidine tracts (PPTs) by U2AF2 is an essential early step in determining the final splice acceptors. However, the mechanism by which U2AF2 distinguishes various PPTs remains to be fully understood. Here, we provide molecular genetic evidence that a conserved α-helix at the *N*-terminus of U2AF2 RNA recognition motif 1 (RRM1) may be a key motif that modulates PPT recognition. *In vivo* amino acid scanning of a conserved residue in the helix can dynamically modulate genome-wide alternative splicing correlated with specific PPT nucleotides in *Caenorhabditis elegans*. Structural modeling of the helix and molecular dynamics simulation of *C. elegans* U2AF2 binding to a 3’ splice site RNA predicted that sidechains of two conserved residues generate flexible twists within the helix, adjusting the orientations of the nucleotide-contacting sidechains to enable an induced-fit binding to PPT nucleotides. Consistent with this prediction, mutagenesis of key PPT nucleotides in transgenic splicing reporters elicited alternative splicing events aligned with the structural models. Together, our findings support a novel structure-function mechanism by which U2AF2 modulates widespread alternative splicing events.

## Graphical Abstract

**Graphical Abstract:**
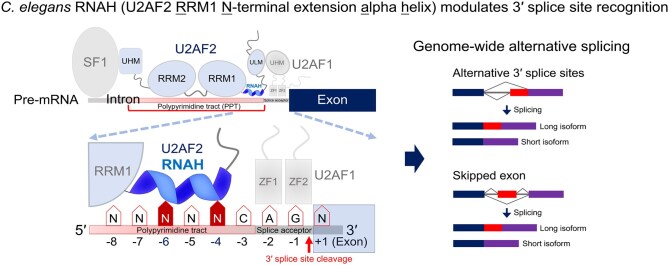

## Introduction

Pre-mRNA splicing (RNA splicing, hereafter) is a fundamental regulatory step for eukaryotic gene expression. RNA splicing is carried out by five small nuclear ribonucleoprotein complexes (U1, U2, U4, U5, and U6 snRNPs) and hundreds of associated splicing factors [1, 2] in a coordinated multi-step catalytic process, resulting in the generation of mature mRNAs through removing non-coding introns and joining coding exons from pre-mRNA transcripts. Using alternative 5’ or 3’ splicing sites (A5SS, A3SS), skipped exons (SE) (also known as cassette exons), intron retention (IR), or mutually exclusive exons (MXE), alternative RNA splicing (alternative splicing, hereafter) can generate multiple mRNA isoforms from the same pre-mRNA transcripts [3, 4]. These mRNA isoforms may have temporary and tissue-specific expression, exhibit variable stabilities, encode proteins with different functions, and play differential roles in complex biological processes. Defective alternative splicing is the cause of numerous diseases [5, 6].

The recognition of 5’ splice sites (5’ss), the branch points (BP) and 3’ splice sites (3’ss) are three key early events of RNA splicing [7]. The recognition of a 5’ss is primarily accomplished by base-pairing between the U1 snRNA and the conserved 5’ss nucleotides “GU” [8–10]. The recognition of a BP is first achieved by the splicing factor SF1 [11–13], followed by base-pairing between the U2 snRNA and the consensus BP nucleotides [2, 14]. Interestingly, the recognition of a 3’ss is largely achieved by protein-RNA interactions between the U2AF heterodimer and the 3’ss nucleotides [15–20] with the involvement of other splicing factors [21, 22].

A 3’ss consists of the 5’ pyrimidine-rich polypyrimidine tract (PPT) and the highly conserved 3’ splice acceptor “AG” [23]. Though uracil (U) forms the consensus sequence of PPTs, cytosine (C), guanine (G) and adenine (A) often appear at different positions with variable frequencies [6, 23]. These variations can affect the dynamic binding of PPTs by U2AF2 [24–26] and potentially the selection of alternative 3’ss [27].

Studies in past decades have significantly enhanced the understanding of how a 3’ss is recognized by the U2AF proteins. The recognition of the conserved 3’ splice acceptor is achieved by the two zinc fingers at the N-terminal and C-terminal extensions of the UHM motif of U2AF1 (also known as U2AF small subunit or U2AF35) [18–20, 28, 29]. The U2AF1 UHM motif interacts with the ULM motif of U2AF2 to form the U2AF heterodimer [16, 17, 30–34]. The PPT upstream of the 3’ splice acceptor is recognized by the two RRMs (RNA recognition motif) in U2AF2 (also known as U2AF large subunit or U2AF65) [16, 22, 35]. U2AF2 adopts flexible conformations that are dynamically affected by interactions between the two RRMs, the interactions between U2AF1 and U2AF2, and the binding of different RNA molecules [25, 36–41]. In addition, the N-terminal and C-terminal extensions of RRM1 and RRM2, respectively, also make contacts with PPT nucleotides [25, 42–44]. U2AF2 can bind most intronic PPTs in the human genome [45, 46] and regulate alternative splicing of a variety of genes [47–49].

U2AF1 mutations are major causes of the myelodysplastic syndrome and some cancers [50–53]. Though less frequently found in cancers compared to U2AF1 mutations, U2AF2 mutations were recently identified to cause neurodevelopmental disorders (NDD) in patients presenting with overlapping cognition impairments, hypotonia, seizures, autistic features, and various behavioral disturbances [54–57]. Specifically, the mutational hotspots are located in a region that includes RRM1 and its N-terminal alpha helix extension of human U2AF2 (hU2AF2, hereafter) [26, 42, 44, 54, 57, 58], or in some cases the RRM2 motif and the spanning region between RRM1 and RRM2 [57]. Though causing overlapping NDD symptoms, these *hU2AF2* mutations differentially affect the splicing of a reporter gene [57].

Studies in *Caenorhabditis elegans* have revealed novel mechanisms underlying RNA splicing [20, 59–69]. Unlike the long PPT of humans, *C. elegans* has a short PPT with a consensus “UUUUC” sequence preceding the 3’ splice acceptor “AG” [63, 69, 70]. Altering the “UUUUC” sequence affects *C. elegans* UAF-1 (U2AF2 ortholog) binding to PPT RNAs and alternative splicing [24, 61].

We previously identified a missense mutation, *n4588*, in *uaf-1* that resulted in the substitution of a conserved threonine at position 180 with isoleucine (T180I). This mutation causes temperature-sensitive (*ts*) lethality, suppresses the locomotion defect of *unc-93(e1500)* mutants, and alters A3SS and/or SE events in two genes [64, 66, 71, 72]. Intragenic mutations at or near *T180I* suppress the *ts*-lethality and alleviate the defective alternative splicing caused by *uaf-1(n4588)* [64], suggesting the functional importance of these residues. UAF-1 T180 is orthologous to hU2AF2 T145 [64], which lies at the center of the N-terminal alpha helix extension of the RRM1 motif, and is among the mutational hotspots for NDD [57]. Previous structural studies found that a few residues of the alpha helix extension form hydrogen bonds (H bonds, hereafter) with PPT nucleotides [26, 42, 43, 44, 58]. These findings together raise important questions as to how this alpha helix of U2AF2 modulates alternative splicing *in vivo* and what structural mechanism might underlie its function. To simplify the presentation in this study, we named the U2AF2 RRM1 N-terminal extension alpha helix RNAH hereafter.

To investigate how UAF-1 RNAH modulates alternative splicing *in vivo* and to understand the structural basis of its function, we examined the effects of multiple *C. elegans uaf-1(T180X)* mutations (X represents a non-threonine amino acid) on genome-wide alternative splicing. These analyses identified specific PPT nucleotides associated with distinct alternative splicing patterns, suggesting that RNAH can dynamically and in some cases bidirectionally modulate alternative splicing events. To explore the underlying structural mechanism, we performed structural modeling and molecular dynamics simulations, which predicted an induced-fit mechanism enabling flexible RNAH binding to a 3’ss PPT RNA. Aligned with the transcriptome findings and structural prediction, the T180I mutation reduced UAF-1 binding to a non-consensus 3’ss RNA, and mutagenesis of specific PPT nucleotides in transgenic reporters produced correlated alternative splicing events.

## Materials and methods

### Strains

*C. elegans* were grown as described [73] at 20°C unless otherwise indicated. Strains used in the study include:

N2 (wild type)

MT16492 *uaf-1(n4588) III* [64]

CSM1019 *uaf-1(mac489) III*

CSM1385 *uaf-1(mac538) III*

CSM1386 *uaf-1(mac539) III*

CSM1387 *uaf-1(mac540) III*

CSM1388 *uaf-1(mac541) /sC1[dpy-1(s2170) macIs1] III*

CSM1389 *uaf-1(mac542) /sC1[dpy-1(s2170) macIs1] III*

CSM1390 *uaf-1(mac543) III*

CSM1391 *uaf-1(mac544) III*

CSM1392 *uaf-1(mac545) III*

CSM1393 *uaf-1(mac546) /sC1[dpy-1(s2170) macIs1] III*

CSM1394 *uaf-1(mac547) /sC1[dpy-1(s2170) macIs1] III*

CSM1395 *uaf-1(mac548) III*

CSM1396 *uaf-1(mac549) /sC1[dpy-1(s2170) macIs1] III*

CSM1397 *uaf-1(mac550) III*

CSM1402 *uaf-1(mac551) III*

CSM1403 *uaf-1(mac552) III*

CSM1404 *uaf-1(mac553) III*

MT19321 *unc-93(e1500) III*

MT15403 *uaf-1(n4588) unc-93(e1500) III*

CSM1265 *uaf-1(mac489) unc-93(e1500) III*

CSM1424 *uaf-1(mac538) unc-93(e1500) III*

CSM1425 *uaf-1(mac539) unc-93(e1500) III*

CSM1426 *uaf-1(mac540) unc-93(e1500) III*

CSM1427 *uaf-1(mac541) unc-93(e1500) /sC1[dpy-1(s2170) macIs1] unc-93(e1500) III*

CSM1429 *uaf-1(mac543) unc-93(e1500) III*

CSM1430 *uaf-1(mac544) unc-93(e1500) III*

CSM1431 *uaf-1(mac545) unc-93(e1500) III*

CSM1432 *uaf-1(mac548) unc-93(e1500) III*

CSM1434 *uaf-1(mac550) unc-93(e1500) III*

CSM1435 *uaf-1(mac551) unc-93(e1500) III*

CSM1441 *uaf-1(mac552) unc-93(e1500) III*

CSM1436 *uaf-1(mac553) unc-93(e1500) III*

CSM600 *rbm-5(n5119) I* [74]

CSM599 *rbm-5(n5132) I* [74]

CSM1467 *rbm-5(n5119) I; uaf-1(mac551) III*

CSM1468 *rbm-5(n5132) I; uaf-1(mac551) III*

CSM1462 *rbm-5(n5119) I; uaf-1(mac552) III*

CSM1463 *rbm-5(n5132) I; uaf-1(mac552) III*

CSM1469 *rbm-5(n5119) I; uaf-1(mac553) III*

CSM1470 *rbm-5(n5132) I; uaf-1(mac553) III*

### Plasmids

To construct the *Pmyo-3::uaf-1a_cDNA(wt)::GFP* plasmid, full-length *uaf-1a* cDNA (1488 bp, NCBI RefSeq: NM_001027796.4) was amplified from *C. elegans* wild-type cDNAs and subcloned into the pPD93.97 vector using *Bam*HI/*Age*I sites to form a *uaf-1a::GFP* fusion coding sequence. Site-directed mutagenesis (Takara In-Fusion® HD Cloning Kit, No. 639650) was performed on *Pmyo-3::uaf-1a_cDNA(wt)::GFP* to generate *Pmyo-3::uaf-1a_cDNA(mut)::GFP* plasmids.

To construct pET-28a(+)_ *His::uaf-1a_cDNA::FLAG* used for purifying UAF-1 protein, amplified full-length *uaf-1a* cDNA was recombined into the pET-28a(+) vector using the Takara In-Fusion® HD Cloning Kit (No. 639650), with a FLAG tag coding sequence introduced before the stop codon. Site-directed mutagenesis was performed on pET-28a(+)*_His::uaf-1a_cDNA::FLAG* to generate pET-28a(+)*_His::uaf-1a(T180I)_cDNA::FLAG*.

To construct the *PCMV::hU2AF2_cDNA(wt)::FLAG* plasmid, *hU2AF2* cDNA (1425 bp, NCBI RefSeq: NM_007279.3) was amplified from HEK293T cell cDNA and subcloned into the pcDNA3.1 vector using *Bam*HI/*Xho*I restriction sites. A FLAG tag coding sequence was included in the reverse PCR primer before the stop codon. Mutagenesis was performed on *PCMV::hU2AF2_cDNA(wt)::FLAG* to generate the *PCMV::hU2AF2_cDNA(mut)::FLAG* plasmid.

To construct *PCMV::hU2AF1_cDNA(wt)::HA* or *PCMV::uaf-2_cDNA(wt)::HA* plasmid, 720 bp of *hU2AF1* cDNA (NCBI RefSeq: NM_001025203.1) and 855 bp of *C. elegans uaf-2* (*U2AF1* ortholog) cDNA (NCBI RefSeq: NM_070635.6) were amplified from HEK293T cDNA or wild-type *C. elegans* cDNA and subcloned into the pcDNA3.1 vector using *BamH*I/*Xho*I restriction sites. The HA tag coding sequence was introduced using the reverse PCR primers before the stop codon.

RNA-Seq of a U2AF2 T145I knockin mouse strain (analysis ongoing) that we previously generated identified an RA3SS event in the *Nfrkb* gene. The annotated transcripts of human *NFRKB* exhibited a conserved splicing pattern (https://www.ncbi.nlm.nih.gov/gene/4798). Hence, we constructed the *hNFRKB* alternative splicing reporter. To construct the *hNFRKB* minigene plasmid, 809 bp (including a downstream portion of intron 5, the whole exon 6, and an upstream portion of intron 6) of *hNFRKB gDNA* (NCBI RefSeq: NC_000011.10: c129885362-129884554) was amplified from human genomic DNAs and subcloned into the pGint vector [75] using *Bam*HI/*Sal*I restriction sites. The full sequence of the *hNFRKB* reporter is provided in the Raw Data.

To construct the *C. elegans* splicing reporter plasmids, we first utilized the previously described *Punc-93::unc-93(E8-I8-E9-I9-E10)* reporter [64]. Mutagenesis was performed on this reporter to generate additional mutant reporters. To construct the *attf-5* reporter plasmid, a 325 bp gDNA fragment (including exon 9, intron 9, and exon 10) of *attf-5* (NCBI RefSeq: NC_003284.9: 791926-792250) was amplified from wild-type gDNA and subcloned into the pPD93.97 vector using *BamH*I/*Age*I restriction sites to generate the *Pmyo-3::attf-5(E9-I9-E10)* plasmid. Mutagenesis was performed on *Pmyo-3::attf-5(E9-I9-E10)* to generate additional mutant reporters. The full sequences of the *unc-93* and *attf-5* reporter plasmids are provided in the Raw Data.

PCR primers are listed in Supplementary Table S5

### Transgenes

Germline transgene experiments were performed as described [76] with minor modifications. For *uaf-1* rescue experiments, the transgene mixtures containing 10 ng/μL of the transgenes of interest with 2.5 ng/μL pCFJ90 *(Pmyo-2::mCherry)* [77] as a co-injection marker were injected into wild-type animals to generate at least two stable lines. The transgenes were then crossed into *uaf-1(n4588) unc-93(e1500) III* animals.

### RNA extraction

Synchronized *C. elegans* were collected at the L4 larval stage. Total RNA was extracted using TRI Reagent™ (Thermo Fisher, Cat. No. AM9738) and treated with RNase-Free DNase I (New England Biolabs, Cat. No. M0303S), followed by incubation at 80°C for 2 min to inactivate DNase I. For RNA extraction from eggs, eggs were collected from adult animals by bleaching and washed three times with ddH_2_O before being subjected to TRI Reagent™ extraction.

### Illumina RNA sequencing

Total RNA integrity and quantity were measured using the Agilent 2100/4200 system (Agilent Technologies, USA). High-quality samples with RNA Integrity Number (RIN) ≥ 8.0 were used for subsequent library preparation. RNA-Seq libraries were constructed using the Hieff NGS™ Ultima Dual-mode mRNA Library Prep Kit for Illumina (Yeasen Biotechnology, Cat. No. 12301ES) following the manufacturer’s instructions. Libraries were quantified using Qubit dsDNA HS Assay (Invitrogen, Cat. No. Q32851). PE150 (paired-end 150 bp) sequencing was performed at Berry Genomics (China) using an Illumina NovaSeq 6000.

### RNA-Seq data analyses

RNA-Seq data quality was assessed using FastQC. Reads were successfully mapped to the *C. elegans* WBcel235 (https://www.ncbi.nlm.nih.gov/datasets/genome/GCF_000002985.6/) using HISAT2 (http://daehwankimlab.github.io/hisat2/). Gene expression was quantified using StringTie (https://ccb.jhu.edu/software/stringtie/), both as Fragments Per Kilobase of transcript per Million mapped reads (FPKM) and as raw count data. The mRNA expression Z-score was calculated based on FPKM, and the heat map was generated in R (4.1.2) using ComplexHeatmap (v. 2.10.0) (bioconductor.org). Differential expression analysis between experimental groups was performed using DESeq2 v1.40.2 [78]. Genes with *P* < 0.05, q < 0.01 and |log2FoldChange| > 1 were considered differentially expressed. Alternative splicing (AS) analysis was conducted using rMATS-turbo v4.1.0 to identify exon skipping, alternative 5' or 3' splice sites, mutually exclusive exons, and intron retention. Novel splice site detection was enabled to capture both annotated and cryptic events. Exon inclusion was quantified using Percent Spliced In (PSI) values (equal to percent cassette exon inclusion). Events with low reads (percentile < 10 in either group) were filtered out. Differential splicing events defined by *P* < 0.05 and |ΔPSI| > 0.1 or by FDR < 0.05 and |ΔPSI| > 0.1 between conditions largely overlapped. A few splicing events with *P* < 0.05 but FDR > 0.05 were validated by RT-PCR, implying that the FDR cutoff was over-stringent. We therefore chose splicing events defined by *P* < 0.05 for analyses in the study.

Using RNA-Seq data, we identified RA3SS and SE events with a more than (>) 10% change between a mutant and wild type. To evaluate the significance of nucleotide frequency, we grouped all 3’ss, regardless of whether they were proximal or distal sites, with increased usage in a mutant. For these 3’ss, we calculated the nucleotide distribution at each position (−3 to −8) and compared it to the corresponding nucleotide distribution in the wild type using Z-test analysis. A similar analysis was also conducted for all 3’ss with decreased usage in mutants.

For RA3SS events, each nucleotide at each position of the two alternative 3’ss was assigned a score corresponding to the percentage use of its respective 3’ss. The average score of the same nucleotide at the same position of all 3’ss was calculated as the weighted frequency. The weighted frequencies of three replicates of a mutant were compared to those of wild type using one-way ANOVA with Dunnett’s multiple comparison test to assess the significance.

### Clustering analysis based on splicing events

To evaluate how similarly UAF-1 mutations influenced genome-wide alternative splicing, a matrix was constructed consisting of splicing event names as rows and mutations as columns, with the values representing the mean inclusion levels (percent 3’ss usage or cassette exon inclusion) for each splicing event across the mutants. The data were standardized using Z-score normalization to ensure comparability across splicing events. The Canberra distance metric was used to calculate pairwise distances between strains. Hierarchical clustering was then performed using the average linkage method to group samples based on their similarity. A dendrogram was generated to visualize the clustering results and to illustrate the relationships among strains. All analyses were conducted using R software (v4.1.2).

### RT-PCR examination of splicing events

First-strand cDNA was synthesized with random hexamer oligonucleotides using Maxima First Strand cDNA Synthesis Kit (Thermo Fisher, Cat. No. K1642).

For AA3SS events (∆3SS ≤ 18), gene-specific primers (10 µM) were used in a PCR reaction with 1–2 µL of first-strand cDNA. The PCR cycling conditions were as follows: annealing at 55–60°C for 30 s, elongation at 72°C for 15 s, 30–32 cycles. Around 15 µL of product was separated on a 20% polyacrylamide gel at a constant voltage of 110 V for 10–11 h. After electrophoresis, the gel was immersed in a 0.5 x TBE solution containing 1:5000 gelstain (TransGen Biotech, Cat. No. GS101) for 60–90 min and visualized using a UV transilluminator with 300 nm excitation.

For RA3SS, SE, RI, and MXE events, PCR conditions like those for AA3SS with 30–50 s elongation were adopted. Around 10–20 µL of PCR product was separated on a 2.5% agarose gel (containing 1:10000 diluted gelstain) at a constant voltage of 110 V for 60–70 min and visualized using a UV transilluminator with 300 nm excitation.

Three biological replicates were analyzed for each strain, and the molar ratios of each splice isoform were quantified using the NIH ImageJ software. Primer sequences are listed in Supplementary Tables S6 and S7.

We evaluated the weighted nucleotide frequencies in RA3SS events validated by RT-PCR as described above.

### CRISPR/Cas9-based homologous recombination

We followed the method by [79] with minor modifications. Plasmids for microinjection were purified using OMEGA’s Midi Plasmid Purification kit (Omega Bio-tek, Cat. No. D6904). The injection DNA mixtures include: 50 ng/µL pPD162 *(Peft-3::Cas9-SV40_NLS)*, 25 ng/µL *PU6::dpy-10 sgRNA* and 500 nM *dpy-10* repair template as co-CRISPR marker [80], 25 ng/µL *PU6::uaf-1 sgRNA1*, 25 ng/µL *PU6::uaf-1 sgRNA2* and 500 nM *uaf-1* repair template with random amino acid-encoding codons at the position of aa180. Dumpy and/or roller F_1_ progeny of injected P_0_ animals were picked to individual plates and allowed to lay eggs. The F_1_ adult was examined by PCR using primers spanning the targeted region of the *uaf-1* locus. PCR fragment sequences were determined by Sanger sequencing to identify mutations in *uaf-1*.

In total, we isolated 852 dumpy and/or roller F_1_ animals. 109 of these animals carried *uaf-1* mutations, among which 81 were deletions. We kept two strains with frameshift mutations (mac546 G194Rfs*197 and mac547 V179Mfs*181) and three strains with in-frame deletions (*mac548 S178delV179L, mac550 V179_T180del* and *mac549 Y187del*). The other 27 strains carried missense mutations encoding 14 different T180X substitutions. However, a *T180Y* mutation was lost, potentially due to heterozygous sterility. A *T180H* mutation carried a closely linked wild-type duplication and was discarded. The other 25 mutations were maintained as homozygotes if their homozygotes were viable or heterozygotes with a balancer if their homozygotes were inviable or sterile. These mutations encode 12 distinct T180X substitutions, including a T180I like that encoded by *uaf-1(n4588)*. Mutants with obvious phenotypes (*ts*-lethality or sterility) were backcrossed three times. Six DAPI-stained bodies were observed in dissected diakinesis stage oocytes of *uaf-1(n4588)(T180I), uaf-1(mac553)(T180I)*, and *uaf-1(mac539)(T180C)* mutants. Homozygous mutants were analyzed in all experiments.

### Temperature-sensitive phenotype analyses

Animals were grown at 15°C and eggs collected by bleaching were synchronized overnight to L1 larva in M9 at 20°C. ∼100 L1 larva were transferred to an NGM agar plate and grown at 15°C, 20°C, 22.5°C, or 25°C for 3–4 days until most animals became adults. Protruding vulva (Pvl) phenotype and sterility (Ste) phenotype (lack of eggs in the gonad) were examined under a dissecting microscope. Some adults were then transferred to new plates and grown at the same temperatures at which they developed. These animals were observed in the following days for egg-laying, hatching, and larval development. Animals that failed to propagate completely were considered inviable (lethality). Animals with unhealthy propagation compared to wild-type, but eventually consumed all the food, were considered partially inviable (partial lethality).

### Locomotion (body bends)

Locomotion was quantified as described [64] with modifications. Synchronized L4 worms were transferred to fresh NGM plates pre-seeded with a thin lawn of bacteria and were allowed to grow into young adults overnight (16–18 h). The tail of each worm was gently touched with a worm pick to trigger locomotion. Body bends were counted for 30 s after the locomotion was initiated.

### Lifespan assay

Synchronized L4 animals were transferred to new plates and allowed to grow for 3 days. Survived adults (*n* = 80, counted as 100%) were transferred to a new plate. Every 2 days, adult animals that responded to prodding by a worm pick were considered alive and transferred to a new plate. During the process, animals with internal hatching, vulval bursting, or those unaccounted for were excluded.

### U2AF2 RNAH motif conformation analysis

All RNAH structures were derived from AlphaFold predicted models (https://alphafoldserver.com) and analyzed using PyMOL 2.5 (open-source version, https://github.com/schrodinger/pymol-open-source). The parameters of the RNAH motif, including the helical tilt angle, local twist, and root mean square deviation (RMSD), as well as the generation of the helical axis centerline, were calculated using custom Python scripts (generated and modified with the assistance of ChatGPT) integrated with PyMOL. The detailed methods are outlined as follows:

Region selection.

To simplify the analysis, the full predicted structures of U2AF2s were truncated to include residues corresponding to UAF-1 (p.176_190) or hU2AF2 (p.138_155), a region encompasses the first β-sheet of the RRM1 motif and the full RNAH α-helix.

Identification of α-helical regions.

The secondary structure assignment tool (dss) in PyMOL was used to identify the α-helical regions. The Cα atoms of the α-helical regions were automatically detected, and their coordinates were extracted for further analysis.

RMSD calculation.

PyMOL’s “align” function was used to align the structures of the truncated wild-type and mutant RNAH-RRM1_β1. The reference alignment structure was RRM1_β1 (UAF-1 (p.185_189) or hU2AF2 (p.150_154). The “rms_cur” function in PyMOL was used to calculate the RMSD of the mutant RNAH structure (UAF-1 (p.176_184) or hU2AF2 (p.141_149)) compared to the wild type.

Helical axis and helical tilt angle.

For each RNAH α-helix, principal component analysis (PCA) was performed on the Cα coordinates to calculate the helical axis. The inclination of the helical axis relative to the reverse extension of the RRM1_β1 principal axis was determined by calculating the dot product of the normalized vectors.

Helix local twist.

Local geometric properties were calculated using overlapping fragments of four consecutive Cα atoms. The twist was determined based on the dihedral angle formed by four sequential atoms. Vector operations for twist calculations were performed using the NumPy library.

Visualization of the helical axis centerline.

To assist in the visualization of α-helical geometry, a centerline was generated for each α-helix using a custom script. The script calculated the geometric center of the Cα atoms and applied cubic spline interpolation to generate a smooth, dashed centerline. The centerline was displayed in PyMOL as a CGO (compiled graphics object).

Data export and analysis.

The calculated parameters, including helical tilt angle, local twist, and RMSD, were exported as tab-delimited text files or Excel spreadsheets for further statistical analysis. Numerical computations were performed using standard Python libraries such as NumPy and SciPy, and data visualizations were generated using Matplotlib.

### Molecular dynamics simulations (MDs)

The molecular dynamics (MD) analysis was carried out using the GROMACS 2022 package (www.gromacs.org). We utilized the AlphaFold online platform (https://alphafoldserver.com/) to simulate bindings of wild-type and mutant UAF-1 proteins with various *C. elegans* 3' consensus splice site sequences. From these simulations, we selected a pair of models that aligned with previously resolved structures for the hU2AF2-RNA complexes [26, 42, 44]. These models served as the starting structures for MDs. The topology of the UAF-1-RNA complex was constructed using the Amber 14SB force field [81], and pdb2gmx (Gromacs) was employed to convert these structures into a GROMACS-compatible 3D coordinate format. The MD simulations were conducted in a cubic box filled with explicit TIP3P water containing 150 mM NaCl, with molecules positioned 10 Å from the box edges. Ion calculations and system neutralization were performed using the Genion tool (Gromacs). Energy minimization was conducted using the steepest descent method for 5000 steps. Subsequently, restrained dynamics were applied to prevent significant conformational changes during water relaxation, preserving protein structure integrity. The temperature was maintained at 300 K using the V-rescale coupling method (Gromacs), and Berendsen pressure coupling (Gromacs) was employed to mitigate velocity effects. The LINCS algorithm (Gromacs) was used to constrain non-water bonds, while the SETTLE algorithm (Gromacs) was applied to water molecules. The equilibration phase involved 100 000 steps over 100 ps of restrained dynamics. Finally, a 300 ns production MD simulation with a 2 fs time step was performed until stable binding between the protein and RNA was observed. A similar protocol was applied to the mutant protein model. H bond analysis was conducted using GROMACS 2022 with the gmx hbond command, defining an H bond as having a hydrogen atom-electronegative atom (e.g. oxygen or nitrogen) distance < 3.5 Å and a donor-hydrogen-acceptor angle > 150 ^o^ to ensure bond stability.

### Protein purification

*Escherichia coli* BL21 cells transformed with pET-28a(+)*_His::uaf-1a_cDNA::FLAG* were cultured at 37°C until the OD600 reached 0.6–0.8. Protein expression was induced with 0.5 mM IPTG (Sigma, Cat. No. I5502) at 16°C for 16 h. Cells were harvested by centrifugation, and the pellet was resuspended in cold PBS supplemented with 1 mg/mL lysozyme (Sigma, Cat. No. L1667) and protease inhibitor cocktail (Bimake, Cat. No. B14001). followed by sonication (16 cycles of 10 s at 50% output). After sonication, the lysate was centrifuged at 12 000 g for 15 min. The collected supernatant was incubated overnight with PureCube Ni-NTA Agarose (Cube Biotech, Cat. No. 31103) pre-equilibrated with PBS to allow binding of His6-tagged proteins. The resin was thoroughly washed with wash buffer (50 mmol/L Tris-HCl, pH 7.9, 400 mmol/L NaCl, 25 mmol/L imidazole) to remove nonspecifically bound proteins. Finally, the His6-tagged proteins were eluted from the PureCube Ni-NTA Agarose using elution buffer (50 mmol/L Tris-HCl, pH 7.9, 400 mmol/L NaCl, 300 mmol/L imidazole).

### RNA electrophoretic mobility-shift assay (EMSA, gel shift)

The RNA EMSA was performed following the manufacturer’s instructions of the RNA EMSA kit (Beyotime, China, Cat. No. GS606). Recombinant UAF-1 protein (∼1 μg) and 5’ biotin-coupled RNA (0.2 μM) were co-incubated at 25°C for 1 h in a 10 μL buffer containing 300 mM NaCl, 300 mM KCl, 15 mM MgCl2, 40 mM Tris-HCl (PH = 7.2), and 1 mM DTT. The protein-RNA complexes were separated by electrophoresis on a 6% non-denaturing polyacrylamide gel at 4°C for 2–3 h and transferred onto a nylon membrane, which was irradiated by UV at a dose of 120 mJ/cm2 for 2 min. Biotinylated RNA was detected as instructed (Beyotime, China, Cat. No. GS606). The biotin-coupled RNA probes and non-labeled RNA probes were purchased from Sangon; sequences are listed in Supplementary Fig. S22.

### Cell culture and transfection

HEK293T cells were cultured in DMEM medium supplemented with 10% fetal bovine serum (Gibco, Cat. No. 12484028) and 1% penicillin/streptomycin (Thermo Fisher Scientific, Cat. No. 15140163). Cells were maintained in a 6-cm petri dish at 37°C with 5% CO2. A mixture of 500 ng *hU2AF2* expression plasmid (see Plasmids) and 500 ng *GFP-hNFRKB* splicing reporter (see Plasmids) or 500 ng *pypY* splicing reporter [47] was transfected using Lipofectamine 3000 Reagent (Invitrogen, Cat. No. L3000015) at 80% cell confluency according to the manufacturer’s instructions. Cells were harvested after 24 h. For immunoprecipitation, 1 μg of plasmid was transfected, and cells were harvested after 24 h.

### Pull-down, immunoprecipitation, and western blot

For the *C. elegans* U2AF complex pull-down assay, HEK293T cells expressing UAF-2::HA (U2AF1 ortholog) were lysed in NP-40 buffer with protease inhibitors (Bimake, Cat. No. B14001), sonicated (6 cycles, 3 seconds each, 30% power), and centrifuged at 4°C for 20 min. The supernatant was incubated overnight at 4°C with purified His::UAF-1::FLAG protein, and 10 µL Protein A/G magnetic beads (Bimake, China, Cat. No. B23201) preincubated with 1 µg of anti-HA or anti-FLAG antibody for 1 h. The beads were washed 5 times with 1 × PBST, and proteins were eluted with 20 µL 1 × SDS sample buffer, denatured at 100°C for 10 min, and analyzed by SDS-PAGE and western blot. 5% of the supernatant was used as an input control.

For hU2AF complex immunoprecipitation, HEK293T cells co-expressing U2AF2::FLAG and U2AF1::HA were lysed in NP-40 buffer, and immunoprecipitation was performed using Protein A/G magnetic beads pre-incubated with anti-HA or anti-FLAG antibody. Subsequent steps were the same as those in the pull-down assay.

Protein samples from pull-down and immunoprecipitation were separated by SDS-PAGE and then transferred to a PVDF membrane (Millipore, Billerica, MA, USA). After blocking with 1xTBST (TBS with 0.1% Tween 20 with 5% nonfat milk) for 1 h at room temperature, the membranes were incubated with the primary antibodies overnight at 4°C, washed three times in 1xTBST, and then incubated with the secondary antibodies for 1 h at room temperature. Primary antibodies were anti-FLAG (Sigma, Cat. No. F1804, 1:5000), anti-HA (Cell Signaling, Danvers, MA, USA, Cat. No. C29F4, 1:2000), anti-β-Actin (Abcam, Cat. No. ab8226, 1:3000). Secondary antibodies were HRP-conjugated goat anti-mouse IgG (Jackson ImmunoResearch, West Grove, PA, USA, Cat. No.115-035-166, 1:10000) or HRP-conjugated goat anti-rabbit IgG (Jackson ImmunoResearch, Cat. No.111-035-144, 1:10000). The protein bands were visualized using an ECL-chemiluminescent kit.

### Statistics

Statistical tests were performed using GraphPad Prism (GraphPad Software). *P*-values were determined by a two-tailed unpaired Student’s t-test for comparisons between two samples, Tukey’s multiple comparison with one-way ANOVA for multiple comparisons, or Dunnett’s multiple comparisons with one-way ANOVA for comparing multiple groups with a single control group. Lifespan was analyzed using the Mantel–Cox log-rank method. 3’ss nucleotide frequency was analyzed using a two-tailed Z-test.

## Results

### *C. elegans* UAF-1 T180I alters the selection of 3’ss correlated with specific PPT sequences

**Figure 1.**
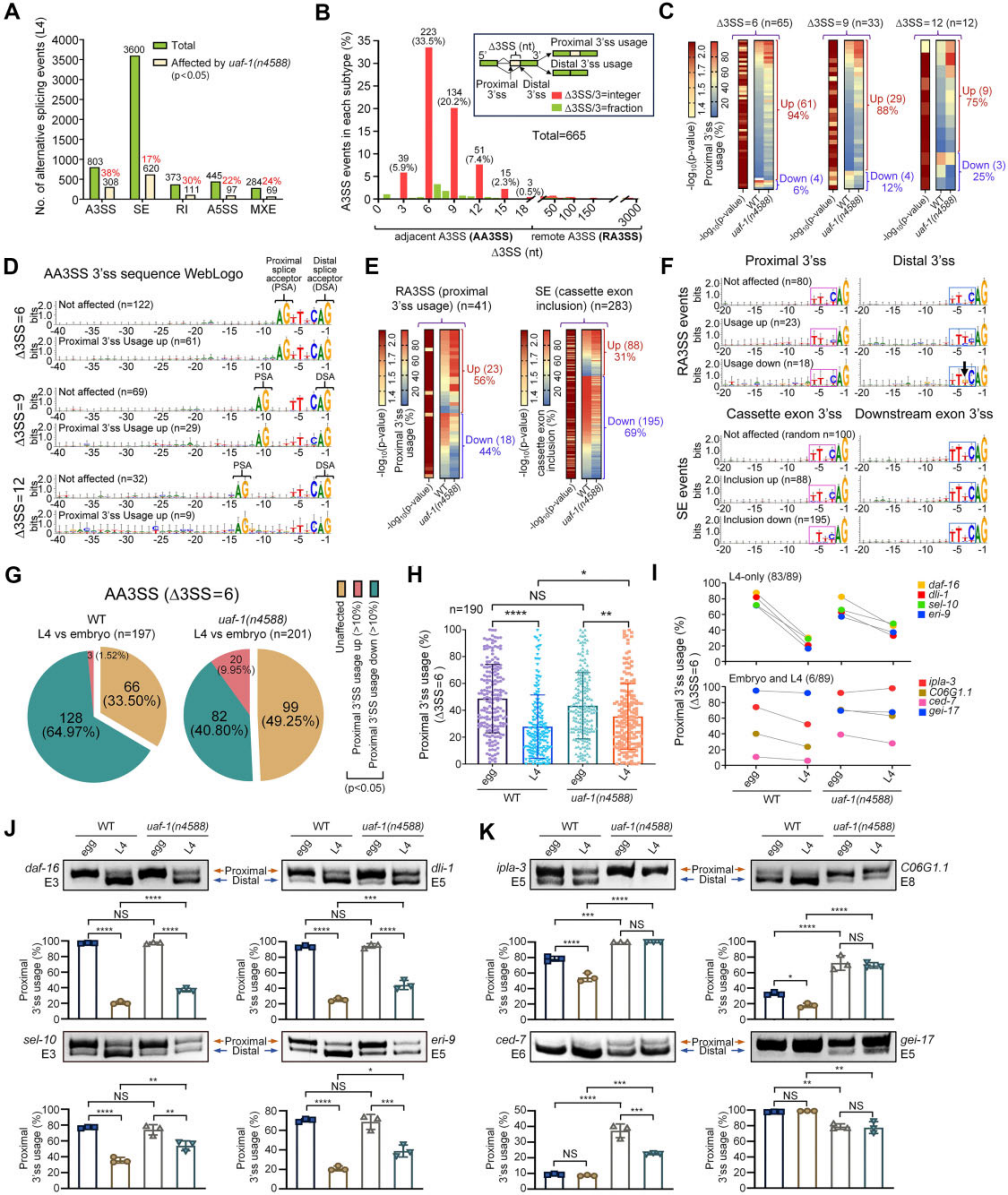
UAF-1 T180I affects genome-wide alternative splicing. (**A**) Alternative splicing events affected by *uaf-1(n4588)(T180I)* at the L4 larval stage. Green bars represent total events shared by wild-type and *uaf-1(n4588)(T180I)* mutants. Yellow bars represent events significantly altered by *uaf-1(n4588)(T180I)*. (**B**) A3SS events were further classified as subgroups based on the nucleotide (nt) lengths (∆3SS) between the proximal (upstream) and distal (downstream) splice acceptors (graph insert). Events with ∆3SS less than or equal to 18 were named adjacent A3SS (AA3SS) events, while those with ∆3SS greater than 18 were named remote A3SS (RA3SS) events. (**C**) Heat maps showing the numbers of different types of AA3SS events exhibiting a more than 10% change in proximal 3’ss usage caused by *uaf-1(n4588)(T180I)*. (**D**) WebLogo comparisons of 3’ss sequences of different AA3SS events. Note: increased (up) proximal 3’ss usage is coupled with decreased (down) usage of the corresponding distal 3’ss in AA3SS and RA3SS events. (**E**) Heat maps showing the number of RA3SS and SE events with a more than 10% change in proximal 3’ss usage and cassette exon inclusion, respectively, caused by *uaf-1(n4588)(T180I)*. (**F**) WebLogo comparisons of proximal and distal 3’ss sequences in RA3SS events, and the preceding 3’ss sequences of the cassette exons (skipping exons) and the downstream exons in SE events. Nucleotides at positions −3 to −6 of 3’ss are outlined, respectively. (**G**) Pie charts showing the number of AA3SS events (∆3SS = 6) with or without an embryo-to-L4 change in proximal 3’ss usage in wild-type or *uaf-1(n4588)(T180I)* animals. (**H**) Scatter plot showing the percent usage of proximal 3’ss in AA3SS events (∆3SS = 6) at the embryonic and L4 larval stages in wild-type and *uaf-1(n4588)(T180I)* animals. Statistics: Tukey’s multiple comparison test with one-way ANOVA. *: *P* < 0.05; **: *P* < 0.01; ****: *P* < 0.0001. NS: not significant. (**I**) *uaf-1(n4588)(T180I)* affected the proximal 3’ss usage in most AA3SS events (∆3SS = 6, 83/89) only at the L4 stage, while in a small number of events (6/89) the effects were at both embryonic and L4 stages. Four representative events of each type are shown in the top (L4-only) and the bottom panel (embryo and L4). (**J**) RT-PCR validation of representative AA3SS events (∆3SS = 6) affected by *uaf-1(n4588)(T180I)* only at the L4 stage. PCR products were separated on a 20% non-denaturing polyacrylamide gel. Quantifications were based on three biological replicates. Statistics: Tukey’s multiple comparison test with one-way ANOVA. *: *P* < 0.05; **: *P* < 0.01; ***: *P* < 0.001; ****: *P* < 0.0001. NS: not significant. (**K**) RT-PCR validation of representative AA3SS events (∆3SS = 6) affected by *uaf-1(n4588)(T180I)* at both embryonic and L4 stages. PCR products were separated on a 20% non-denaturing polyacrylamide gel. Quantifications were based on three biological replicates. Statistics: Tukey’s multiple comparison test with one-way ANOVA. **: *P* < 0.01; ***: *P* < 0.001; ****: *P* < 0.0001. NS: not significant.

To understand how UAF-1 T180 (orthologous to hU2AF2 T145) affects genome-wide alternative splicing, we analyzed the transcriptomes of wild-type and *uaf-1(n4588)* (carrying the original T180I mutation) homozygous mutants [64] at the L4 larval stage. In wild-type animals, SE (skipped exons) were the most common alternative splicing events (*n* = 3600), followed by A3SS (alternative 3’ splice sites, *n* = 803), A5SS (alternative 5’ splice sites, *n* = 445), IR (intron retention, *n* = 373) and MXE (mutually exclusive exons, *n* = 284) (Fig. 1A, green bars). *uaf-1(n4588)(T180I)* significantly affected each type of event (Fig. 1A, yellow bars). Among these, the fraction of affected A3SS events was the highest (308/803, 38%, Fig. 1A), whereas the absolute number of affected SE events was the largest (620/3600, 17%, Fig. 1A).

Focusing on A3SS events with more than (>) 10 junction-spanning reads shared by both wild-type and *uaf-1(n4588)(T180I)* animals (Supplementary Fig. S1A), we found that the nucleotide lengths (Fig. 1B inset, ∆3SS) between the upstream proximal 3’ss and downstream distal 3’ss (Fig. 1B) in most events were less than or equal to (≤) 18 (Fig. 1B). We named these events adjacent alternative 3’ splice sites (AA3SS) events. To distinguish from AA3SS, we named A3SS events with ∆3SS greater than (>) 18 remote alternative 3’ splice site (RA3SS) events.

Consistent with a previous study [68], we found that the ∆3SS of most AA3SS events was equal to 3, 6, 9, 12, 15, or 18 (Fig. 1B, 88.1% of all AA3SS events). Among them, 87.7% consisted of events with ∆3SS equal to 6, 9, or 12 (Fig. 1B). Interestingly, *uaf-1(n4588)(T180I)* primarily caused increased proximal 3’ss usage, i.e. decreased distal 3’ss usage, in these events (Supplementary Fig. S1B, Fig. 1C).

To investigate whether specific 3’ss sequences were associated with the effects of *uaf-1(n4588)(T180I)* on AA3SS events, we performed WebLogo sequence comparisons of the intronic regions up to -40 nt upstream of the distal 3’ss (Fig. 1D). Interestingly, events not affected by *uaf-1(n4588)(T180I)* (Fig. 1D, top subpanels) and those with increased proximal 3’ss usage in *uaf-1(n4588)(T180I)* (Fig. 1D, bottom subpanels) exhibited similar PPT sequences: the proximal 3’ss lacked a consensus, while the distal 3’ss exhibited a TT(T/N)C (N: any nucleotide) consensus from positions −6 to −3 (same order hereafter). The PPTs of AA3SS events with decreased proximal 3’ss usage in *uaf-1(n4588)(T180I)* mutants were not analyzed because of their limited sample sizes (∆3SS = 6 *(n* = 4), 9 (*n* = 4), or 12 (*n* = 3)).

Unlike AA3SS events, RA3SS events affected by *uaf-1(n4588)(T180I)* showed a more balanced distribution, with 56% (n = 23) exhibiting increased and 44% (*n* = 18) exhibiting decreased proximal 3’ss usage (Fig. 1E, left). In their PPTs, a proximal TTNY (Y: pyrimidine) consensus and a distal NTYY consensus were associated with increased proximal 3’ss usage, while a proximal TTNY consensus and a distal TT(G/T)C consensus were associated with decreased proximal 3’ss usage (Fig. 1F, top panel, 2^nd^ and 3^rd^ lines, enclosed). However, for RA3SS events not affected, the consensus for both proximal and distal PPTs was TTNY (Fig. 1F, top panel, 1^st^ line, enclosed). Interestingly, a -4G became obviously associated with increased distal 3’ss usage in *uaf-1(n4588)(T180I)* mutants (Fig. 1F, top panel, arrow). This is consistent with our previous analyses of two other RA3SS events [64, 71], in which a -4G in 3’ss can increase their usage in *uaf-1(n4588)(T180I)* mutants.

3159 of 3600 SE events identified by RNA-Seq had more than 10 junction-spanning reads (Fig. 1A and Supplementary Fig. S1C). Most cassette exons in these events had nt lengths equal to multiples of 3 (Supplementary Fig. S1D). For SE events with more than (>) 10% change caused by *uaf-1(n4588)(T180I)* (Supplementary Fig. S1C, 283 in total), significantly more (*n* = 195, 69%) exhibited decreased cassette exon inclusion compared to those exhibiting increased inclusion (*n* = 88, 31%) (Fig. 1E, right). In SE events not affected and those with cassette exon inclusion increased by *uaf-1(n4588)(T180I)*, the PPTs of the 3’ss preceding the cassette exons (upstream) had a TTNY consensus (Fig. 1F, bottom panel, 1^st^ and 2^nd^ lines, enclosed in red). However, in events in which cassette exon inclusion was decreased by *uaf-1(n4588)(T180I)*, the corresponding consensus was NTTY (Fig. 1F, bottom panel, 3^rd^ line, enclosed in red). In all cases, the downstream PPTs had a TTTY consensus (Fig. 1F, bottom panel, enclosed in blue).

Examining the PPT consensus sequences of the RA3SS and SE events together, it appears that UAF-1 T180I favors the TTNY sequence but disfavors the NTTY sequence. Differently, between affected and unaffected AA3SS events, the PPT consensus sequences lacked a clear difference, implying that mechanisms other than PPT sequences might be involved in the selection of the alternative 3’ss in these events.

### *C. elegans* UAF-1 T180I alters developmentally regulated usage of proximal 3’ss in AA3SS events

Alternative splicing is broadly involved in organ development and tissue function [82, 83]. To investigate whether UAF-1 differentially affects alternative splicing in development, we compared splicing events in the early-stage embryos with those in the L4 larva (1G-K, Supplementary Figs S2 and S3).

Taking AA3SS (∆3SS = 6) events as examples, we found that most events decreased the proximal 3’ss usage (>10%, n = 128, 64.97%, named type 1), while only a few increased the proximal 3’ss usage (>10%, *n* = 3, 1.52%, named type 2) at the L4 stage compared to the embryonic stage in the wild type (Fig. 1G, left). However, *uaf-1(n4588)(T180I)* noticeably reduced the number of type 1 events (*n* = 82, 40.8%) but increased the number of type 2 events (*n* = 20, 9.95%) (Fig. 1G, right). Such a developmental difference appeared to result from the largely comparable proximal 3’ss usage in embryos between *uaf-1(n4588)(T180I)* mutants and wild type (Fig. 1H). Further analysis suggested that *uaf-1(n4588)(T180I)* exerted similar effects on other types of AA3SS events (∆3SS = 9 or 12) (Supplementary Fig. S2A and B).

Of the AA3SS events affected by *uaf-1(n4588)(T180I)*, most were affected only at the L4 stage (Fig. 1I, top panel, and Supplementary Fig. S2C, in parentheses), while a small fraction was affected at both embryonic and L4 stages (Fig. 1I, bottom panel, and Supplementary Fig. S2D, in parentheses). We validated the RNA-Seq results by examining representative events through RT-PCR (Fig. 1J, K, Supplementary Fig. S2E and F). Hence, we propose that UAF-1 T180 might modulate developmental AA3SS events.

Unlike AA3SS events, smaller fractions of RA3SS and SE events exhibited embryo-to-L4 changes in the wild-type (Supplementary Fig. S3A, left). *uaf-1(n4588)(T180I)* slightly decreased the percentages of these events regardless of the direction of the change (Supplementary Fig. S3A, compare right with left). WebLogo sequence comparisons on embryonic RA3SS events showed that a proximal TTNY and a distal NTTY PPT consensus were associated with increased proximal 3’ usage, while a proximal TTNY and a distal TT(G/T)Y consensus were associated with decreased proximal 3’ss usage (Supplementary Fig. S3B, top panel, 2^nd^ and 3^rd^ lines, enclosed). In SE events, increased and decreased cassette exon inclusion were associated with a TTNY and an NTTY consensus, respectively, in the upstream PPTs (Supplementary Fig. S3B, bottom panel, 2^nd^ and 3^rd^ lines, enclosed in red). We further confirmed representative RNA-Seq results through RT-PCR (Supplementary Fig. S3C and D).

It is worth noting that in all types of events, including AA3SS (proximal 3’ss only), RA3SS, and SE, specific nucleotides upstream of position −7 (including −7) in general were not obviously overrepresented in the WebLogo graphs. Weighted nucleotide frequency provided a slightly different scenario (see below).

Together, we hypothesize that specific PPT nucleotides, especially those at positions −4 and −6, were associated with affected RA3SS and SE events in *uaf-1(n4588)(T180I)* mutants at both embryonic and L4 stages (Fig. 1F and Supplementary Fig. S3B).

### *C. elegans* UAF-1 RNAH adopts a flexible alpha helix-like conformation *in silico*

**Figure 2.**
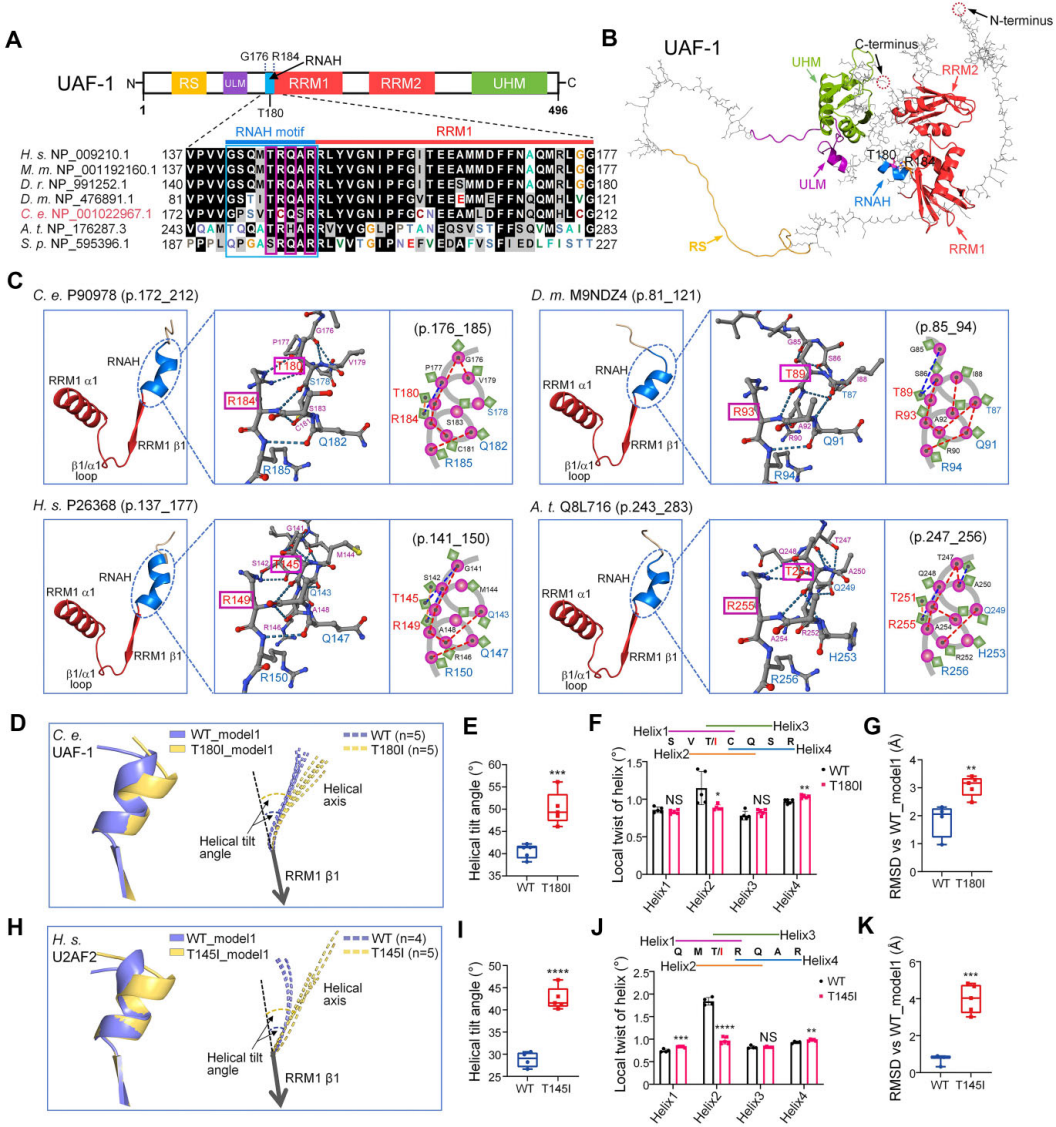
Comparable effects of *C. elegans* UAF-1 T180I and hU2AF2 T145I on AlphaFold-predicted conformations of UAF-1 RNAH and hU2AF2 RNAH, respectively. (**A**) UAF-1 domains and RNAH sequence alignment (outlined) across species. The conserved T180, Q182, and R184 are marked with boxes. RS, arginine-serine rich; ULM, U2AF ligand motif; RRM, RNA recognition motif; UHM, U2AF homology motif. *H. s.: Homo sapiens. M. m.: Mus musculus. D. r.: Danio rerio. D. m.: Drosophila melanogaster. C. e.: Caenorhabditis elegans. A. t.: Arabidopsis thaliana. S. p.: Schizosaccharomyces pombe*. (**B**) *In silico* UAF-1 structure predicted by AlphaFold. RNAH and other motifs are color-coded. (**C**) Detailed structures of AlphaFold-predicted RNAH motifs. Left subpanels: ribbon diagrams of RNAHs (encircled) are shown as an *N*-terminal extension of RRM1s. Segments of RRM1s were labeled based on a solution structure [119]. Middle subpanels: RNAH structures, visualized with stick line sidechains, showing H-bond formation (dashed lines). UAF-1 T180 and R184, and the orthologous residues in other U2AF2 structures, are outlined. Right subpanels: cartoon diagrams of the RNAH motifs. Main chains are represented with spheres and side chains with diamonds. H bonds are indicated with dashed lines (red: main chain bonds; blue: bonds between the main chains and sidechains; yellow: bonds between the sidechains). (**D-G**) Effects of T180I on UAF-1 RNAH conformation as predicted by AlphaFold. T180I caused an obvious left-hand twist at the N-terminus of the helix (**D**, left), larger tilt angles away from the RRM1 β1 axis (**D**, right, and **E**), opposite local twists on helical segments 2 and 4 (**F**), and significant structural deviations (root mean square deviation, RMSD) from that of the wild-type (**G**). Statistics: Student’s t-test. *: *P* < 0.05; **: *P* < 0.01; ***: *P* < 0.001. NS: not significant. (H-K) Effects of T145I on hU2AF2 RNAH conformation as predicted by AlphaFold. Like UAF-1 T180I, hU2AF2 T145I caused similar effects on the twist of the helix (**H**, left), the helical tilt angles (**H**, right, and **I**), local twists on helical segments 2 and 4 (**J**), and RMSD (**K**). Statistics: Student’s t-test. **: *P* < 0.01; ***: *P* < 0.001; ****: *P* < 0.0001. NS: not significant.

*C. elegans* UAF-1 T180 is orthologous to hU2AF2 T145, a residue at the center of the hU2AF2 RNAH motif [26, 42–44]. Among the UAF-1 RNAH residues, T180 (hU2AF2 T145), Q182 (hU2AF2 Q147), and R184 (hU2AF2 R149) are highly conserved (Fig. 2A).

In the absence of a resolved *C. elegans* UAF-1 structure, we examined AlphaFold [84, 85] predicted structures of UAF-1 (Fig. 2B) and U2AF2 proteins from other species. Interestingly, an alpha helix-like structure was identified as an N-terminal extension of U2AF2 RRM1 in all species (Fig. 2C and Supplementary Fig. S4A). A similar alpha-helix was observed in a resolved structure of hU2AF2 in complex with a PPT-mimicking oligonucleotide (Supplementary Fig. S4B) [42]. The predicted align errors (PAE) and pLDDT (predicted Local Distance Difference Test) of the RNAH structures were shown in Supplementary Fig. S5.

It is important to note that the pLDDT scores of residues preceding *C. elegans* UAF-1 T180 (and their corresponding residues in several other orthologs) were below 50 (Supplementary Fig. S5B), suggesting that the predicted conformations of these residues may be unreliable. Furthermore, the roles of hU2AF2 G141 and S142 in RNAH remain unclear, as the conformations of these residues have not been well resolved (Supplementary Fig. S4B) [42]. Therefore, these structural models should be interpreted with caution, especially in regions with low pLDDT scores or residues with unresolved conformations.

More detailed analyses on the AlphaFold-predicted structure showed that the orientations of the sidechains of the RNAH motif were comparable in all species (Fig. 2C and Supplementary Fig. S4A). For example, the sidechains of the highly conserved UAF-1 T180 and R184 (orthologous to hU2AF2 T145 and R149) point to one side of the helix, away from the sidechains of Q182 and R185 (orthologous to hU2AF2 Q147 and R150) (Fig. 2A, C and Supplementary Fig. S4A). Resolved structures showed that hU2AF2 Q147 and R150 can form H bonds with the PPT nucleotides (Supplementary Fig. S4B) [42, 58].

To explore how T180I might affect the structure of RNAH, we analyzed AlphaFold predicted structures of the UAF-1 T180I mutant protein, focusing on the conformation of RNAH (Fig. 2D). In all predicted structures, T180I appeared to induce a left-hand twist at the *N*-terminus of the helix (Fig. 2D) and a larger tilt angle between the axes of the mutant helices and the axis of the RRM1 β1 strand (Fig. 2D, right, and Fig. 2E). Furthermore, differential twists were predicted in the four stable helical segments of the helix (excluding the *N*-terminal G176 and P177 due to their limited contribution to helix stability) (Fig. 2F), and the mutant RNAH exhibited a significant structural deviation from the wild-type (Fig. 2G).

To further explore how UAF-1 T180 might affect RNAH conformation *in silico*, we examined AlphaFold predicted structures of UAF-1 mutant proteins carrying all other T180X mutations (X represents non-threonine amino acid) (Supplementary Fig. S6). Here, each mutation was predicted to induce variable changes in the helix tilt angles (Supplementary Fig. S6A and B), the overall RNAH structures (Supplementary Fig. S6C), and the twists of the four helical segments (Supplementary Fig. S6D–G).

Because experimentally resolved structures are lacking, the AlphaFold-predicted U2AF2 models for *C. elegans* and other species were likely inferred from available human structures. Their resemblance to human U2AF2 structures (also see below) should be interpreted with caution.

### AlphaFold predicts comparable RNAH conformations in human U2AF2

To explore whether hU2AF2 T145 (orthologous to *C. elegans* UAF-1 T180) also affects the RNAH conformation, we examined AlphaFold predicted hU2AF2 structures carrying each T145X mutation, focusing on RNAH (Fig. 2H–K, and Supplementary Fig. S7). These *in silico* analyses predicted that hU2AF2 T145I also induced a left-hand twist at the *N*-terminus of RNAH (Fig. 2H), larger tilt angles between the helical axes and the axis of the RRM1 β1 strand (Fig. 2H and I), twists of the helical segments (Fig. 2J), and different RNAH overall structures compared to wild-type (Fig. 2K). Inspection of the predicted structures of other hU2AF2 T145X mutant proteins showed further similarities between hU2AF2 RNAH and *C. elegans* UAF-1 RNAH. For example, hU2AF2 T145N, T145S, and T145D mutations (Supplementary Fig. S7A–C), and the corresponding *C. elegans* UAF-1 mutations (T180N, T180S, and T180D, Supplementary Fig. S6A–C), all induced minimal changes in the helix tilt angles and the overall structures. However, hU2AF2 R149W (a recurrent NDD-causing mutation) [54, 57] and the orthologous *C. elegans* UAF-1 R184W both induced the formation of the largest helix tilt angles and the most significant structural changes (Supplementary Fig. S6A–C, S7A–C).

### *C. elegans* UAF-1 T180I or human U2AF2 T145I did not obviously affect its binding to *C. elegans* UAF-2 (U2AF1 ortholog) or human U2AF1

RNAH is located at the N-terminus of U2AF2 RRM1 and to the C-terminus of U2AF2 ULM, a position that might affect the interaction between U2AF2 and U2AF1. To test this, we performed pull-down experiments using purified *E. coli*-expressed *C. elegans* UAF-1 (U2AF2 ortholog) and HEK293T-expressed UAF-2 (U2AF1 ortholog). We found that UAF-1 and UAF-2 can pull down each other, with no obvious difference between wild-type UAF-1 and UAF-1 T180I in binding wild-type UAF-2 (Supplementary Fig. S8A). Similarly, hU2AF2 and hU2AF1 expressed in HEK293T cells can immunoprecipitate each other, and no obvious difference was observed between wild-type hU2AF2 and hU2AF2 T145I in binding wild-type hU2AF1 (Supplementary Fig. S8B).

To assess whether *C. elegans* UAF-1 T180I or hU2AF2 T145I affects the conformation of the U2AF heterodimer, we employed AlphaFold predictions. In the five predicted models of the *C. elegans* U2AF heterodimer (Supplementary Fig. S9A and B for model 1, Raw Data_AlphaFold Predictions for models 2-5), the regions of reciprocal tryptophan recognitions [32, 33] were well aligned for both wild-type and mutant complexes (Supplementary Fig. S9C and D), and no obvious conformational differences were observed between the wild-type and mutant models (Supplementary Fig. S9C, RMSD). Like *C. elegans*, the five predicted models of the hU2AF2/hU2AF1 heterodimers were also well aligned in the regions of reciprocal tryptophan recognitions (Supplementary Fig. S9E–H for model 1, Raw Data_ AlphaFold Predictions for models 2-5).

Supporting these observations, the pLDDT scores (color coded) and PAE plots (Supplementary Fig. S10 for models 1, Raw Data_AlphaFold Predictions for models 2–5) indicated that the predicted conformations of the reciprocal tryptophan recognition regions (enclosed in dotted lines) exhibited high reliability in all models of wild-type or mutant U2AF heterodimers.

We observed that the overall domain arrangements within the heterodimer complexes exhibited noticeable variations. It is unclear whether these differences arose from inconsistent AlphaFold predictions or reflected snapshots of the complexes’ dynamic conformations *in vivo*. An attempt to model the U2AF2/U2AF1/RNA tertiary complex did not yield structures with stable conformations for either the RNAH motif or the RNA molecule itself. Thus, the extent to which AlphaFold predictions of the U2AF heterodimer complexes, with or without RNA molecules, accurately represent the *in vivo* structures remains uncertain.

### *uaf-1(T180X)* homozygous mutations differentially affect *C. elegans* phenotypes

Given the broad effects of *C. elegans uaf-1(n4588)(T180I)* on genome-wide alternative splicing and the comparable effects of UAF-1 T180X and hU2AF2 T145X on the predicted RNAH conformations, an important question is how UAF-1 T180X mutations affect the recognition of diverse 3’ss. To address this, we generated a series of *uaf-1(T180X)* knockin mutants and analyzed the phenotypes and genome-wide alternative splicing in homozygous mutants.

Using the CRISPR/Cas9 method (Supplementary Fig. S11), we generated 12 *uaf-1(T180X)* knockin strains (Supplementary Table S1), including *T180D* (Asp), *T180F* (Phe), *T180C* (Cys), *T180G* (Gly), *T180A* (Ala), *T180R* (Arg), *T180S* (Ser), *T180P* (Pro), *T180N* (Asn), *T180M* (Met), *T180L* (Leu) and *T180I* (Ile) (*n4588* genocopy) (Supplementary Table S1). We also isolated three in-frame deletions, *S178delV179L, V179_T180del*, and *Y187del*, and two frameshift mutations, G194Rfs*197 and V179Mfs*181 (Supplementary Table S1), as byproducts. We failed to isolate knockin mutants for seven other amino acids, including H (His), Y (Tyr), E (Glu), W (Trp), V (Val), Q (Gln), and K (Lys). To simplify the description, we called these mutations collectively *T180X* and analyzed homozygotes of each mutation.

The *T180I* knockin mutants exhibited temperature-sensitive (*ts*) lethality like the original *uaf-1(n4588)(T180I)* mutants (Supplementary Table S2) [64]. Interestingly, *T180M* caused a more severe *ts*-lethality while *T180L* caused a less severe *ts*-lethality (Supplementary Table S2). *T180A* caused sterility at all temperatures (Supplementary Table S2).

*T180R, Y187del*, and the two frameshift mutations (Supplementary Table S1) caused early-stage larval arrest (Supplementary Table S2), like *uaf-1(null)* mutants [64].

Surprisingly, nine mutants, including *T180C, T180F, T180D, T180G, T180S, T180P, T180N, S178delV179L* and *V179_T180del*, were grossly normal. These animals survived like wild type at all temperatures (Supplementary Table S2) and locomoted normally except for *T180D* (Supplementary Fig. S12A). In comparison, the *ts* mutants *T180I* and *T180M*, and the sterile mutant *T180A*, exhibited partially reduced locomotion (Supplementary Fig. S12A).

*uaf-1(n4588)(T180I)* was originally isolated as a strong suppressor of the defective locomotion of *unc-93(e1500)* mutants [64, 86, 87]. Aside from the *ts*-lethality, *uaf-1(n4588)(T180I)* also caused partial protruding vulva (Pvl) and sterility (Ste) phenotypes [72]. These phenotypes can be partially suppressed by loss-of-function (lf) mutations in *rbm-5*, which encodes an RNA-binding protein homologous to human RBM5 and RBM10 [74]. In addition, *uaf-1(n4588)(T180I)* and its intragenic suppressor, *uaf-1(n4588 n5127)(M157I, T180I)* [64], were found to shorten the lifespan of *C. elegans* [72]. We therefore assessed these phenotypes in the new mutants to understand how *T180X* affects UAF-1 functions.

We found that the *ts* mutations (*T180I, T180M*, and *T180L*), the sterile *T180A* mutation, and, unexpectedly, the viable *T180C* mutation, all significantly suppressed the defective locomotion and the “rubberband” phenotype of *unc-93(e1500)* mutants, while the other eight viable mutations failed to (Supplementary Fig. S12B, Supplementary Tables S2 and S3). *uaf-1* transgenes carrying the suppressive *T180X* mutations failed to rescue or only partially rescued the suppression of *unc-93(e1500)* by *uaf-1(n4588)(T180I)* (Supplementary Fig. S12C and Supplementary Table S3). All *ts* mutations caused *ts* Pvl (Supplementary Fig. S12E-S12G) and Ste (Supplementary Fig. S12H-S12J) phenotypes correlated with their lethality, and *rbm-5(lf)* partially suppressed the *ts* lethality of these mutants (Supplementary Table S4). Interestingly, *T180C, T180S*, and *V179_T180del* consistently extended the lifespan, whereas *T180I* shortened the lifespan (Supplementary Fig. S12K-S12L), as previously reported [72].

Finally, we investigated how seven other amino acids, for which we failed to isolate *T180X* knockin mutations, affected UAF-1 function by testing *uaf-1* transgenes carrying each mutation in rescuing the suppression of *unc-93(e1500)* by *uaf-1(n4588)(T180I)*. Here, we also tested the *uaf-1(R184W)* mutant transgene. We found that *T180H, T180Y, T180E*, and *T180W* did not obviously impact the transgenes’ rescuing effects (Supplementary Fig. S12D and Supplementary Table S3), while *T180V, T180Q*, and *T180K* abrogated or significantly weakened the rescuing effects (Supplementary Fig. S12D and Supplementary Table S3). Interestingly, *R184W* had a partial effect (Supplementary Fig. S12D and Supplementary Table S3). Taken together, we hypothesize that UAF-1 RNAH may influence diverse biological processes through flexible and context-dependent mechanisms.

### *C. elegans uaf-1(T180X)* mutations induce diverse alternative splicing patterns correlated with specific PPT sequences

To investigate how UAF-1 T180X affects genome-wide alternative splicing, we performed RNA**-**Seq on each *T180X* knockin homozygous mutant at the L4 stage. The heat map of differentially expressed genes highlighted mutation-specific effects on broad gene expression landscapes (Supplementary Fig. S13).

**Figure 3.**
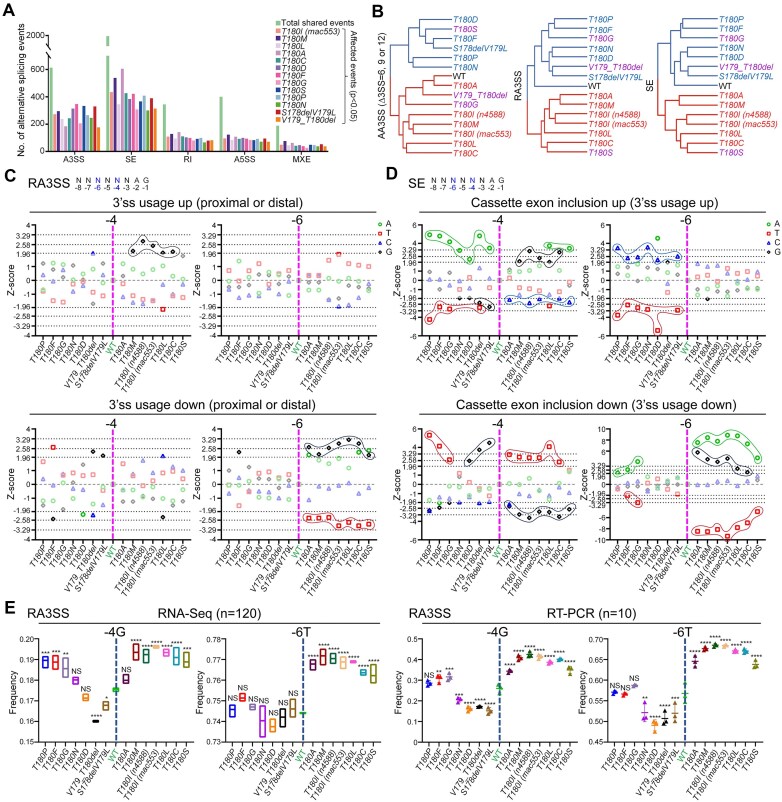
*uaf-1(T180X)* mutations differentially affect genome-wide alternative splicing correlated with specific nucleotides at positions −4 and −6 of 3’ss. (**A**) The number of alternative splicing events affected by each *uaf-1(T180X)* mutation at the L4 larval stage. (**B**) Hierarchical clustering of *uaf-1(T180X)* mutations based on how similarly each mutation affects AA3SS, RA3SS, and SE events. (**C** and **D**) Nucleotide frequency significance at positions −4 and −6 of 3’ss in RA3SS and SE events affected by *uaf-1(T180X)* mutations. Statistics: two-tailed Z-test. ∣Z∣> 1.96: *P* < 0.05; ∣Z∣ > 2.58: *P* < 0.01; ∣Z∣ > 3.29: *P* < 0.001. (**E**) The weighted frequencies of −4G and −6T in 3’ss of RA3SS events in each *uaf-1(T180X)* mutant based on RNA-Seq and RT-PCR results. Statistics: Dunnett’s multiple comparison test with one-way ANOVA. *: *P* < 0.05; **: *P* < 0.01; ***: *P* < 0.001; ****: *P* < 0.0001. NS: not significant.

Focusing on alternative splicing events shared by wild type and the *T180X* mutants, we found that each *T180X* mutation affected a significant number of events (Fig. 3A). Among AA3SS events, the mutations primarily led to increased proximal 3’ss usage (Supplementary Fig. S14A, top three panels). Among RA3SS events, however, the mutations led to more balanced changes in the proximal 3’ss usage (Supplementary Fig. S14A, bottom panel). The representative individual events highlighted dynamic splicing changes in a mutation-dependent manner (Supplementary Fig. S14B), and in some cases, *e.g. nsy-1* and *sipa-1*, one group of mutations (*T180I, T180M, T180L, T180A*, and *T180C*) induced changes opposite to those by another group of mutations (*T180D, T180F, T180P, T180N* and *S178delV179L*) (Supplementary Fig. S14B).

*T180X* mutations affected smaller fractions of SE events (Supplementary Fig. S14C), with individual events displaying mutation-specific changes and occasionally, opposite changes between mutations (Supplementary Fig. S14D). We further validated the RNA-Seq results by performing RT-PCR analysis on multiple alternative splicing events, including AA3SS (Supplementary Fig. S15A), RA3SS (Supplementary Fig. S15B), SE (Supplementary Fig. S16A), RI (Supplementary Fig. S16B), and MXE events (Supplementary Fig. S16C). Therefore, we hypothesize that RNAH might act as a “swing” to modulate UAF-1 recognition of 3’ss.

Based on how similarly they impacted genome-wide alternative splicing, *T180X* mutations were classified into two major groups: the group of *T180I, T180C, T180L, T180M*, and *T180A* (*T180I*-like, hereafter) and the group of *T180D, T180F, S178delV179L, T180P*, and *T180N* (*T180D*-like, hereafter) (Fig. 3B). The association of *T180S, T180G*, or *V179_T180del* was dynamic, depending on alternative splicing events (Fig. 3B). The splicing-based classification correlated well with the phenotypes of the mutants (compare Fig. 3B with Supplementary Fig. S12, and Supplementary Tables S2–S4), implying the biological significance of the mutation-induced splicing changes.

To understand how *uaf-1(T180X)* impacted the recognition of different 3’ss, we analyzed the frequency of each nucleotide at positions −3 to −8 of 3’ss in RA3SS and SE events affected by each *T180X* mutation (Fig. 3C, D, Supplementary Fig. S17A and S17B).

In RA3SS events, a higher −4G frequency was significantly associated with increased 3’ss usage, regardless of proximal or distal sites in most *T180I*-like mutants (Fig. 3C, top left panel, enclosed), while a higher −6G frequency and a lower −6T frequency were significantly associated with decreased 3’ss usage in all *T180I*-like mutants (Fig. 3C, bottom right panel, enclosed). Alternatively, a higher −5A frequency and/or a lower −5T frequency were associated with increased 3’ss usage in a few *T180D*-like mutants (Supplementary Fig. S17A).

The association pattern between PPT nucleotide frequencies and the affected SE events was noticeably more complex (Fig. 3D and Supplementary Fig. S17B). Nevertheless, there were interesting resemblances between SE and RA3SS events. For example, a higher −4G frequency in 3’ss was significantly associated with increased cassette exon inclusion in most *T180I*-like mutants (Fig. 3D, top left panel, enclosed), a higher −6G frequency and a lower −6T frequency were significantly associated with decreased cassette exon inclusion in nearly all *T180I*-like mutants (Fig. 3D, bottom right panel, enclosed), and a higher −5A frequency and/or a lower −5T frequency were strongly associated with increased cassette exon inclusion in nearly all *T180D*-like mutants (Supplementary Fig. S17B, enclosed).

We further calculated the weighted frequency of each nucleotide at various positions of 3’ss, regardless of proximal or distal 3’ss, in RA3SS events (Fig. 3E and Supplementary Fig. S18A). Here, the weighted frequencies of −4G and −6T based on RNA-Seq were consistent with RT-PCR-validated events, showing that −4G and −6T were significantly favored in nearly all *T180I*-like mutants (Fig. 3E). Using a similar approach, we identified additional nucleotides at positions −3, −5, −7, and −8 in RA3SS events associated with *T180I*-like and *T180D*-like mutants (Supplementary Fig. S18A, enclosed, and Supplementary Fig. S18B for simplified bidirectional associations).

As mentioned above, WebLogo analyses showed that intronic nucleotides upstream of position -7 of 3’ss were not or only weakly associated with alternative splicing in *uaf-1(n4588)(T180I)* mutants. To examine this in more detail, we calculated the weighted frequency of each nucleotide from positions −9 to −20 (Supplementary Fig. S19 and S20) (positions −7 and −8 were shown in Supplementary Fig. S18). Here, it appeared that −9A, −10A, −11G, −14A, and −15A were associated with decreased usage of 3’ss in the *T180I*-like mutants, while −10C and −15T were associated with increased usage of 3’ss in these mutants.

Taken together, we hypothesize that nucleotides around positions −4 to −6 are significantly associated with 3’ss usage in *T180X* mutants, while nucleotides at other positions might also be involved.

### Molecular dynamics simulations predict differential binding to a 3’ss RNA by T180 or I180-induced conformational change in *C. elegans* UAF-1 RNAH motif

**Figure 4.**
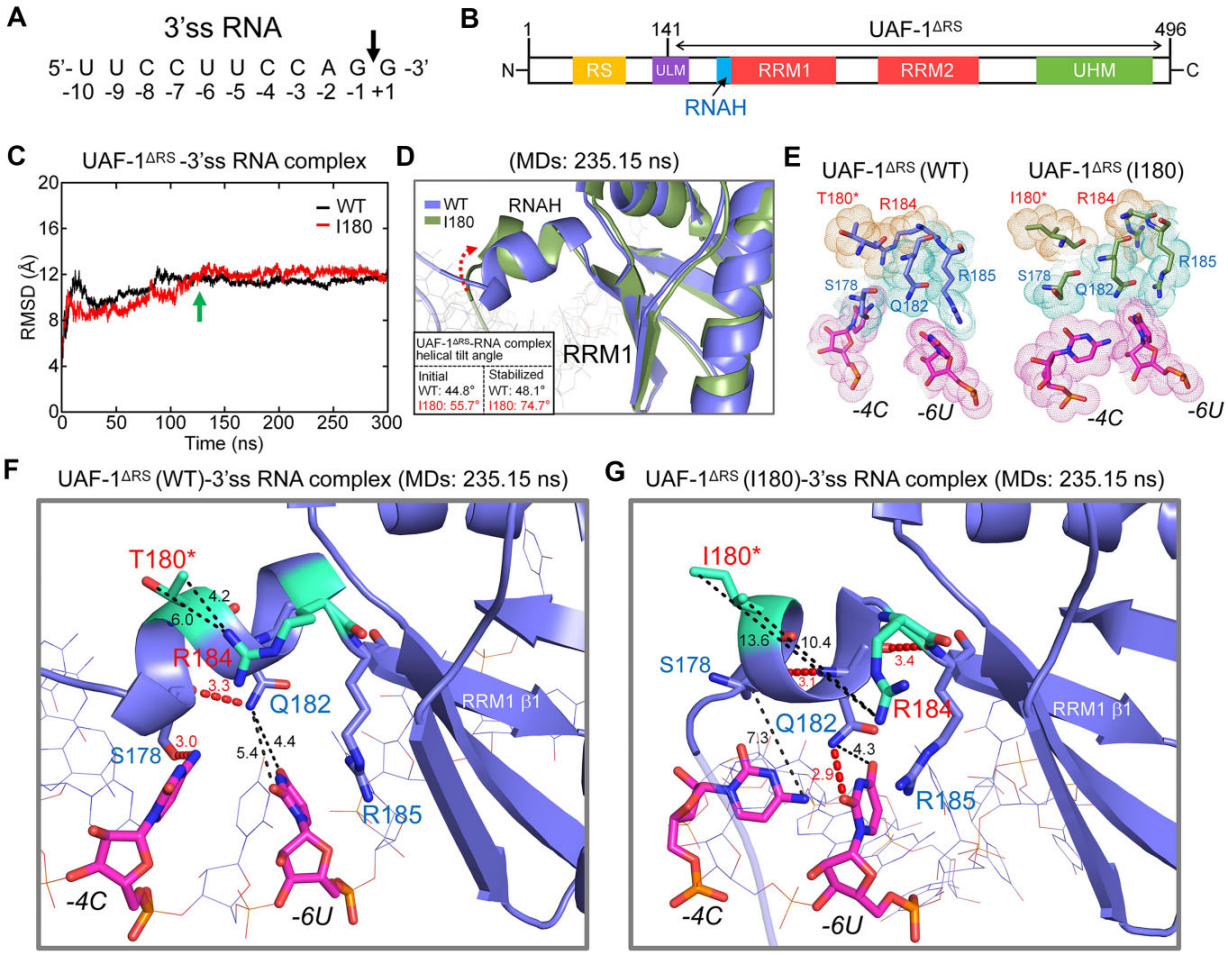
Molecular dynamics simulations (MDs) predict the dynamic structural difference between the UAF-1(I180)–RNA complex and the UAF-1(WT)-RNA complex. (**A**) Sequence of the PPT-AG 3'ss RNA molecule. The arrow indicates the intron-exon junction. (**B**) Diagram showing the truncated UAF-1 protein (residues 141–496, UAF-1^ΔRS^) lacking the *N*-terminal RS domain and a portion of the ULM domain. (**C**) RMSDs of the UAF-1^ΔRS^(WT)-RNA and UAF-1^ΔRS^(T180I)-RNA complexes during a 300 ns MD. The arrow indicates the stage of simulation at which a stable conformation appears to be achieved. (**D**) Alignment of the ribbon structures of UAF-1^ΔRS^(WT) RNAH and UAF-1^ΔRS^(I180) RNAH at 235.15 ns of MDs. The RNAH helical tilt angles in the initial complexes and the stabilized complexes are indicated. (**E**) Molecular surface interfaces of the RNAH(WT)–RNA complex and RNAH(I180)–RNA complex at 235.15 ns of MDs. (**F, G**) Ribbon and stick structures of the complexes highlighting the RNAH regions at 235.15 ns of an MD. Key nitrogen and oxygen atoms are shown in blue and red, respectively, and residue 180 and R184 are highlighted in cyan. The distances between atoms are labeled. Strong H bonds are indicated by thick dashed lines and an atomic distance less than 3.5 Å. The distances between atoms potentially involved in the interaction between the RNAH motif and the RNA molecule are indicated with thin dashed lines.

To explore the potential structural mechanism underlying how UAF-1 T180I might affect the binding to 3’ss, we resorted to a molecular dynamics simulation (MDs) method (www.gromacs.org) to model the binding of the UAF-1 wild-type and I180 mutant proteins to a 3’ss RNA molecule (Fig. 4 and Supplementary Fig. S21).

We utilized AlphaFold to model the structure of UAF-1 in complex with a 3’ss RNA molecule, focusing on the binding to the PPT. We first tested an RNA molecule with the more consensus 3’ss sequence UUCCUU**U**CAGG, in which the splice acceptor is underlined, and the nucleotide at position -4 is highlighted in bold. However, this molecule failed to establish stable docking conformations with UAF-1. When -4U was changed to -4C, the RNA molecule docked stably. Hence, subsequent MDs were performed using UUCCUU**C**CAGG (Fig. 4A) as the 3’ss RNA sequence.

To simplify the MDs process, we used a truncated UAF-1 protein (named UAF-1^ΔRS^) lacking the disordered N-terminal RS domain and a portion of the ULM domain (Fig. 2B and 4B). UAF-1^ΔRS^ spans from V141 (hU2AF2 V94) to the C-terminus of UAF-1 (Fig. 4B), encompassing the complete RNAH, RRM1, RRM2, and UHM motifs (Fig. 4B).

We conducted three replicates of 300 ns MDs on the binding of UAF-1^ΔRS^ to the RNA molecule (Fig. 4 and Supplementary Fig. S21). Root mean squared deviations (RMSD) of the UAF-1^ΔRS^(WT)-RNA complex and the UAF-1^ΔRS^(I180)-RNA complex indicated that the structures were stabilized after ∼125 ns during the simulations (Fig. 4C, also shown as Supplementary Fig. S21A for comparison purpose, Supplementary Fig. S21C and S21E), and in two of the three simulations, the stabilized mutant complex adopted a structure highly similar to that of the wild-type complex (Fig. 4C and Supplementary Fig. S21C).

Using snapshots taken at 235.25 ns from replicate 1 of the MDs, we performed structure alignment of the wild-type and I180 RNAH motifs. Compared to the initial conformations of the UAF-1^ΔRS^-RNA complex, the stabilized complexes established larger tilt angles for both the wild-type and I180 protein, with the change in the mutant protein being more dramatic (Fig. 4D). The simulated molecular surface of the wild-type and mutant RNAH-RNA interfaces (Fig. 4E), and the corresponding ribbon-stick structures (Fig. 4F and G) showed that compared to the wild type (Fig. 4E left panel, Fig. 4F), the sidechain of R184 turned away from the sidechain of I180 in the mutant complex (Fig. 4E right panel, Fig. 4G).

Though predicative, the MDs may provide a window into the structural details of UAF-1 bound to a PPT RNA molecule when X-ray or NMR structures are lacking. To examine the details, we partitioned the entire simulation trajectory into 50-ps intervals, yielding a total of 6, 000 segment slices per complex. Analyses of the slices suggested that the sidechain guanidium group of UAF-1 R185 (hU2AF2 R149) tended to form H bonds with -7C of the RNA molecule, and the sidechain of UAF-1 Q182 (hU2AF2 Q147) tended to form H bonds with -7C and -6U (Supplementary Fig. S21B, D, and F). Variable H bonds were also formed between UAF-1 P177, S178 or Q182 (comparable to hU2AF2 S142, Q143 or Q147) with -5U, -4C, or -3C (Supplementary Fig. S21B, D, and F).

Interestingly, the MDs predicted comparable H-bond formation between the RNA molecule and the RNAH residues in both wild-type and mutant complexes (Supplementary Fig. S21G). However, Q182 formed obviously more H bonds in the mutant UAF-1 I180-RNA complex compared to the wild-type complex (Supplementary Fig. S21H), and an imbalance in H bond formation on positions -6 and -4 of the RNA molecule became obvious in the mutant complex (Supplementary Fig. S21I).

### Nucleotide substitutions at positions -6 and/or -4 of 3’ss alter the splicing patterns of reporter genes in a genotype-dependent manner

The MD's predictions, together with the observed associations of splicing patterns with specific nucleotides at positions -6 and -4 of 3’ss, raise the question whether changes at position -6 or -4 may influence UAF-1 binding to 3’ss RNA or alternative splicing events *in vivo*.

To examine whether UAF-1 T180I affects RNA binding, we co-incubated purified UAF-1 WT or T180I proteins with a biotin-labeled 3’ss RNA (based on the distal 3’ss of the *unc-93* reporter, see below) (Supplementary Fig. S22A). Native PAGE showed that UAF-1(WT) and UAF-1 T180I formed comparable complexes with the consensus RNA (Biotin-R1, consensus). However, with a -6U to -6A substitution in the RNA (Biotin-R2, non-consensus), UAF-1 T180I reduced the binding by approximately 50% (Supplementary Fig. S22B and S22C). Interestingly, the non-consensus RNA produced a distinct shift pattern compared to the consensus RNA in complexes formed with UAF-1(WT) or UAF-1 T180I (Supplementary Fig. S22B).

**Figure 5.**
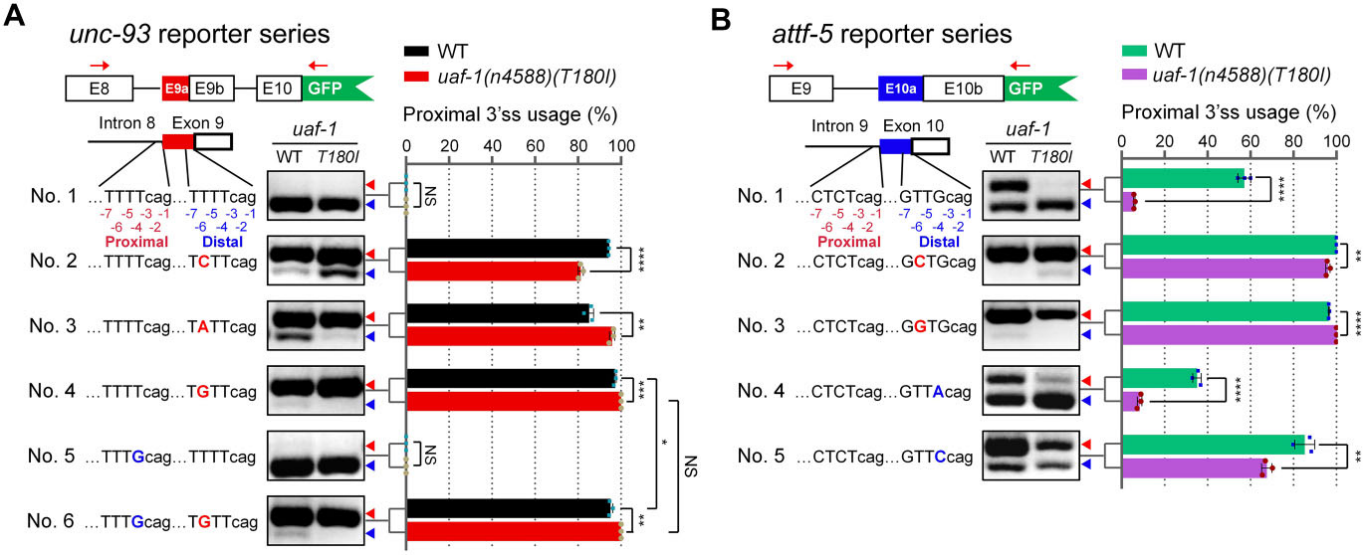
3'ss nucleotides at positions -6 and/or -4 of transgene reporters modulate splicing patterns in a UAF-1 T180-dependent manner. (**A**) Structure of the *unc-93* splicing reporters (left panel, top). PCR primers for detecting splicing isoforms are indicated as arrows. 3’ss sequences of the reporters (No. 1 to No. 6) are shown (left panel). Mutations at positions -6 and -4 are highlighted. RT-PCR products of the reporters were analyzed on a 2.5% agarose gel (mid panel. Upper arrowhead: proximal 3’ss splice isoform; lower arrowhead: distal 3’ss splice isoform). Quantifications of proximal 3’ss usage (right panel) were based on three biological replicates. Statistics: Student’s t-test. **: *P* < 0.01; ***; *P* < 0.001; ****: *P* < 0.0001; NS: not significant. (**B**) Structure of the *attf-5* splicing reporters (left panel, top). PCR primers for detecting splicing isoforms are indicated as arrows. 3’ss sequences of the reporters (No. 1 to No. 5) are shown (left panel). Mutations at position -6 and -4 are highlighted. RT-PCR products of the reporters were analyzed on a 2.5% agarose gel (mid panel. Upper arrowhead: proximal 3’ss splice isoform; lower arrowhead: distal 3’ss splice isoform). Quantifications of proximal 3’ss usage (right panel) were based on three biological replicates. Statistics: Student’s t-test. **: *P* < 0.01; ***: *P* < 0.001; ****; *P* < 0.0001; NS: not significant.

To examine if nucleotides at positions -6 and/or -4 of 3’ss affect the pattern of alternative splicing, we generated an RA3SS reporter carrying the sequence TTTTCAG (positions -7 to -1) at both proximal and distal 3’ss (Fig. 5A, No. 1), using the surrounding sequence of a previously described *unc-93* reporter [64]. In both wild-type and *uaf-1(n4588)(T180I)* mutants, splicing occurred exclusively at the distal 3’ss (Fig. 5A, No. 1). Changing -6T at the distal 3’ss to -6C (Fig. 5A, No. 2), -6A (Fig. 5A, No. 3) or -6G (Fig. 5A, No. 4) strongly shifted splicing to the proximal 3’ss in the wild type and mutants, though the precise effects differed by the genotype (Fig. 5A). In contrast, further changing -4T at the proximal 3’ss in either the No. 1 reporter (Fig. 5A, No. 5) or the No. 4 reporter (Fig. 5A, No. 6) did not obviously or only weakly alter the splicing pattern.

To further examine the effects of position -4, we constructed a second RA3SS reporter (Fig. 5B, No. 1) based on the *attf-5* gene, which exhibited opposite splicing patterns in the wild type and *uaf-1(n4588)(T180I)* mutants (Supplementary Fig. S15). In reporter No. 1, the splicing was balanced at the proximal and distal 3’ss in the wild type but shifted predominantly to the distal 3’ss in *uaf-1(n4588)(T180I)* mutants (Fig. 5B, No. 1). Changing -6T at the distal 3’ss in reporter No. 1 to -6C (Fig. 5B, No. 2) or -6G (Fig. 5B, No. 3) significantly shifted the splicing to the proximal 3’ss in both genotypes. However, changing -4G at the distal 3’ss in reporter No.1 to -4A (Fig. 5B, No. 4) shifted the splicing moderately to the distal 3’ss in the wild type without an obvious effect in *uaf-1(n4588)(T180I)* mutants, whereas to -4C (Fig. 5B, No. 5) shifted the splicing moderately to the proximal 3’ss in both genotypes.

Previously, without knowledge of the RNAH structural prediction, we proposed that -4G promotes splicing at the 3’ss in *uaf-1(n4588)(T180I)* mutants based on analyses of two RA3SS transgenic reporters [64, 71]. With a better structural understanding, we generated additional *unc-93* splicing reporters and confirmed that nucleotide changes at position -6 or -4 can differentially affect the splicing at proximal or distal 3’ss, depending on the genotype (Supplementary Fig. S22D). Together, these reporter assays support the structural predictions from the MDs as well as the transcriptome analyses.

### Human alternative splicing is affected by mutations in hU2AF2 RNAH

The *hU2AF2(T145I)* mutation, orthologous to *uaf-1(T180I)*, was a causal mutation of an NDD patient [57]. To investigate whether hU2AF2 T145I affects alternative splicing, we introduced plasmids encoding hU2AF2 T145I mutant proteins and two alternative splicing reporter plasmids to HEK293T cells. The recurring NDD-causing *hU2AF2(R149W)* mutation was also tested.

**Figure 6.**
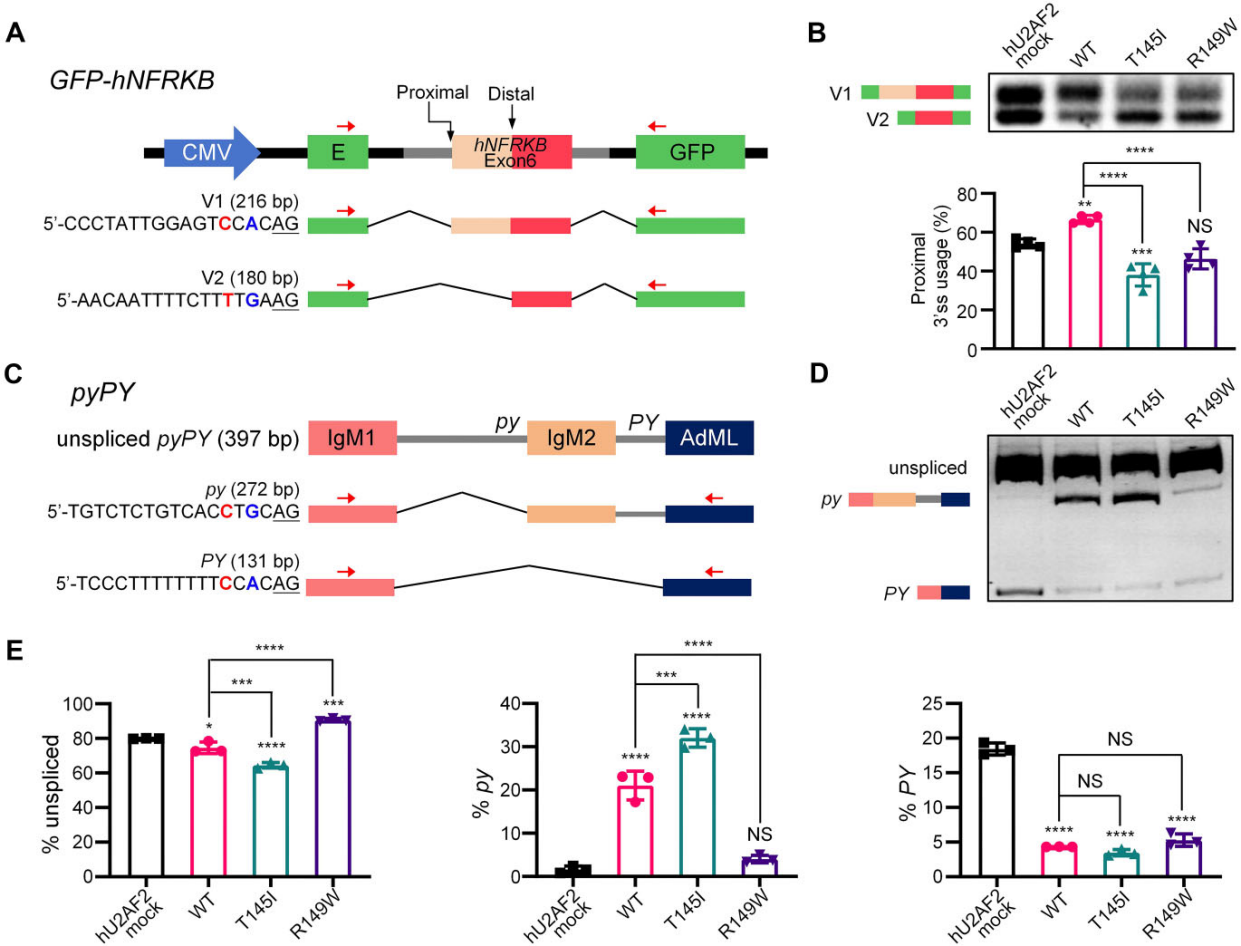
A conserved function of the hU2AF2 RNAH motif in modulating 3'ss selection. (**A**) Structure of the *GFP-hNFRKB* minigene splicing reporter and the expected alternative splicing events. PCR primers for detecting splicing isoforms are shown as arrows. The lengths of the splice isoforms and the sequences of the proximal and distal 3’ss are shown on the left. CMV: CMV promoter. E: EGFP N-terminal fragment. (**B**) RT-PCR products of the *GFP-hNFRKB* reporter co-transfected with *hU2AF2* expression constructs in HEK293T cells were analyzed on a 2.5% agarose gel (top panel). Quantifications of *GFP-hNFRKB* splice isoforms (bottom panel) were based on four biological replicates. Statistics: Tukey’s multiple comparison test with one-way ANOVA. **: *P* < 0.01; ***: *P* < 0.001; ****: *P* < 0.0001. NS: not significant. (**C**) Structure of the *pyPY* minigene splicing reporter based on a previous description [47]. PCR primers for detecting splicing isoforms are shown as arrows. The expected lengths of the splice isoforms and sequences of the upstream and downstream 3’ss are shown on the left. (**D**) PAGE separation of RT-PCR products of the *pyPY* reporter co-transfected with *hU2AF2* expression constructs in HEK293T cells. (**E**) Quantifications of the *pyPY* splice isoforms. Results were based on three biological replicates. Statistics: Tukey’s multiple comparison test with one-way ANOVA. *: *P* < 0.05; ***: *P* < 0.001; ****: *P* < 0.0001. NS: not significant.

We found that *hU2AF2(WT)* significantly increased the proximal 3’ss usage of an *hNFRKB* RA3SS reporter transcript (Fig. 6A) (see Materials and Methods) compared to *hU2AF2* mock (empty expression vector) transfected cells (Fig. 6B). However, *hU2AF2(T145I)* and *hU2AF2(R149W)* both decreased the proximal 3’ss usage of the transcripts, though the effect of *hU2AF2(R149W)* appeared to be slightly weaker (Fig. 6B).

Interestingly, *hU2AF2(WT), hU2AF2(T145I)*, and *hU2AF2(R149W)* exerted more diverse effects on the splicing of the *pyPY* reporter [47] (Fig. 6C). Compared to mock-transfected cells, *hU2AF2(WT)* reduced the proportion of the unspliced isoform, *hU2AF2(T145I)* further reduced it, while *hU2AF2(R149W)* weakly increased it (Fig. 6D and 6E). The difference correlated well with the differential effects of *hU2AF2* on the spliced isoforms: *hU2AF2(WT)* and *hU2AF2(T145I)* (to a larger degree) significantly increased the portion of the *py* isoform, whereas *hU2AF2(R149W)* did not (Fig. 6D and E). However, all three transgenes significantly reduced the *PY* isoform (Fig. 6D and E). It is noteworthy that *GFP-hNFRKB* and *pyPY* both had a -4G at the distal 3’ss and the *py* 3’ss, respectively, and *hU2AF2(T145I)* increased their usage compared to *hU2AF2(WT)*. Hence, we propose that hU2AF2 T145 plays a conserved role in modulating alternative splicing.

## Discussion

In this study, we uncovered a conserved role of *C. elegans* UAF-1 T180 in modulating genome-wide alternative splicing. We performed *in vivo* amino acid scanning on UAF-1 T180 to assess the effects on genome-wide alternative splicing, applied *in silico* methods to model the RNAH motif and to predict how UAF-1 might dynamically interact with PPT RNA, and supported the *in silico* prediction through gel shift experiments and mutagenesis analyses of transgenic splicing reporters. Based on these findings, we hypothesize that a flexible U2AF2 RNAH motif may adjust its conformation when contacting 3’ss and, together with other domains of U2AF2, U2AF1 and potentially more splicing factors, generate a coordinated recognition of diverse 3’ss.

### Developmental regulation of AA3SS events

Most AA3SS events had ∆3SS equal to multiples of three. Amino acids encoded by the extra nucleotides between the alternative 3’ss are often conserved in nematodes [68]. Indeed, the AA3SS events exhibiting decreased proximal 3’ss usage from wild-type eggs to L4 larva are encoded by genes enriched in GO (Gene Ontology) processes such as cellular component organization, development and lifespan, and in KEGG (Kyoto Encyclopedia of Genes and Genomes) pathways such as mTOR, autophagy and longevity (Supplementary Fig. S23A), supporting functional importance of the splicing changes. At the L4 stage, *uaf-1(n4588)(T180I)* significantly affected these AA3SS events (Supplementary Fig. S23B), consistent with the developmental and lifespan defects in the mutants.

We observed that the proximal PPTs of AA3SS events, affected or not by *uaf-1(n4588)(T180I)*, did not exhibit a distinct consensus sequence. Previous analyses of alternative splicing in germline and adult somatic tissues discovered a similar feature of proximal PPTs in AA3SS events [68]. However, the proximal PPTs of RA3SS events exhibited distinct consensuses, depending on how they were affected by *uaf-1(n4588)(T180I)*. Therefore, AA3SS and RA3SS probably represent subtypes of A3SS events involving distinct mechanisms for selecting alternative 3’ss.

AA3SS events are widely expressed across species and may carry out important biological functions [68, 88–91] (this study). However, how alternative 3’ss in these events are selected remains to be understood [68, 90, 92]. Previous studies suggest that the selection of alternative 3’ss may be governed by two distinct yet coexistent mechanisms, splice site scanning or wobbling usage [93–96]. These mechanisms are further influenced by factors such as branch point sequences, the PPTs, intronic or exonic elements, the nucleotides surrounding the splice acceptor, the distance (∆3SS) between alternative splice acceptors, and the tissue types [68, 90, 93–98].

The distinct effects of *uaf-1(WT)* and *uaf-1(n4588)(T180I)* on the selection of proximal 3’ss in AA3SS events at the embryonic and L4 stages suggest development-associated mechanisms. In embryos, the preferred selection of the proximal 3’ss (∼70-90%), which lack a PPT consensus, is consistent with the scanning model. Otherwise, the distal 3’ss, which have a strong PPT consensus, should be preferred or at least equally recognized were UAF-1 able to wobble between the two 3’ss. In the L4 larva, however, the usage of the proximal 3’ss was reduced to ∼20-40%, potentially due to a wobbles selection by UAF-1(WT) between the non-consensus proximal and the consensus distal PPT. Compared to wild type, UAF-1 T180I increased the usage of proximal 3’ss to ∼40-50% at the L4 stage, further supporting a wobbles selection mechanism because T180I likely altered UAF-1 binding to the consensus PPT.

Multiple AA3SS events described in our study have been previously identified to exhibit decreased proximal 3’ss usage from the germline to somatic tissues [68] (Supplementary Fig. S23C). Interestingly, Ragle *et al.* suspected that UAF-1 was potentially involved in the process [68]. A recent study by the same group found that loss of functions in splicing factors DDX41/SACY-1 and PRP22/MOG-5 caused increased proximal 3’ss usage in AA3SS events [99]. DDX41 is a conserved DEAD-box RNA helicase affecting RNA splicing and found to be associated with the myelodysplastic syndrome and innate immunity [100, 101]. PRP22 is also a DEAD-box RNA helicase required for the second step of RNA splicing, capable of contacting 3’ss and modifying the selection of alternative 3’ss [102–106]. Therefore, it appears that the determination of the final 3’ss probably involves a two-step process: the binding of UAF-1 to 3’ss before and the selection between alternative 3’ss by PRP22 after the first step of splicing. Consistent with the genetic findings in *C. elegans*, disrupting multiple human spliceosome C* complex proteins involved in the second step of splicing can increase or decrease the usage of proximal 3’ss of the NAGNAG type of AA3SS events, potentially by interfering with the scanning of the PPT from the branch point to the 3’ splice acceptor [107]. Hence, it will be interesting to investigate the biological significance of the two-step recognition of 3’ss in AA3SS events.

### UAF-1 may dynamically regulate RA3SS and SE events through PPT nucleotide variations around positions -4 and -6 of 3'ss

Alternative splicing is regulated by multiple *cis* and *trans* factors [108]. The consensus 3’ss sequence across species is 5’-(Y)_11_NYAG/G-3’ [23] (splice acceptor underlined; “/” indicates intron-exon boundary). Specifically, the nucleotides from -3 to -13 show different degrees of variations, among which the nucleotides at -4 position are most variable (no consensus). *C. elegans* has a much shorter 3’ss consensus as TTTTCAG and only ∼26% of all 3’ss are exactly TTTTCAG, with nucleotides at positions -3 to -7 exhibiting different degrees of variations [69, 70, 109, 110]. A consensus octamer RNA (TTTTCAG/R, R: purine) binds the *C. elegans* U2AF heterodimer strongly, and substituting the T at positions -3 to -6 with other nucleotides can significantly impact the binding in a sequence-dependent manner [24]. These *in vitro* binding results are consistent with our *in vivo* findings that nucleotides around positions -4 to -6 are important signals for splicing.

Analysis of *uaf-1(T180X)* mutants provided a more layered view than *uaf-1(n4588)(T180I)* alone of how nucleotides at different positions affect splicing. *uaf-1(T180I-like)* mutations significantly favored -4G and -6T (Supplementary Fig. S18). In contrast, *uaf-1(T180D-like)* mutations showed no strong groupwise preference for specific PPT nucleotides, except for disfavoring -5T (Supplementary Fig. S18). Notably, *uaf-1(T180X)* mutations were associated with more complex PPT sequences across positions -3 to -8 in SE events. Together, these findings suggest that nucleotides around positions -4 and -6 are important signals that can influence how RA3SS and SE events are expressed. Splicing reporter analyses confirmed the predictions for RA3SS events (this study) [64, 71].

The branch points in most *C. elegans* introns have not been mapped. Based on available experimental evidence and sequence analyses [68, 69], they are likely located around position -20. It remains unclear how sequences between positions -8 and -19, lying between the presumptive branch points and the short consensus PPT, contribute to UAF-1 binding of PPT, and how the two RRMs of UAF-1 are involved. We found that -9A, -10A, -11G, -14A, and -15A were negatively associated with splicing in *uaf-1(T180I-like)* mutants, whereas -10C and -15T were positively associated. Given that reducing the strength of the *AdML* PPT at positions -8 to -11 generally reduces hU2AF2 binding [26], that the hU2AF heterodimer shifts the “open” or “close” conformation depending on strong or weak 3’ss [39, 40], and the hU2AF2 RRM2-linker-RRM1 region is primarily involved in PPT binding, together we suspect that the two RRM motifs of *C. elegans* UAF-1 and the intronic sequences upstream of the consensus PPT may play a similar role.

### The RNAH motif probably recognizes PPT sequences via an induced-fit mechanism

Building on previous functional and structural studies of U2AF2, Glasser *et al.* recently proposed that U2AF2 may act as a sensitive “rheostat” for splicing [26]. Our findings, combining *in vivo* evidence with *in silico* modeling, suggest that RNAH may be an important regulatory element underlying this dynamic function.

The *in silico* analyses aligned with resolved structures in some respects. For example, crystal structures showed that H bonds are formed between the sidechains of hU2AF2 T145 and R149 (Supplementary Fig. S24), and hU2AF2 R150 and Q147 formed H bonds with 3’ nucleotides of PPT-like oligonucleotides (Supplementary Fig. S4B) [42, 44, 58]. An NMR solution structure of hU2AF2 (PDB: 6TR0) also showed H-bond formation between the sidechains of hU2AF2 T145 and R149 [43]. Similarly, AlphaFold predicted analogous H bond formation by the sidechains of *C. elegans* UAF-1 T180 and R184, and the MDs predicted that the side chains of *C. elegans* UAF-1 R185 and Q182 formed H bonds with -7C and/or -6U of the RNA.

The MDs of the UAF-1(I180)-RNA complexes predicted stronger H bonds at -6U of the RNA and weaker H bonds at -4C, compared to the wild-type complex. This shift is consistent with the favoring of -6T and/or disfavoring of -4C in *uaf-1(n4588)(T180I)* mutants in the transcriptome analyses. Given that RNA molecules adopt flexible conformations in the U2AF2-RNA complex [26], conformational changes of the mutant RNAH probably underlie these differences in H-bond formation. Although the splicing reporter analyses further support the MD's prediction and transcriptome results, these structural findings should be interpreted with caution, as the simulation was based on a pre-built UAF-1-RNA complex model from AlphaFold, and their accuracy requires further experimental validation.

Among all the T180X substitutions, T180Y, T180W, and T180F formed an intriguing group, as these mutants were wild-type-like. However, in most predicted structures, the aromatic sidechains of Y180, W180, and F180 were positioned away from the sidechain of R184, leading to dramatic RNAH conformation changes (Supplementary Fig. S6). We suspect this discrepancy between structure and function reflects inaccuracy in the predicted models. In reality, the cationic sidechain of R184 and the aromatic sidechain of the mutant residue 180 (Y, W, or F) are well positioned to form the cation-π interaction, a type of non-covalent atomic interaction previously discovered in other α-helices [111, 112]. Thus, we suspect that genuine cation-π interactions might be formed between UAF-1 Y180, W180, or F180 and R184 *in vivo*, thereby supporting RNAH conformation for wild-type-like UAF-1 activities. This scenario again highlights the need for caution in interpreting the biological significance of the *in silico* structural predictions.

**Figure 7.**
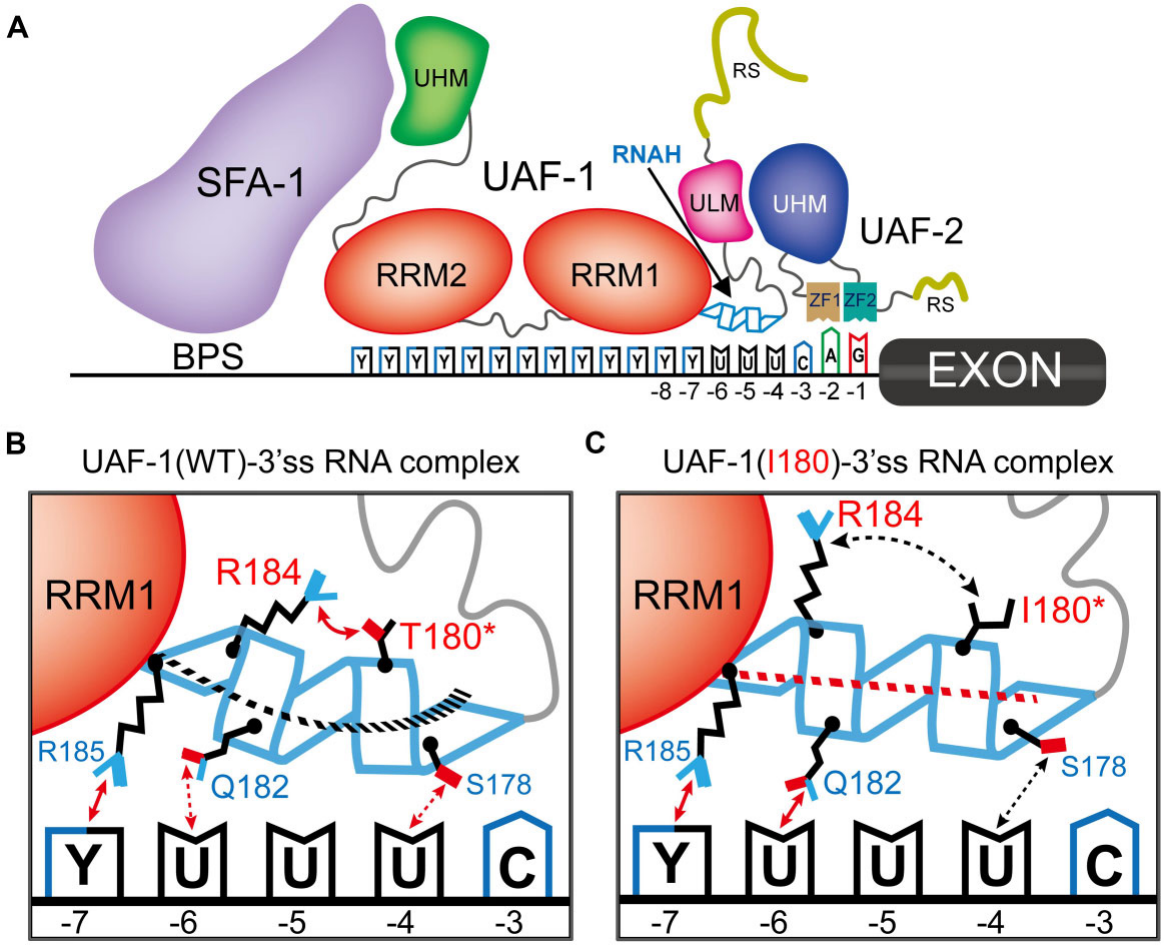
A hypothesized model illustrating the recognition of key 3’ss sequence features in *C. elegans*. (**A**) SFA-1 (SF1), UAF-1 (U2AF2), and UAF-2 (U2AF1) form a tertiary complex to bind the branch point sequence, the PPT, and the AG splice acceptor, respectively. UAF-1 RRM2 and RRM1 and their linker domain bind the upstream PPT nucleotides, while the RNAH motif binds the nucleotides at and around positions -4 to -6 of the downstream PPT nucleotides. (**B, C**) Detailed illustrations of the binding of UAF-1 wild-type or I180 RNAH motif to the PPT. In the wild-type, the sidechains of T180 and R184 are positioned closely, potentially forming flexible H bonds as shown in Figure 2C. These bonds help adjust the sidechain orientations of S178 and Q182 for binding diverse PPT nucleotides near the splice acceptor through an induced-fit mechanism. In the mutant complex, the sidechain of I180 reorientates away from the sidechain of R184, generating abnormal twists in the helix that reposition the sidechain of S178 away from, while bringing the sidechain of Q182 closer to, the PPT nucleotides. This conformational change favors binding to -6U but disfavors binding to -4U. In both illustrations, red solid lines and dashed arrowheaded lines indicate likely and potential H bond formation, respectively, while black dashed arrowheaded lines indicate unlikely or no H bond formation.

Taken together, we hypothesized a model for the UAF-1 RNAH motif (Fig. 7). In this model, the SFA-1-UAF-1-UAF-2 (orthologous to human SF1-U2AF2-U2AF1) ternary complex coordinates the recognition of 3’ss through the binding of the branch point by the SF1 KH fold, of the upstream PPT by the UAF-1 RRM motifs, and of the splice acceptor “AG” by the U2AF1 zinc fingers (Fig. 7A). Specifically, upstream PPT sequences are dynamically recognized by the RRM2, RRM1, and their linker region, whereas downstream PPT sequences close to the splice acceptor are dynamically recognized by RNAH (Fig. 7A). The interaction between T180 and R184 (Fig. 7B) may adjust the conformation of RNAH by twisting the helix, reorientating the sidechains of Q182 and potentially other RNAH residues to generate an induced-fit interaction with PPT nucleotides. Disrupting the interaction between T180 and R184, e.g. through T180I and other T180X mutations, could alter the conformation of RNAH and impact the PPT recognition (Fig. 7C).

Consistent with this model, the NDD-causing hU2AF2 R149W mutation was recently shown to alter the orientations of the sidechains of Q147 and R146, replacing the R146-nucleobase bond with a Q147-nucleobase bond [58]. The gel shift experiments suggest that *C. elegans* UAF-1 T180I obviously reduced UAF-1 binding to a non-consensus 3’ss RNA. Moreover, including residues 88-147 (note: human RNAH spans residues 141-149) to an hU2AF2 RRM1/RRM2 recombinant protein significantly increased its binding to PPT RNA [39], supporting a role for hU2AF2 RNAH in facilitating the binding. The partial loss of function caused by *C. elegans* UAF-1 R184W mutation, together with the altered splicing caused by hU2AF2 R149W in human cells, provided further *in vivo* and *ex vivo* evidence for the importance of RNAH in 3’ss recognition.

A previous study reported that in addition to interacting with the U2AF2 ULM domain, the U2AF35 UHM domain also interacts with the U2AF2 RRM1 domain [39]. The RNAH motif lies between U2AF2, ULM, and RRM1, raising the question of whether it affects the interaction between U2AF2 and U2AF1. Our pull-down experiments on *C. elegans* or human U2AF heterodimers, together with AlphaFold modeling of the heterodimers, did not detect obvious disruption of their interactions. In addition, the disease-causing hU2AF2 M144I mutation, which lies in the middle of RNAH, did not obviously affect hU2AF2 interaction with hU2AF1 [39]. Nevertheless, detailed experiments will be required to rigorously test whether RNAH influences U2AF2 interactions with U2AF1 and potentially other proteins.

Still, important questions remain. For example, what mechanisms underlie the coordinated binding to the branch point, the PPT, and the splice acceptor? How is the precise selection of alternative 3’ss achieved? And how do additional *cis* and *trans* factors affect the process? A question specific to *C. elegans* is whether the two UAF-1 RRM motifs bind the PPTs. Because the structures in this study were primarily predicted by AlphaFold and MDs, future studies using X-ray crystallography, NMR, precise binding assays, *in vivo* experiments, and further *in silico* modeling are warranted to test this model.

## Conclusions

Many human diseases, e.g. cancers and neurodevelopmental disorders, are caused by defects in RNA splicing [6]. Alternative splicing is also involved in autism spectrum disorders and aging [113–116]. Small molecules targeting U2AF2 or other splicing factors have been actively pursued as potential treatments of splicing-related cancers [41, 117, 118]. Our findings provide new mechanistic insights into how U2AF2 modulates genome-wide alternative splicing.

## Funding

National Natural Science Foundation of China (Grant/Award Number: 31972877). Funding to pay the Open Access publication charges for this article was provided by the National Natural Science Foundation of China.

## Supplementary Material

gkaf1347_Supplemental_Files

## Data Availability

RNA-Seq datasets generated and analyzed in this study are available in the SRA (https://www.ncbi.nlm.nih.gov/sra) with the accession numbers PRJNA1224051 and PRJNA1224053. AlphaFold-predicted protein structure coordinates, PyMOL scripts, and GROMACS MD simulation files are available in Zenodo (https://doi.org/10.5281/zenodo.17491502). All other data are available from the corresponding author upon reasonable request.
