# Supplementary material for: Genome-wide modulation of alternative splicing by a predicted alpha helix in U2AF2
Source: Nucleic Acids Res. 2025 Dec 17;53(22):gkaf1347. doi: 10.1093/nar/gkaf1347 (PMC12709185; doi:10.1093/nar/gkaf1347)
Supplement: gkaf1347_Supplemental_Files [file gkaf1347_supplemental_files.zip › Raw Data_AlphaFold Predictions.docx]

**Raw Data_** **AlphaFold Predictions**


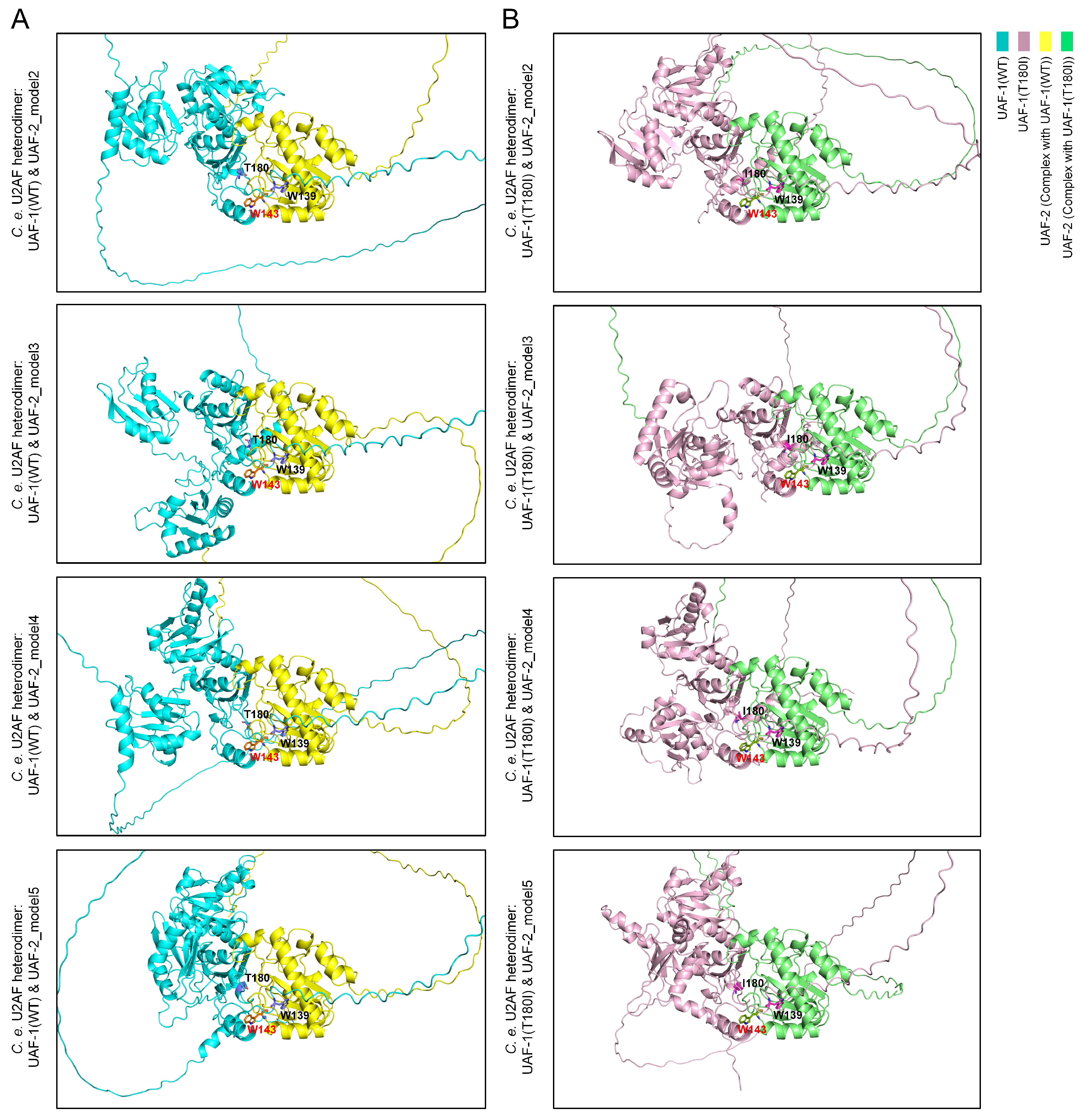
Related to Figures S9 and S10

**Raw Data_** **AlphaFold Predictions Figure 1**

(A) *C. elegans* UAF-1(WT)/UAF-2(WT) heterodimers (models 2 to 5).

(B) *C. elegans* UAF-1(T180I)/UAF-2(WT) heterodimers (models 2 to 5).


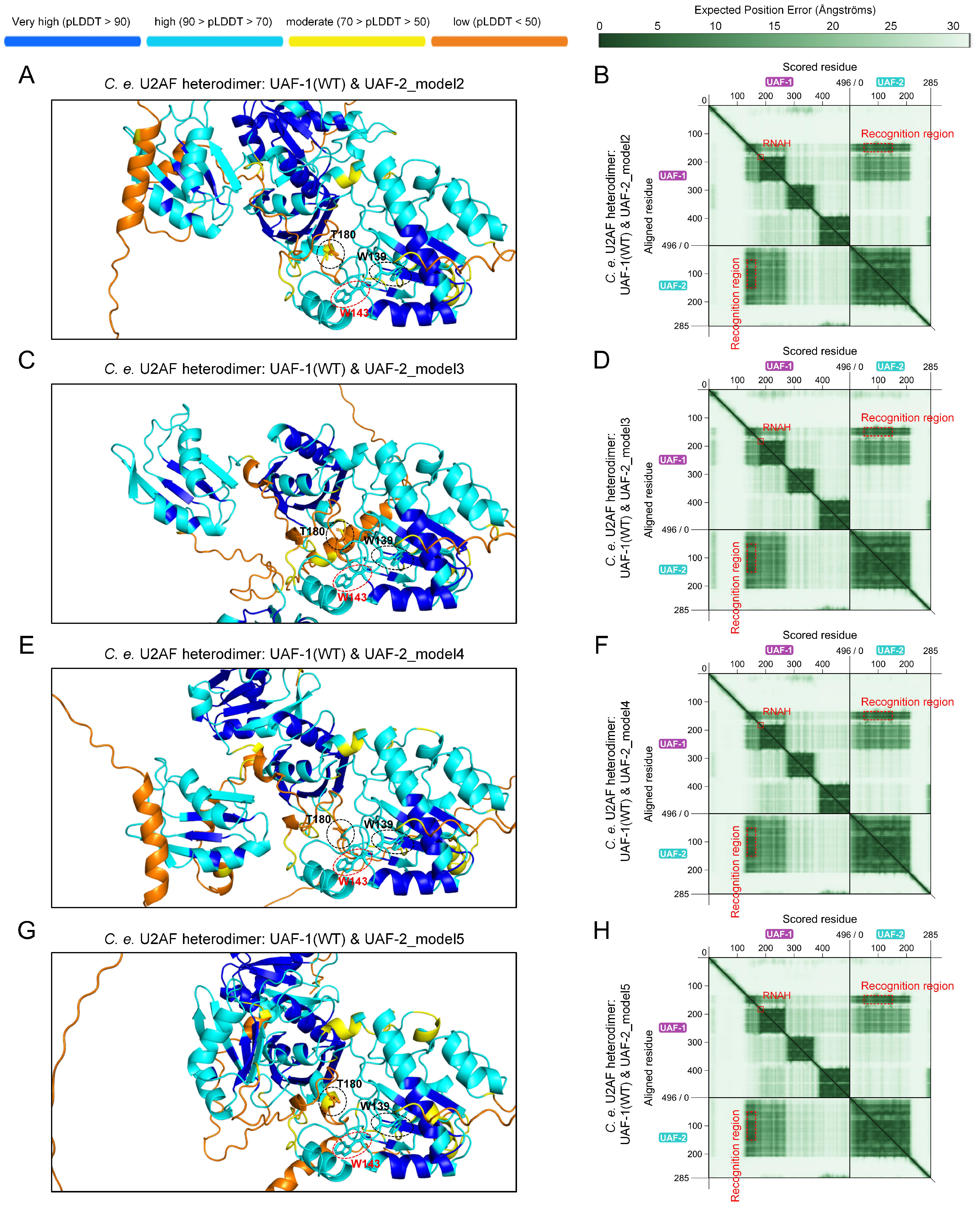
**R****aw Data_** **AlphaFold Predictions Figure 2**

(A, C, E, G) *C. elegans* UAF-1(WT)/UAF-2(WT) heterodimers (models 2 to 5) color coded showing the pLDDT scores in various regions of the predicted structures.

(B, D, F, H) Corresponding PAE plots of the predicted structures. The RNAH motif and the regions of reciprocal tryptophan recognitions between UAF-1 and UAF-2 are enclosed and indicated.


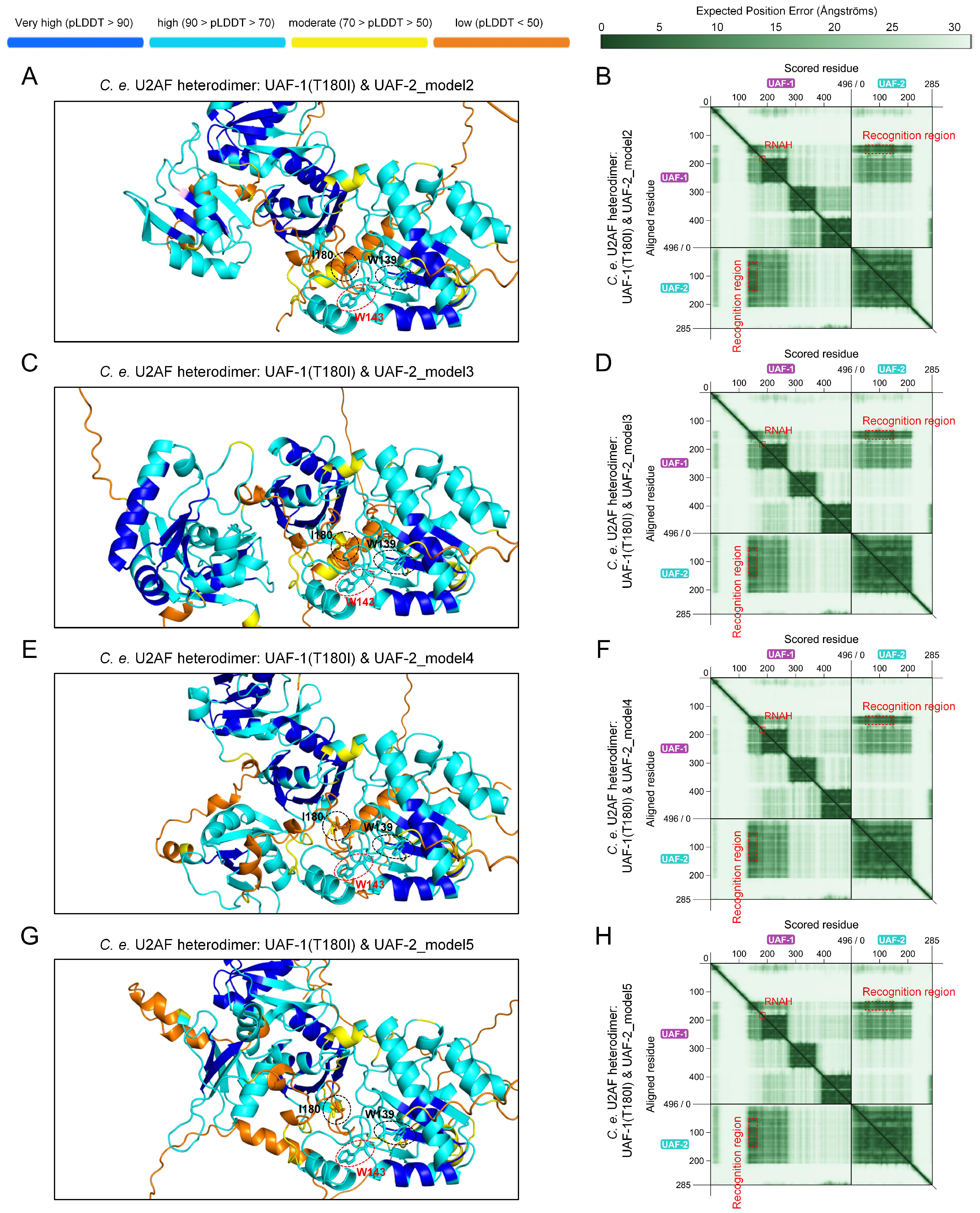
**Raw Data_** **AlphaFold Predictions Figure 3**

(A, C, E, G) *C. elegans* UAF-1(T180I)/UAF-2(WT) heterodimers (models 2 to 5) color coded showing the pLDDT scores in various regions of the predicted structures.

(B, D, F, H) Corresponding PAE plots of the predicted structures. The RNAH motif and the regions of reciprocal tryptophan recognitions between UAF-1 and UAF-2 are enclosed and indicated.


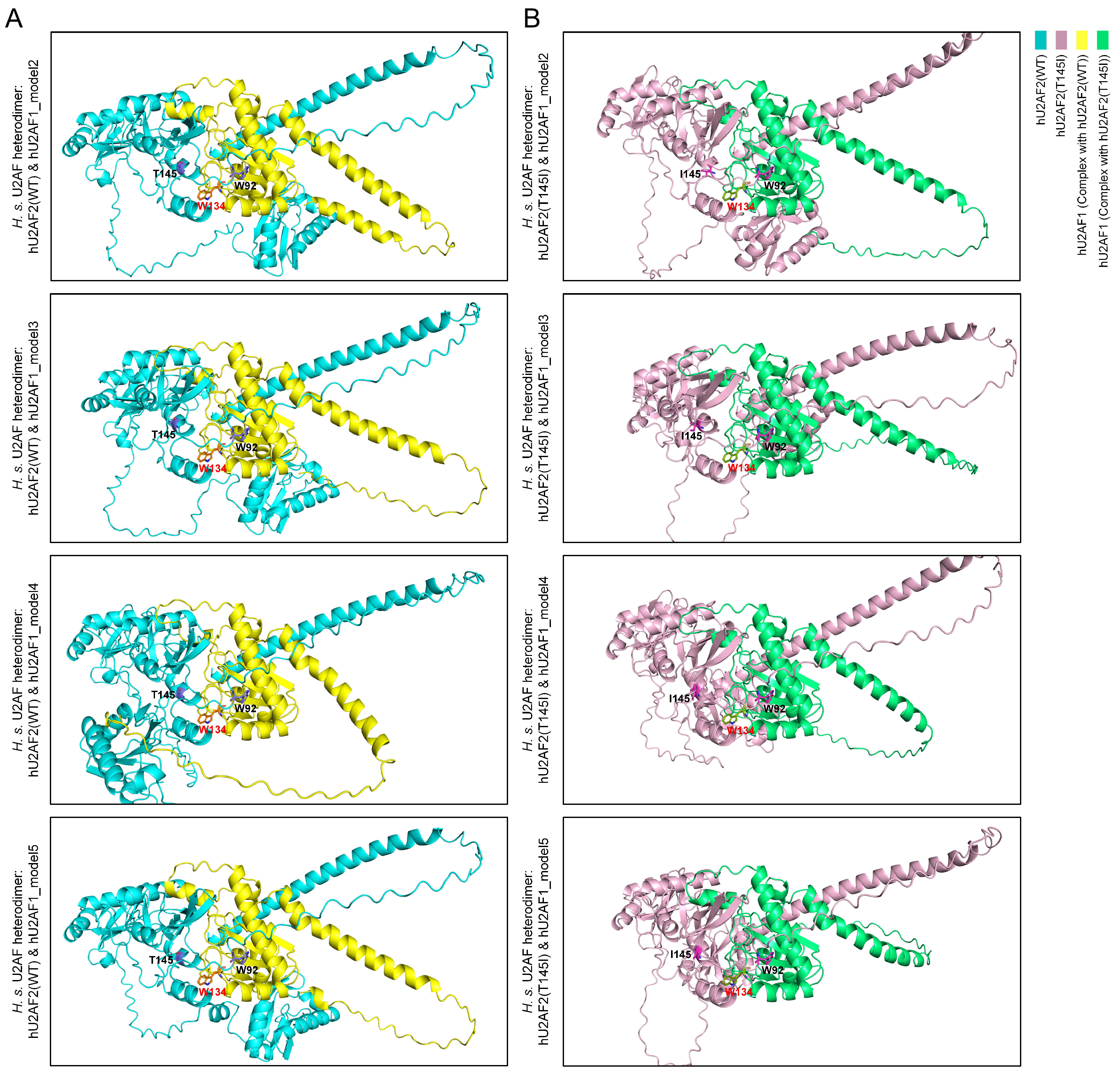
**Raw Data_** **AlphaFold Predictions Figure 4**

(A) Human U2AF2(WT)/U2AF1(WT) heterodimers (models 2 to 5).

(B) Human U2AF2(T145I)/U2AF1(WT) heterodimers (models 2 to 5).


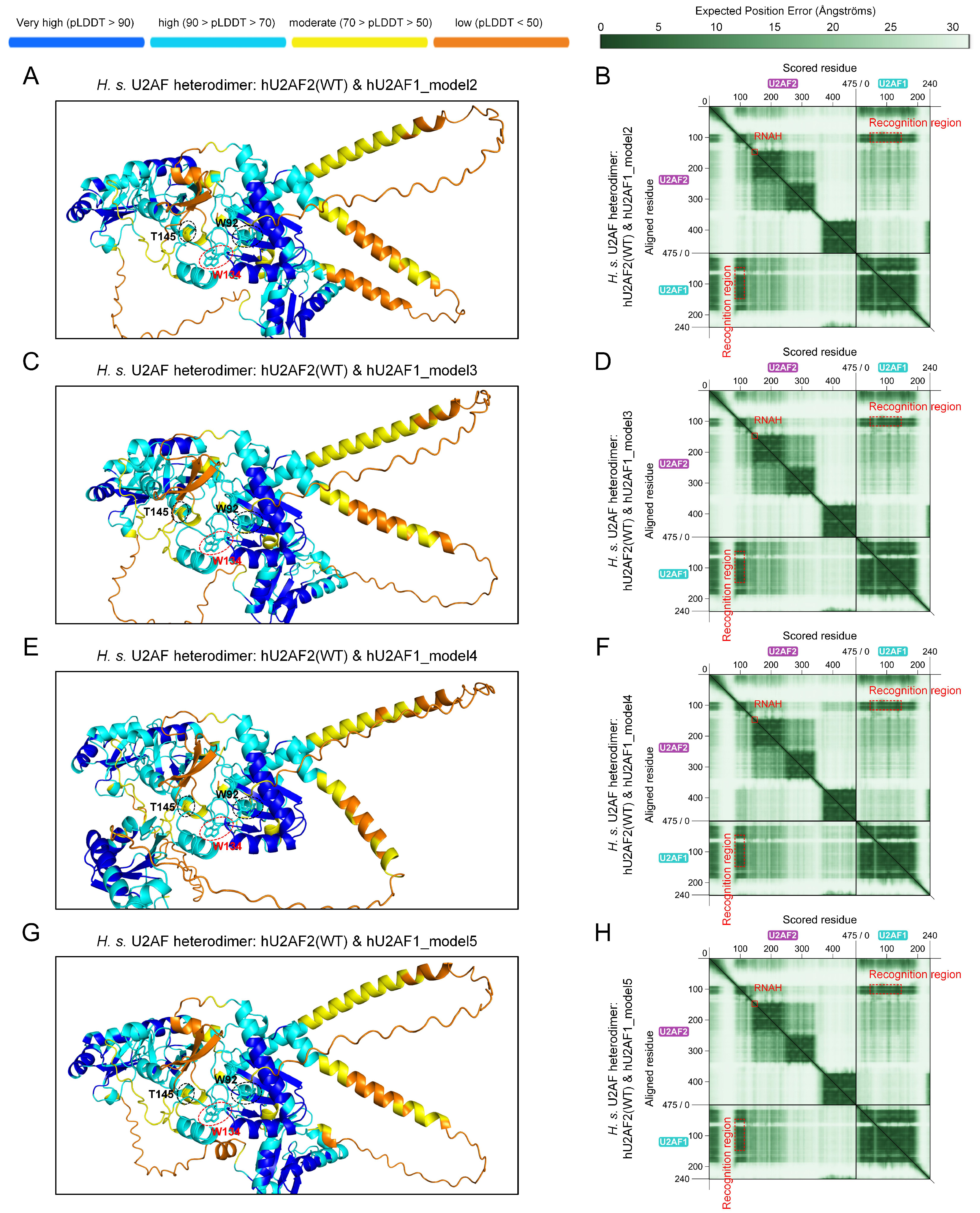
**Raw Data_** **AlphaFold Predictions Figure 5**

(A, C, E, G) Human U2AF2(WT)/U2AF1(WT) heterodimers (models 2 to 5) color coded showing the pLDDT scores in various regions of the predicted structures.

(B, D, F, H) Corresponding PAE plots of the predicted structures. The RNAH motif and the regions of reciprocal tryptophan recognitions between U2AF2 and U2AF1 are enclosed and indicated.


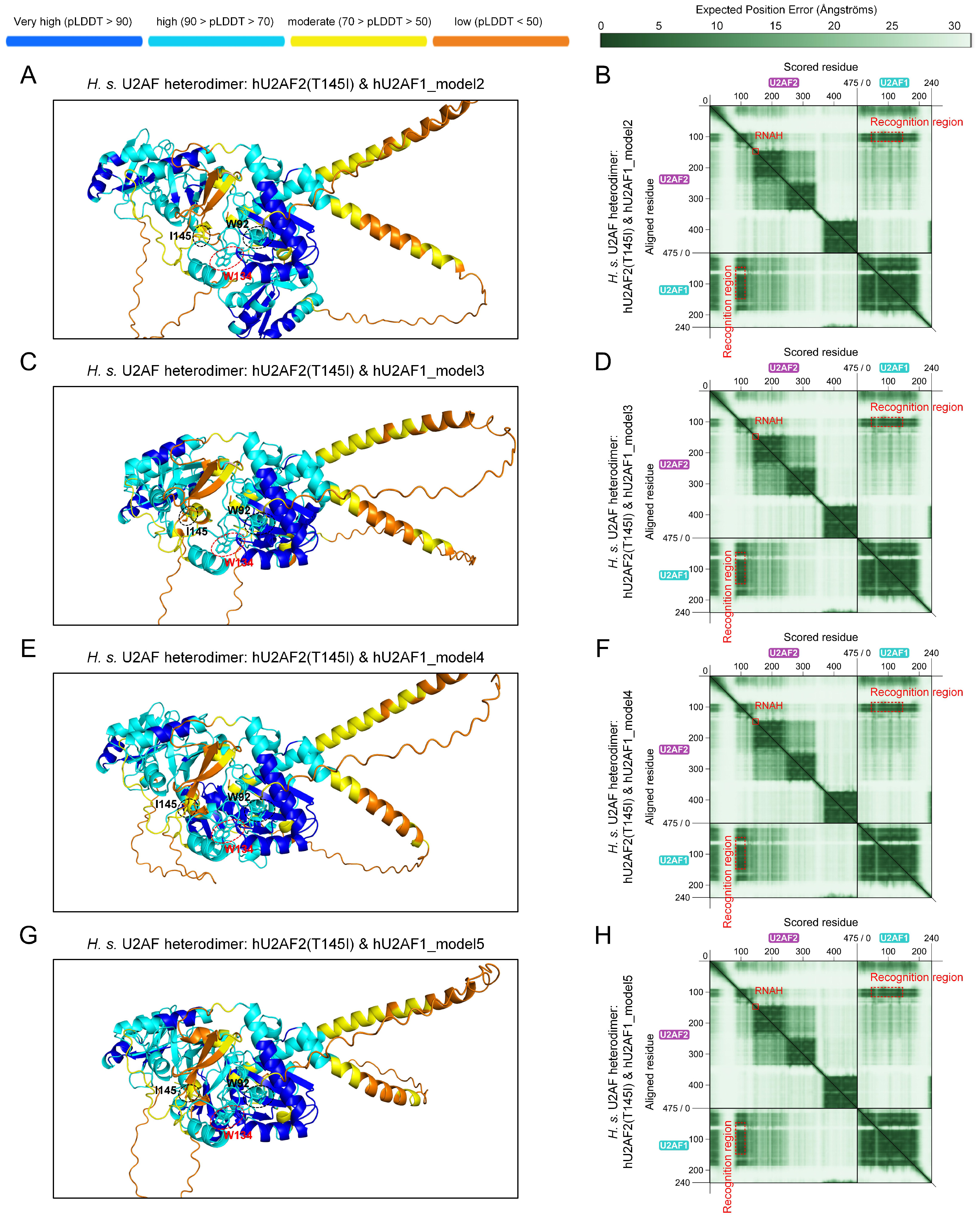
**Raw Data_** **AlphaFold Predictions Figure 6**

(A, C, E, G) Human U2AF2(T145I)/U2AF1(WT) heterodimers (models 2 to 5) color coded showing the pLDDT scores in different regions of the predicted structures.

(B, D, F, H) Corresponding PAE plots of the predicted structures. The RNAH motif and the regions of reciprocal tryptophan recognitions between U2AF2 and U2AF1 are enclosed and indicated.
