# Supplementary material for: Genome-wide modulation of alternative splicing by a predicted alpha helix in U2AF2
Source: Nucleic Acids Res. 2025 Dec 17;53(22):gkaf1347. doi: 10.1093/nar/gkaf1347 (PMC12709185; doi:10.1093/nar/gkaf1347)
Supplement: gkaf1347_Supplemental_Files [file gkaf1347_supplemental_files.zip › Raw Data_EMSA.docx]

**Raw Data_EMSA**

Related to Figure S22


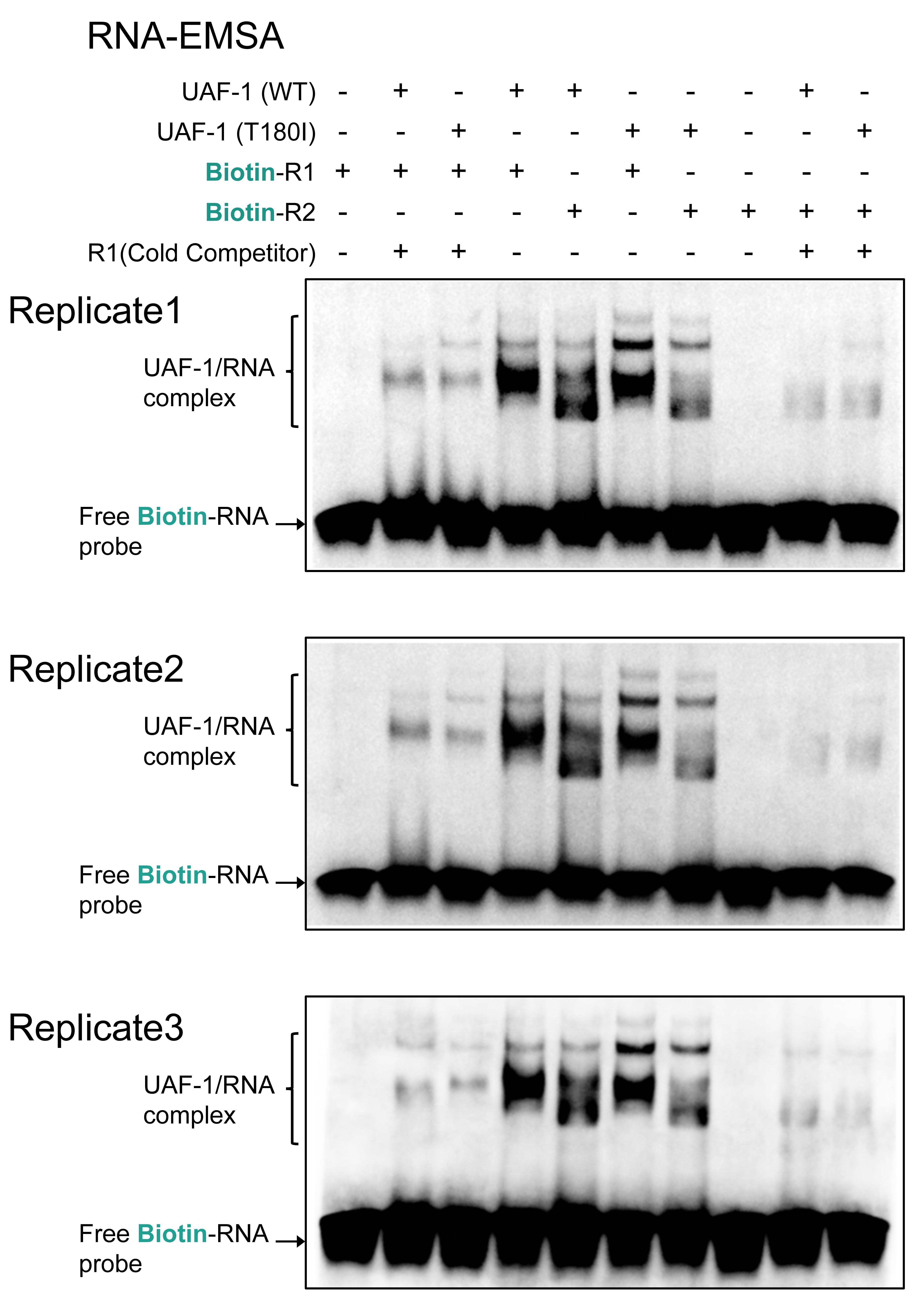

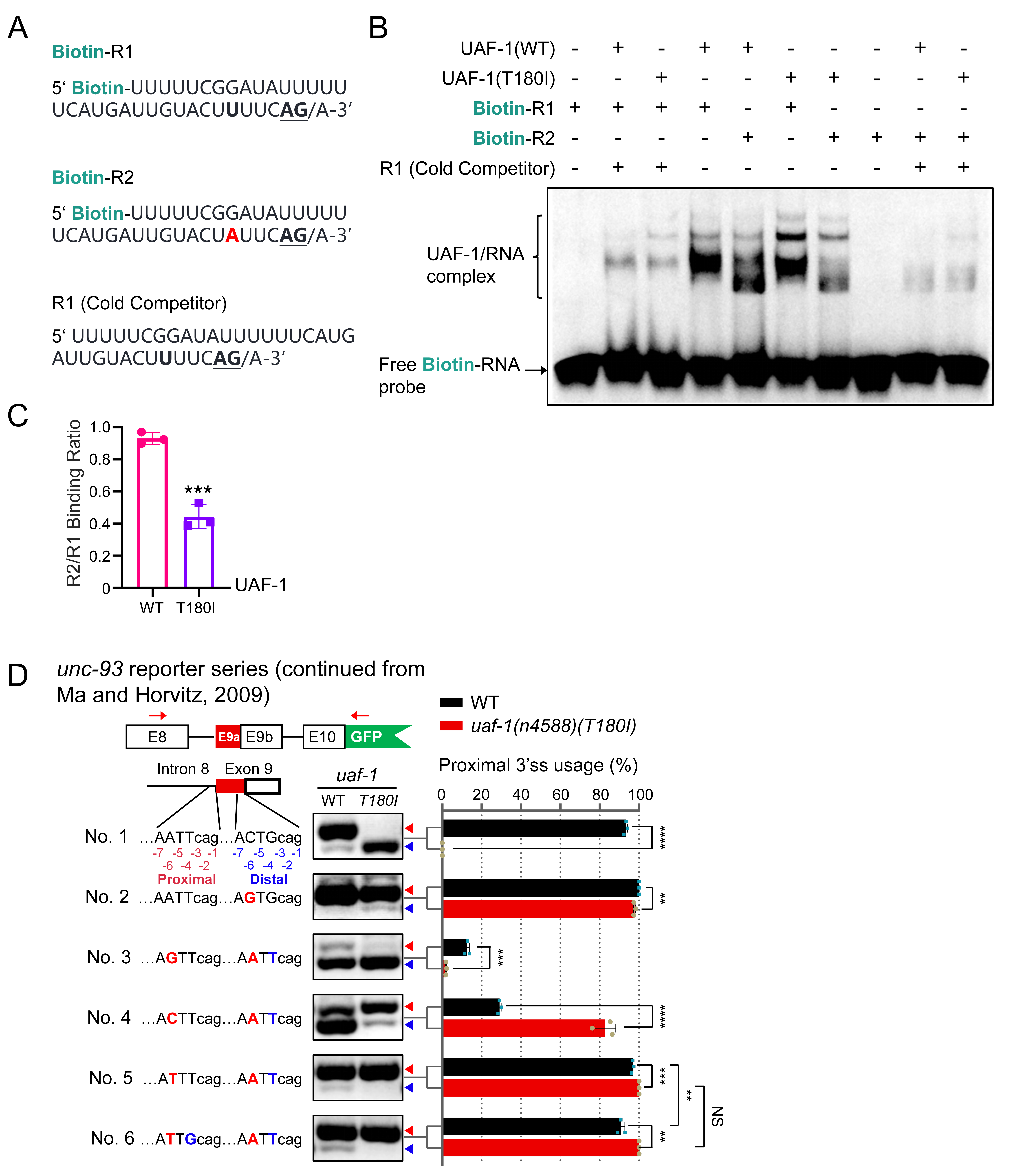

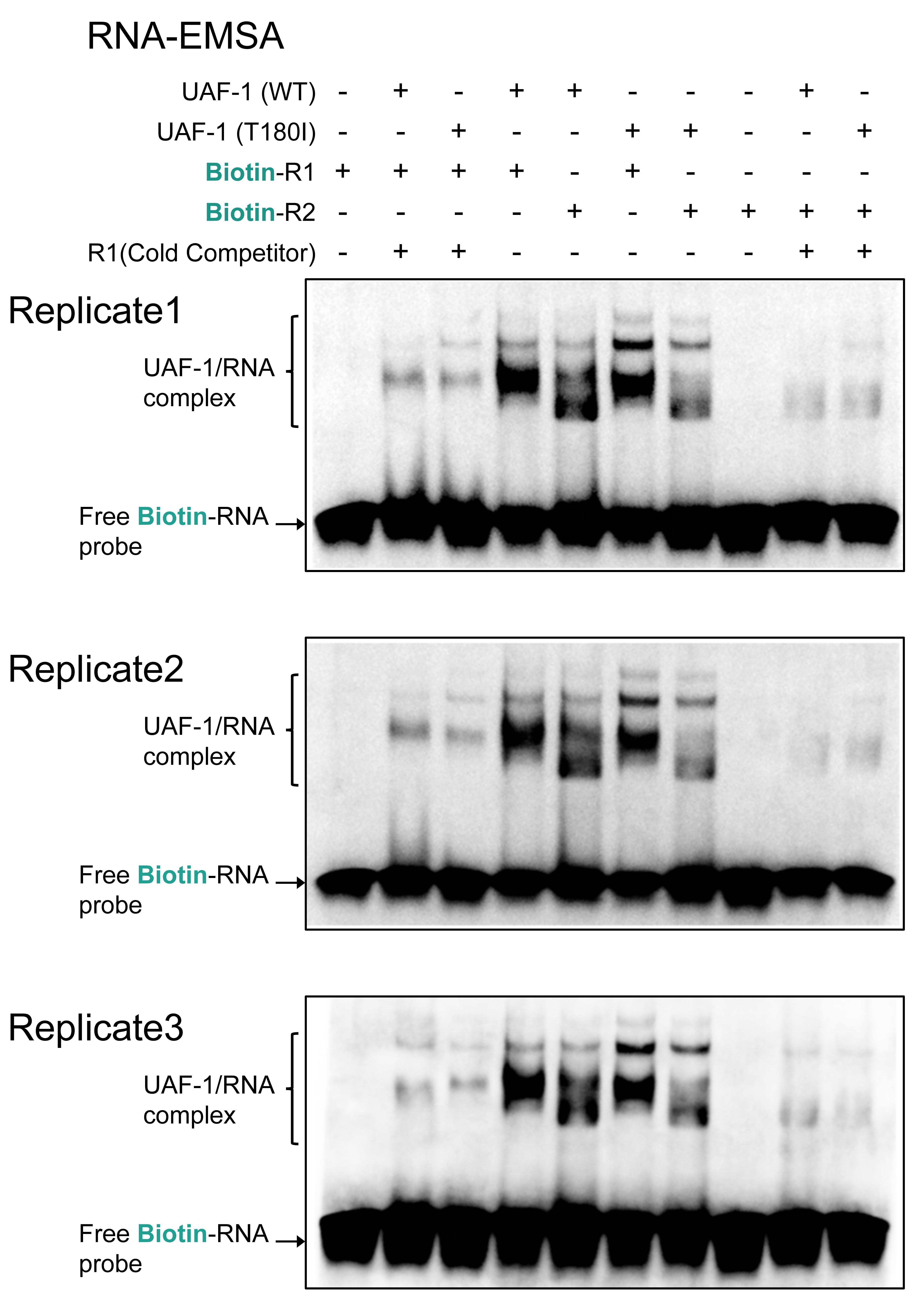


**Raw Data _EMSA Figure 1**

Electrophoretic mobility shift assay (EMSA), shown as representative gels from three independent replicates, depicting UAF-1 binding to RNA probes with or without a cold competitor. Biotin-labeled 3’ss RNAs (Biotin-R1 and Biotin-R2) or unlabeled R1 (cold competitor) were incubated with purified UAF-1(WT) or UAF-1(T180I) as indicated above the lanes. UAF-1/RNA complexes are marked by shifted bands, and free Biotin-RNA probes appear at the bottom. UAF-1(WT) binds R1 and R2, whereas the T180I mutant shows reduced interaction with R2.
