# Supplementary material for: Genome-wide modulation of alternative splicing by a predicted alpha helix in U2AF2
Source: Nucleic Acids Res. 2025 Dec 17;53(22):gkaf1347. doi: 10.1093/nar/gkaf1347 (PMC12709185; doi:10.1093/nar/gkaf1347)
Supplement: gkaf1347_Supplemental_Files [file gkaf1347_supplemental_files.zip › Raw Data_Reporter Sequences.docx]

**Raw Data_Reporter Sequences**

Related to Figures 5 and 6 and Supplementary Figure S22

*hNFRKB* minigene reporter full sequence

......tggtagccatttatctccccagcaaagaaaccctcttgagttgtctggtctggggtgcatagcgggactcagaaggccttggtctttgggttgctgagacattgccatcagatggggagtgactccatggcctttttcacccttgtggccatgtggcatttgagcttagatgagagcccgccagacgactccctgcacgacggggttctccacagtgggcctgactggtcccatttgccacttgagagaactgttttccccttacaggagagtagtagtggctcttggcctgctgcaatgcaaccccagcactctgttgaagtctctcttcctggaagagccgttagatgacctaagtgtgctccagctcctctggggtagggtcagaaaggattgtggaaccctggcctccatgttgttggcttagccttgttgaacccaaatggccacttacccaatctcccctattggagtccac**ag**CCACGTTGGATTTACA**AG**AACAATTTTCTTTTGAAGATCTCAGCTCATGGCTTCCGAGCTCTCCAGCACGTTCTCCTAGTCCTGCGGTGCCCCTGCGGGTGGTGCCCACACTTTCAACCACGGATATGAAAACTGCAGgtgagaaccaagctgccgctgtcaccattctgggacattgcggtggagacctgaaaactcctttagagggaggtgggtgggggcagattcctacctaccaggaaaacaaaactccctagaactaggtgatttgtttggcaaagtttttgtaccattttacttttttttacatctggagataggcctgcctt......

*unc-93* minigene reporter full sequence

......ATACTCTTCTATGATCCCTGCTTCAATATTCTGTGGAATCGCGGCTTCATGTATCTGGGGAGCCAAATGCGCGTACATTACAGAAATGGGAATTCGATATGCTAGCCTGAACTTTGAAAGTCAGACTACTGTTATTGTTAGgtaatgggctgattttctaaacctttttcaagcttttcaagcttttaatcaagccaaattc**ag**ATTTTTCGGATATTTTTTCATGATTGTACACTGC**AG**ACAAGTCGTCGGAAATATGGTATCCTCTTATATTTTTACACTGTCCTATTCGCAAGCCCTACGCGGTCCTGAAGATTCTATATACGATAGgtttgccgaaatttttttttgaagttttaaaatttcaattttccagCTGTGGCTACCAGTTTCCAAAGAATTTATCAGATCTTACCGAGTTGGCGGAGAGCAATCTTGCTCGGCCACCACAGAAAGTTTATGTAGC......

*attf-5* minigene reporter full sequence

......CACACCACCACCAGTCAAAAAAACTCGAGGACGGCCTCGCAAAGATCGATCACAAGAGCCAACGGCAGTACCTAAGCATATGCAGAGAATGCGGAAACCGAAAAgtaagtgcttgagagtgttgtgccacctgcatgttgctaaatatgttttcctctc**ag**CTCTTGTACACGACGGTGCACTGATTCCCGTTGC**AG**AAACTAAACTGAACCCGGAAAATGACGAGAAAGCAGAGGAAGAGAGAAAAGCATTACTGCAAGCGCTGCCAACAAATCTGCTAAATGCAATCCAAGCGCAAAAAGCTATGGATAGGGAACAAGATAAG......

Note: Alternative 3′ splice acceptor positions are marked in bold and underlined; nucleotides contributing to the extended (long) exon but absent from the shorter exon are highlighted in red.
