# Supplementary material for: Genome-wide modulation of alternative splicing by a predicted alpha helix in U2AF2
Source: Nucleic Acids Res. 2025 Dec 17;53(22):gkaf1347. doi: 10.1093/nar/gkaf1347 (PMC12709185; doi:10.1093/nar/gkaf1347)
Supplement: gkaf1347_Supplemental_Files [file gkaf1347_supplemental_files.zip › UAF-1-NAR-Sup_R2.pdf]

Supplementary Material

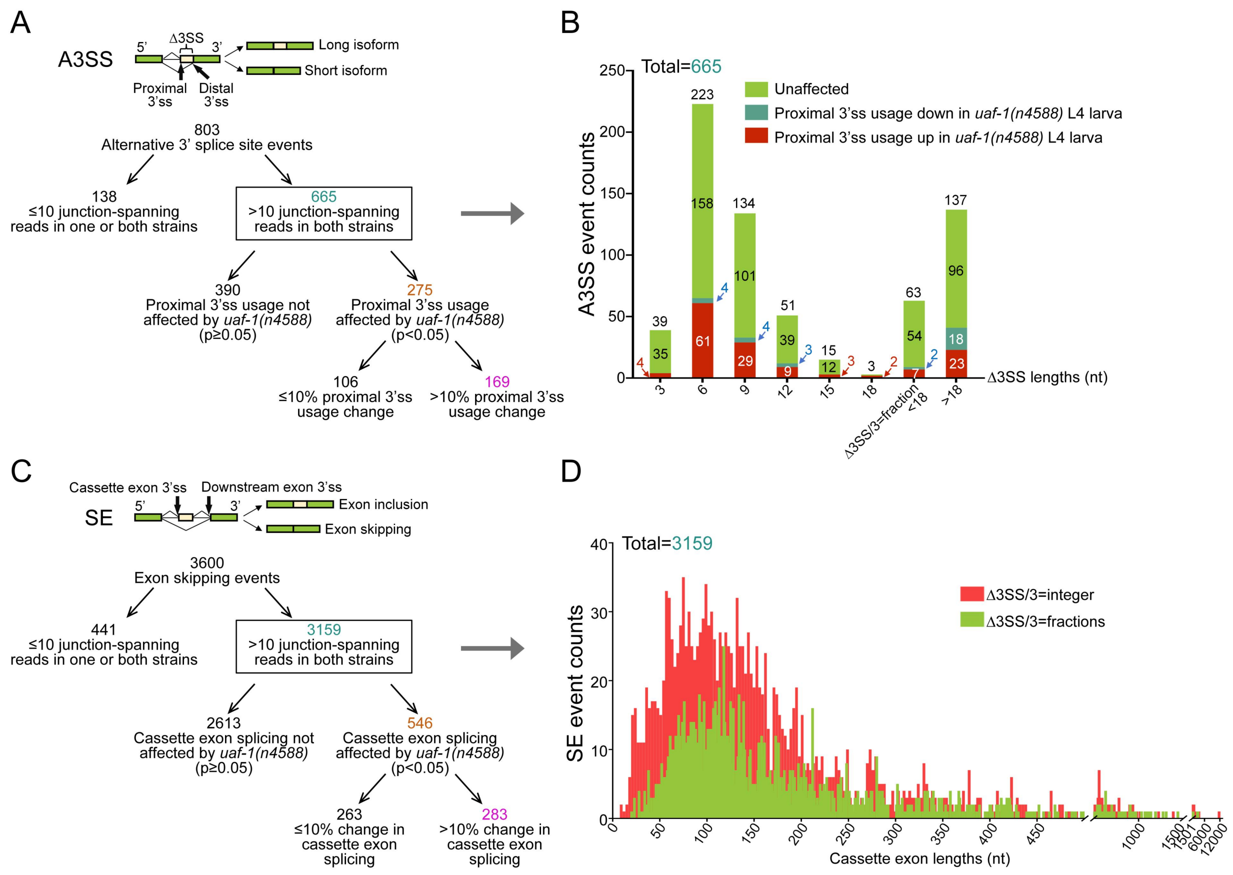

Figure S1. *uaf-1(n4588)* significantly alters A3SS and SE events at the L4 larval stage.

(A) A diagram showing the generation of A3SS events and the triaging process based on a previously described method (1).

(B) The distribution of various types of A3SS events with more than (>) 10 RNA-Seq reads on both strands. *uaf-1(n4588)* affected a fraction of each type. The total number of events is indicated on top. Events not affected are marked in green, whereas events with decreased or increased usage of the proximal 3'ss are marked in blue or red.

(C) A diagram showing the generation of SE events and the triaging process.

(D) The distribution of SE events based on cassette exon lengths.

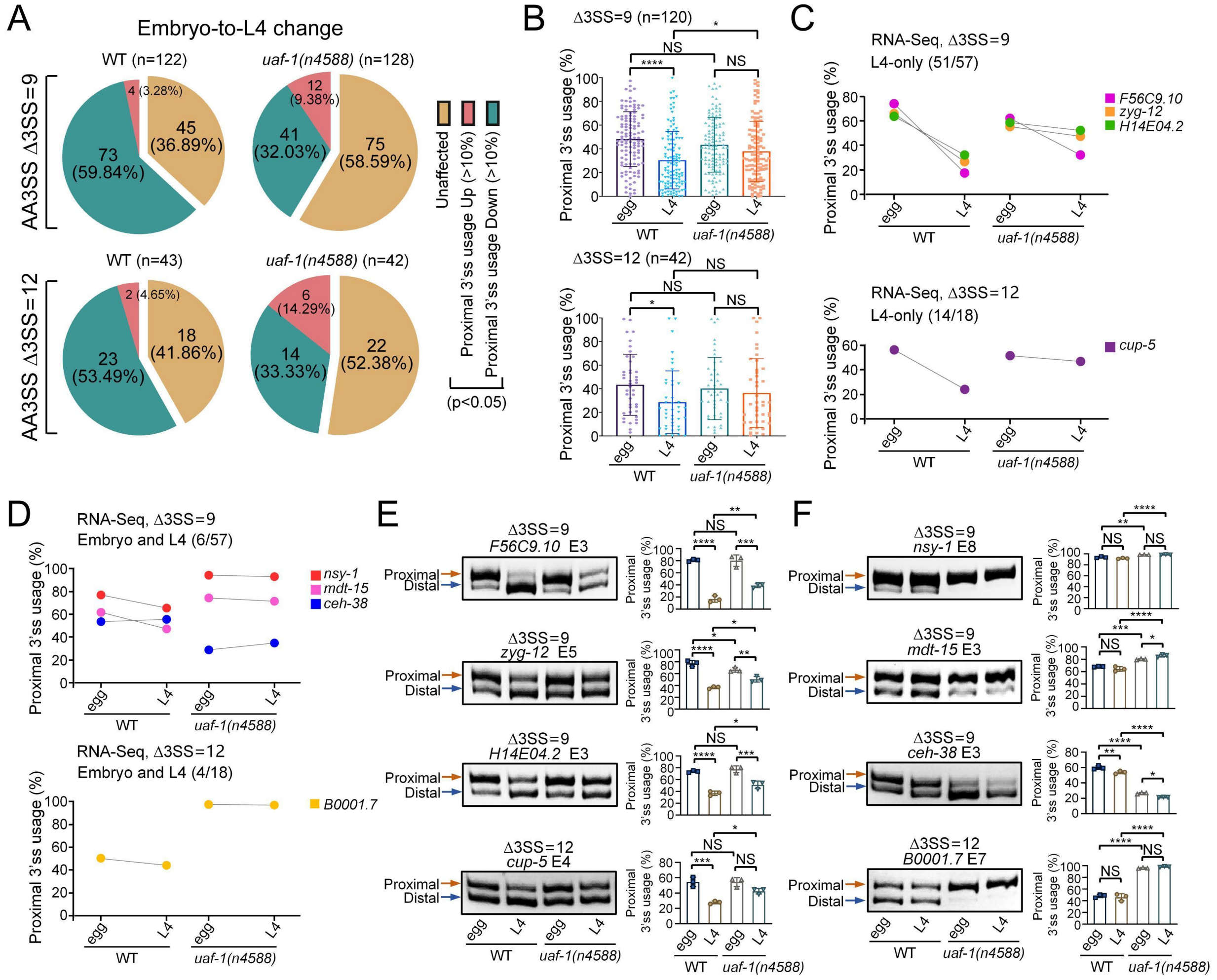

Figure S2. *uaf-1(n4588)* disrupts development-dependent usage of proximal 3'ss in AA3SS events (Δ3SS=9 or 12).

(A) Pie charts showing the number of AA3SS events with or without embryo-to-L4 changes in proximal 3'ss usage in wildtype or *uaf-1(n4588)* animals.

(B) Scatter plot showing percent proximal 3'ss usage in AA3SS events at the embryonic and L4 larval stages. Statistics: Tukey's multiple comparison test with one-way ANOVA. *: $p < 0.05$; ****: $p < 0.0001$. NS: not significant.

(C) RNA-Seq results showing that *uaf-1(n4588)* mostly affected proximal 3'ss usage in AA3SS events (51/57 events for Δ3SS=9, 14/18 events for Δ3SS=12) only at the L4 stage. Representative events are shown.

(D) RNA-Seq identified a small number of events affected by *uaf-1(n4588)* (6/57 events for Δ3SS=9, 4/18 events for Δ3SS=12) at both embryonic and L4 stages. Representative events are shown.

(E) RT-PCR validation of representative AA3SS events affected by *uaf-1(n4588)* only at the L4 stage. Quantifications were based on three biological replicates. Statistics: Tukey's multiple comparison test with one-way ANOVA. *: p < 0.05; **: p < 0.01; ***: p < 0.001; ****: p < 0.0001. NS: not significant.

(F) RT-PCR validation of representative AA3SS events affected by *uaf-1(n4588)* at both embryonic and L4 stages. Quantifications were based on three biological replicates. Statistics: Tukey's multiple comparison test with one-way ANOVA. *: p < 0.05; **: p < 0.01; ***: p < 0.001; ****: p < 0.0001. NS: not significant.

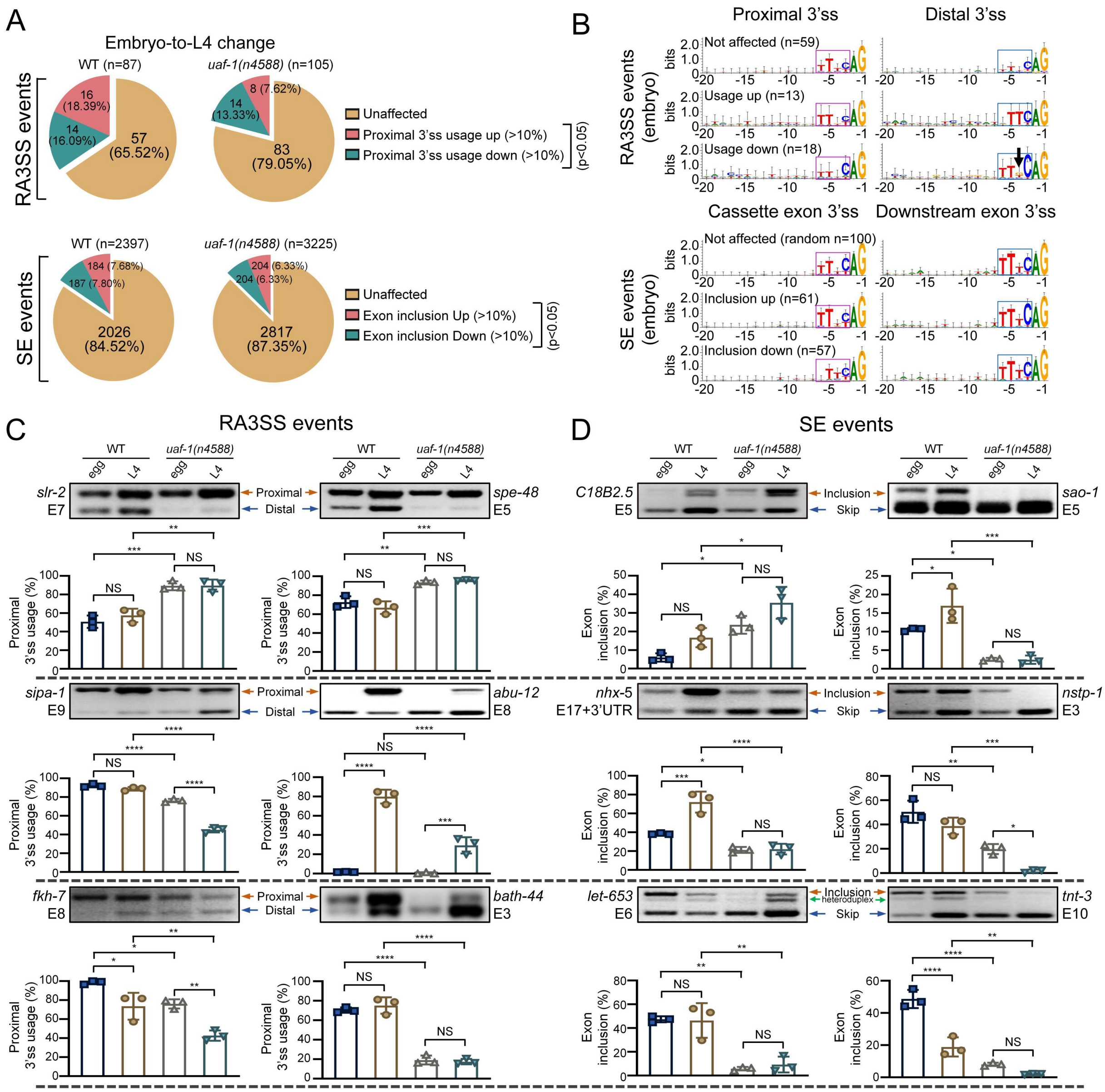

Figure S3. Similar PPT sequences are associated with RA3SS and SE events affected by *uaf-1(n4588)* at the embryonic and L4 stages.

(A) Pie charts showing the numbers of RA3SS and SE events with embryo-to-L4 changes in proximal 3'ss usage or cassette exon inclusion in wildtype or *uaf-1(n4588)* animals.

(B) WebLogo comparisons of proximal and distal 3'ss sequences in RA3SS events, and the preceding 3'ss sequences of the cassette exons (skipping exons) and the downstream exons in SE events in embryos. Nucleotides at positions -3 to -6 of 3'ss are outlined in red and blue, respectively.

(C, D) RT-PCR validation of representative RA3SS (C) and SE (D) events. PCR products were separated on a 2.5% agarose gel. Quantifications were based on three biological replicates. Statistics: Tukey's multiple comparison test with one-way ANOVA. *: $p < 0.05$; **: $p < 0.01$; ***: $p < 0.001$; ****: $p < 0.0001$. NS: not significant.

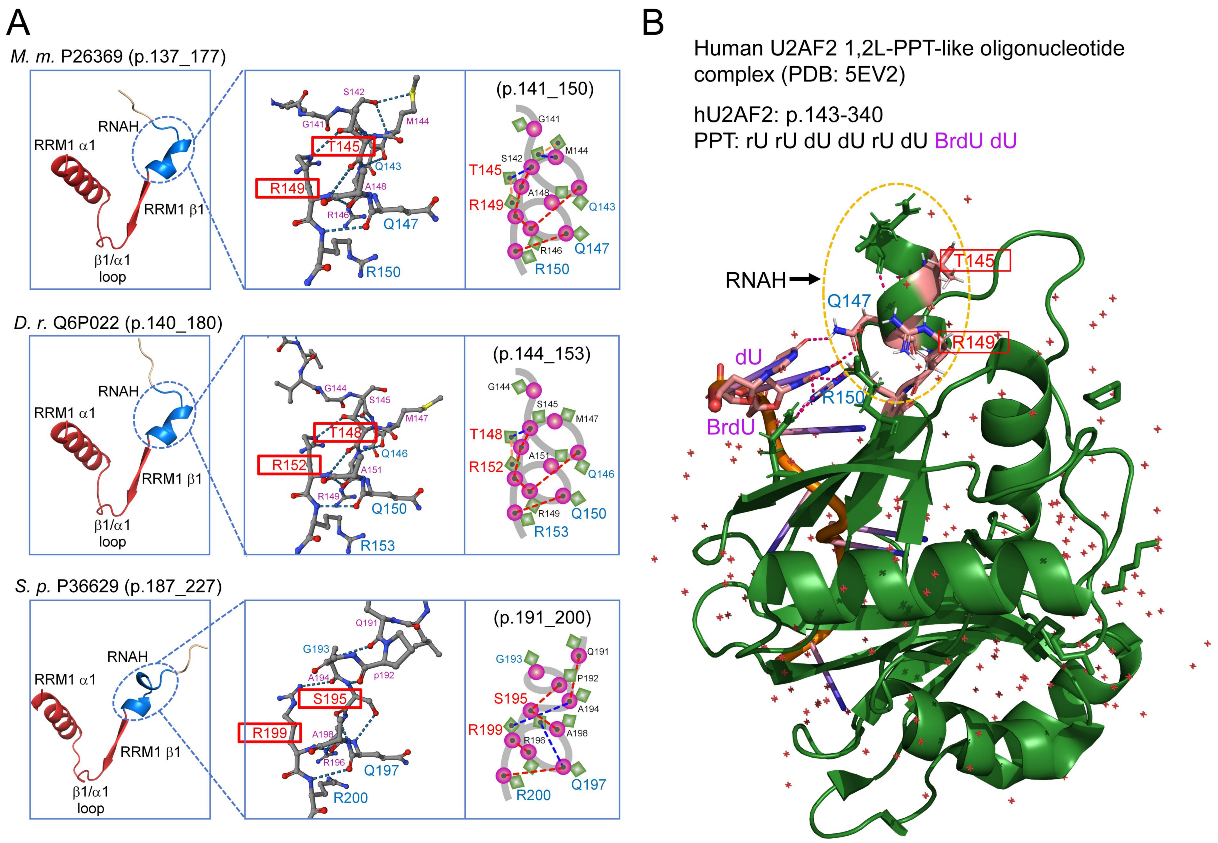

Figure S4. U2AF2 RNAH is predicted to be a conserved structural motif.

(A) Residue positions and H bonds in AlphaFold-predicted RNAH motifs. *M. m.*: *Mus musculus*. *D. r.*: *Danio rerio*. *S. p.*: *Schizosaccharomyces pombe*. Left subpanels: ribbon diagrams of RNAHs (encircled) are shown as the N-terminal extension of RRM1s (red). Segments of RRM1 were labeled based on a solution structure (2). Middle subpanels: RNAH structures, visualized with stick line sidechains, showing H bond formation (dashed lines). Orthologous residues of UAF-1 T180 and R184 in other U2AF2 structures are outlined. Right subpanels: cartoon diagrams of RNAH motifs. Main chains are represented with purple spheres and sidechains with green diamonds. H bonds are indicated with dashed lines (red: main chain bonds; blue: bonds between the main chains and sidechains; yellow: bonds between the sidechains).

(B) The crystal structure of a hU2AF2 (p.143-340) protein (green) in complex with a PPT-like oligonucleotide (orange) (PDB code: 5EV2) (3), showing H bonds formed between 3' nucleotides of the oligonucleotide with the sidechains of Q147 and R150.

The sidechains of T145 and R149 (outlined) are orientated away from the nucleotides. “*” represents H_2O.

A

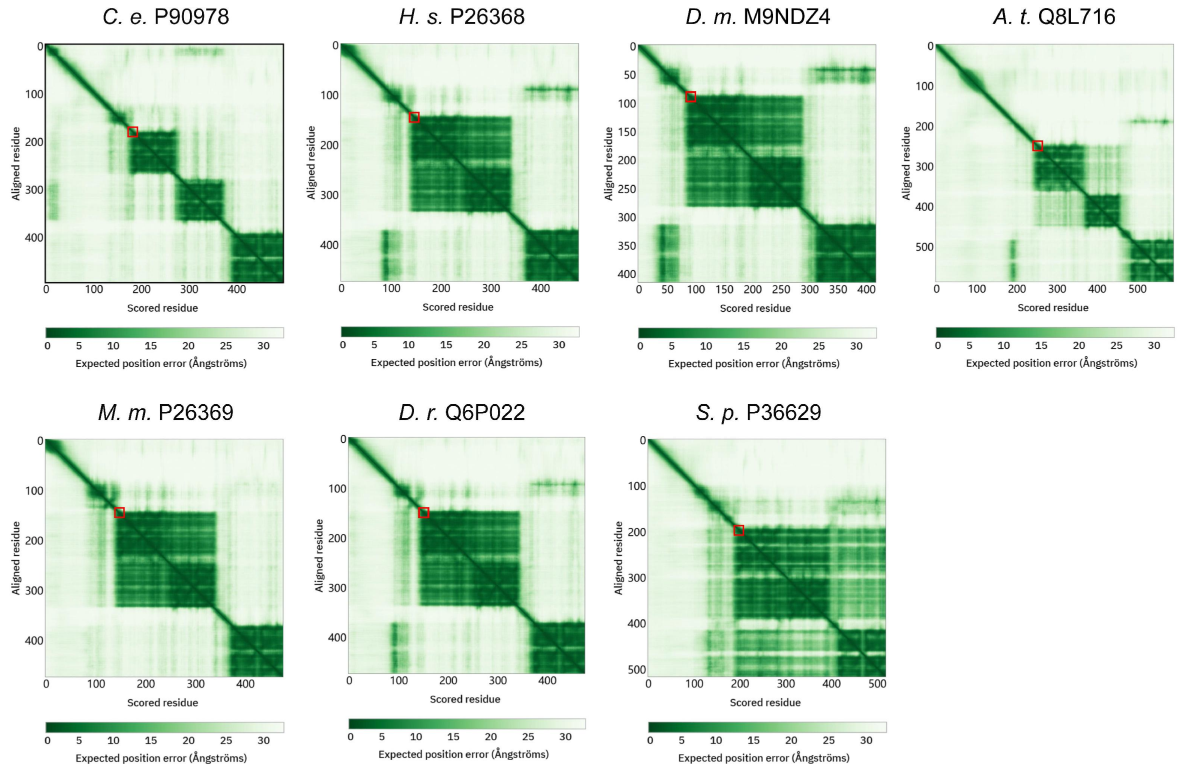

B

	C. e. P90978		*H. s.* P26368		*D. m.* M9NDZ4		*A. t.* Q8L716	
	Residue	pLDDT	Residue	pLDDT	Residue	pLDDT	Residue	pLDDT
RNAH Motif	G176	33.44	G141	48.15	G85	39.03	T247	52.52
	P177	40.40	S142	57.14	S86	44.16	Q248	60.21
	S178	37.83	Q143	62.55	T87	48.59	Q249	63.31
	V179	45.85	M144	61.61	I88	48.34	A250	63.16
	T180	60.74	T145	74.46	T89	67.12	T251	77.44
	C181	60.48	R146	80.68	R90	73.81	R252	80.09
	Q182	70.34	Q147	84.08	Q91	79.38	H253	83.67
	S183	80.14	A148	86.94	A92	83.12	A254	86.75
	R184	87.82	R149	90.88	R93	89.56	R255	90.30
	M. m. P26369		*D. r.* Q6P022		*S. p.* P36629			
	Residue	pLDDT	Residue	pLDDT	Residue	pLDDT		
RNAH Motif	G141	49.48	G144	46.72	Q191	57.34		
	S142	54.44	S145	45.56	P192	58.88		
	Q143	62.54	Q146	45.94	G193	59.70		
	M144	60.70	M147	43.62	A194	61.81		
	T145	75.79	T148	66.38	S195	70.35		
	R146	81.40	R149	75.12	R196	78.15		
	Q147	84.49	Q150	81.06	Q197	79.85		
	A148	87.59	A151	84.31	A198	83.98		
	R149	91.39	R152	89.88	R199	89.69		

Figure S5. PAE plots for the predicted U2AF2 structures and per-residue pLDDT scores for the RNAH motif.

(A) Predicted Aligned Error (PAE) plots of AlphaFold predicted U2AF2 structures in *C. elegans* (P90978), *H. sapiens* (P26388), *D. melanogaster* (MNDZ4), *A. thaliana*

(Q8L716), *M. musculus* (P26369), *D. rerio* (Q6P022), and *S. pombe* (P36629). The red squares indicate the range of the RNAH motif.

(B) pLDDT (Predicted Local Distance Difference Test) score of each RNAH residue, providing an assessment of the reliability of structural predictions. Residues with pLDDT scores below 50 are shown in gray, and the conserved *C. elegans* RNAH residues T180, Q182 and R184 and their orthologs are highlighted in red.

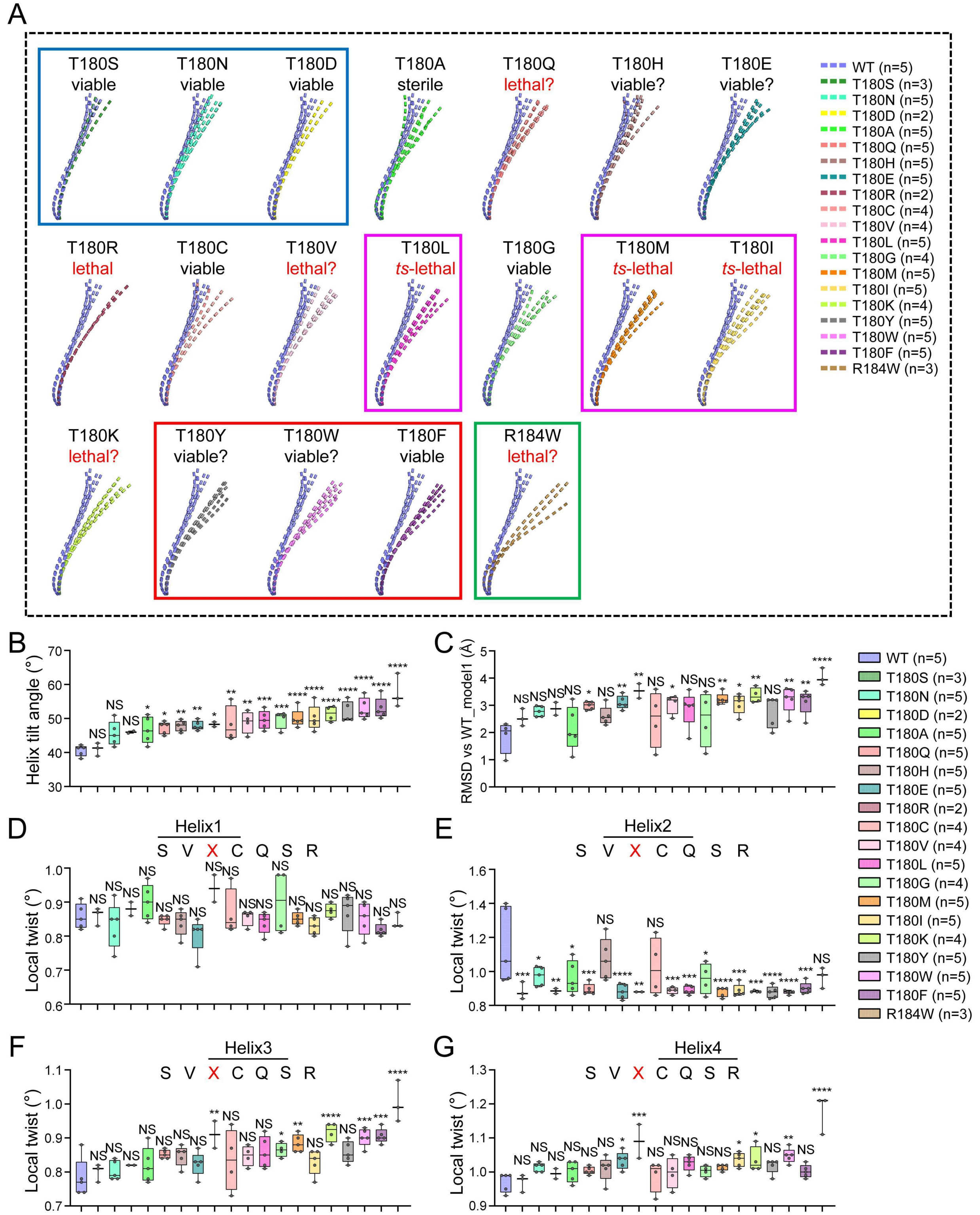

Figure S6. *In silico* UAF-1 RNAH conformations carrying different UAF-1 T180X substitutions and the UAF-1 R184W substitution.

(A) The mutations cause the helix main axes to tilt away from that of the wild type at

variable degrees. The axes are arranged in ascending order of tilt angles from left to right. Mutations that exhibit lethal or potentially lethal (marked by “?”) phenotypes are indicated. T180S, T180N and T180D (enclosed in the blue box) had minimal effects, while R184W (enclosed in the green box) caused the largest tilt. T180I-like mutations causing similar helical axes and phenotypes are enclosed in the purple box. Aromatic amino acid mutations (T180F, T180Y and T180W) are enclosed in the red box. Five conformations were predicted by AlphaFold for each mutation. Conformations, including all five carrying the T180P mutation, that completely disrupted the alpha helix structures were not shown. Wildtype conformations were shown in each illustration for comparison purposes.

(B, C) Helical tilt angles (see Fig 2D, right) and RMSDs of the RNAH structures using the wildtype model 1 as reference.

(D, E, F, G) Local twists of each RNAH helical segment.

Results are based on two to five predicted conformations. Statistics: Dunnett's multiple comparison test with one-way ANOVA. *: $p < 0.05$; **: $p < 0.01$; ***: $p < 0.001$; ****: $p < 0.0001$. NS: not significant.

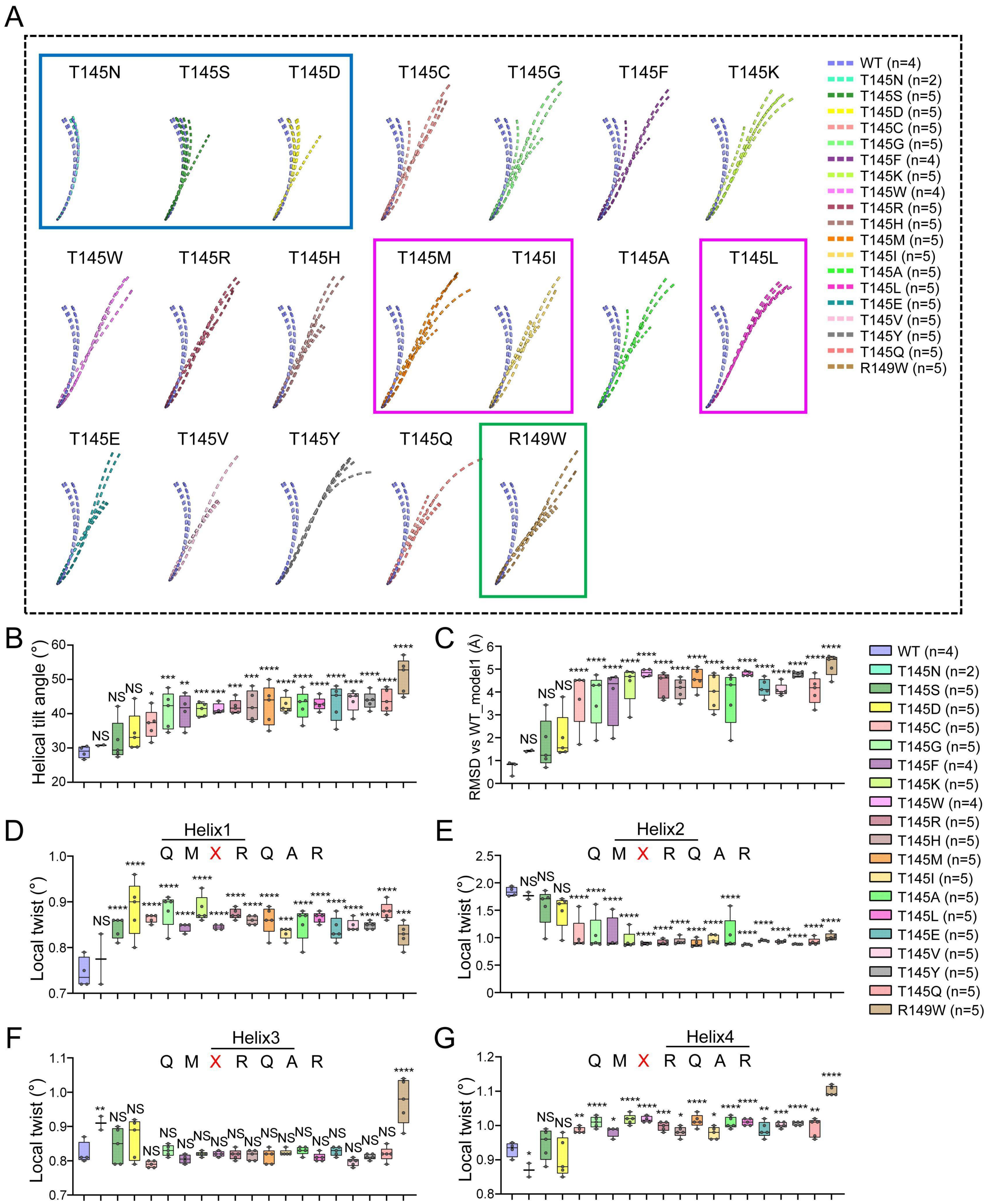

Figure S7. *In silico* hU2AF2 RNAH conformations carrying different hU2AF2 T145X substitutions and the hU2AF2 R149W substitution.

(A) The axes are arranged in ascending order of tilt angles from left to right. T145N, T145S and T145D (enclosed in the blue box) had minimal effects, while R149W

(enclosed in the green box) caused the largest tilt. T145I, T145M, and T145L, orthologous to the UAF-1 T180I-like mutations, are enclosed in the purple box. Five conformations were predicted by AlphaFold for each mutation. Conformations, including all five carrying the T145P mutation, that completely disrupted the alpha helix structures were discarded. Wildtype conformations were shown in each illustration for comparison purposes.

(B, C) Helical tilt angles (see Fig 2H, right) and RMSDs of the RNAH structures using the wildtype model 1 as reference.

(D, E, F, G) Local twists of each helical segment of RNAH.

Results are based on two to five predicted conformations. Statistics: Dunnett's multiple comparison test with one-way ANOVA. *: $p < 0.05$; **: $p < 0.01$; ***: $p < 0.001$; ****: $p < 0.0001$. NS: not significant.

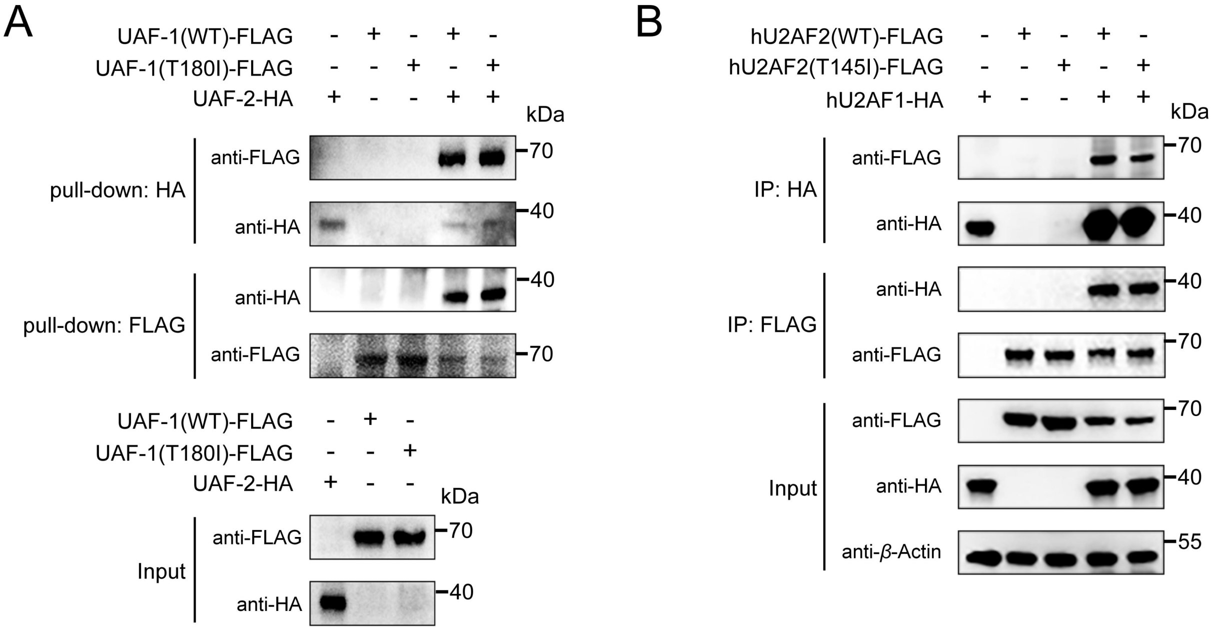

Figure S8. *C. elegans* UAF-1 T180I or human U2AF2 T145I mutations does not obviously affect the interaction between U2AF2 and wildtype U2AF1.

(A) Pull-down assay assessing the binding between *C. elegans* UAF-2(WT) (U2AF1 ortholog, expressed in HEK293T cells) and the recombinant FLAG-UAF-1 (U2AF2 ortholog, WT or T180I) purified from *E. coli*. Reciprocal pull-downs were performed and samples were immunoblotted using both anti-HA and anti-FLAG antibodies.

(B) Reciprocal co-immunoprecipitation of human FLAG-hU2AF2 (WT or T145I) and HA-hU2AF1(WT) overexpressed in HEK293T cells. β-actin was input loading control.

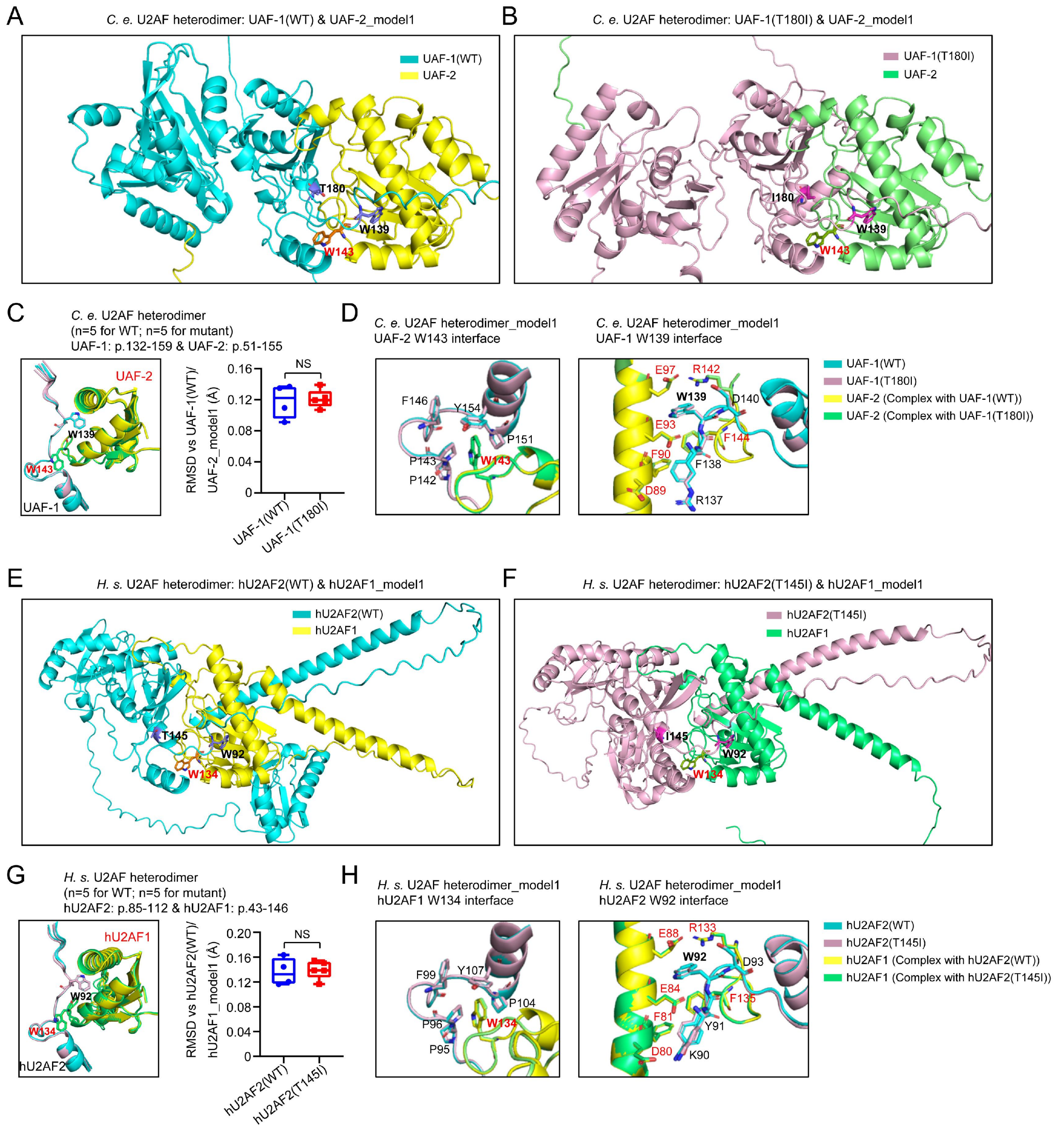

Figure S9. *C. elegans* UAF-1 T180I or human U2AF2 T145I does not obviously alter U2AF1 recognition in AlphaFold predicted structures.

(A) Model 1 of five AlphaFold predicted structures of the *C. elegans* UAF-1(WT)/UAF-2 (U2AF1 ortholog) heterodimer. UAF-1 T180 and W139 (orthologous to hU2AF2 T145 and W92) and UAF-2 W143 (orthologous to hU2AF1 W134) are highlighted.

(B) Model 1 of five AlphaFold predicted structures of the *C. elegans* UAF-1(T180I)/UAF-2 heterodimer. UAF-1 I180 and W139 and UAF-2 W143 are highlighted.

(C) Structural alignment of the predicted reciprocal tryptophan recognition regions (UAF-1: p.132-159; UAF-2: p51-155) between *C. elegans* UAF-1(WT)/UAF-2(WT) and UAF-1(T180I)/UAF-2(WT) heterodimers (left). All five wildtype and T180I structures were aligned. RMSD analysis was performed on the reciprocal tryptophan recognition regions in comparison to wildtype model 1 (right).
(D) Detailed views highlighting the region of reciprocal tryptophan recognition in the *C. elegans* U2AF heterodimer model 1. UAF-2 W143 and UAF-1 W139 are shown in bold. UAF-1(WT): cyan; UAF-1(T180I): pink; UAF-2 (complexed with UAF-1(WT)): yellow; UAF-2 (complexed with UAF-1(T180I)): green.
(E) Model 1 of five AlphaFold predicted structures of the human U2AF2(WT)/U2AF1 heterodimer. hU2AF2 T145, W92 and hU2AF1 W134 are highlighted.
(F) Model 1 of five AlphaFold predicted structures of the human U2AF2(T145I)/U2AF1 heterodimer. hU2AF2 I145 and W92 and hU2AF1 W134 are labeled.
(G) Structural alignment of the predicted reciprocal tryptophan recognition regions (U2AF2: p85-112; U2AF1: p43-146) between human U2AF2(WT)/U2AF1 and U2AF2(T145I)/U2AF1 heterodimers (left). All five wildtype and T180I structures were aligned. RMSD analysis was performed on the reciprocal tryptophan recognition regions in comparison to wildtype model 1 (right).
RMSD analysis was performed on the recognition regions in comparison to wildtype model 1 (right).
(H) Detailed views highlighting the region of reciprocal tryptophan recognition in the hU2AF heterodimer model 1. hU2AF1 W134 and hU2AF2 W92 are shown in bold. hU2AF2(WT): cyan; hU2AF2(T145I): pink; hU2AF1 (complexed with hU2AF2(WT)): yellow; hU2AF1 (complexed with hU2AF2(T145I)): green.
Note: PyMOL analyses-either excluding poorly matched atom pairs or including all atom pairs-did not reveal obvious RMSD differences in the reciprocal tryptophan recognition regions between wildtype and mutant heterodimers in *C. elegans* or human. The results obtained using the former approach are presented.

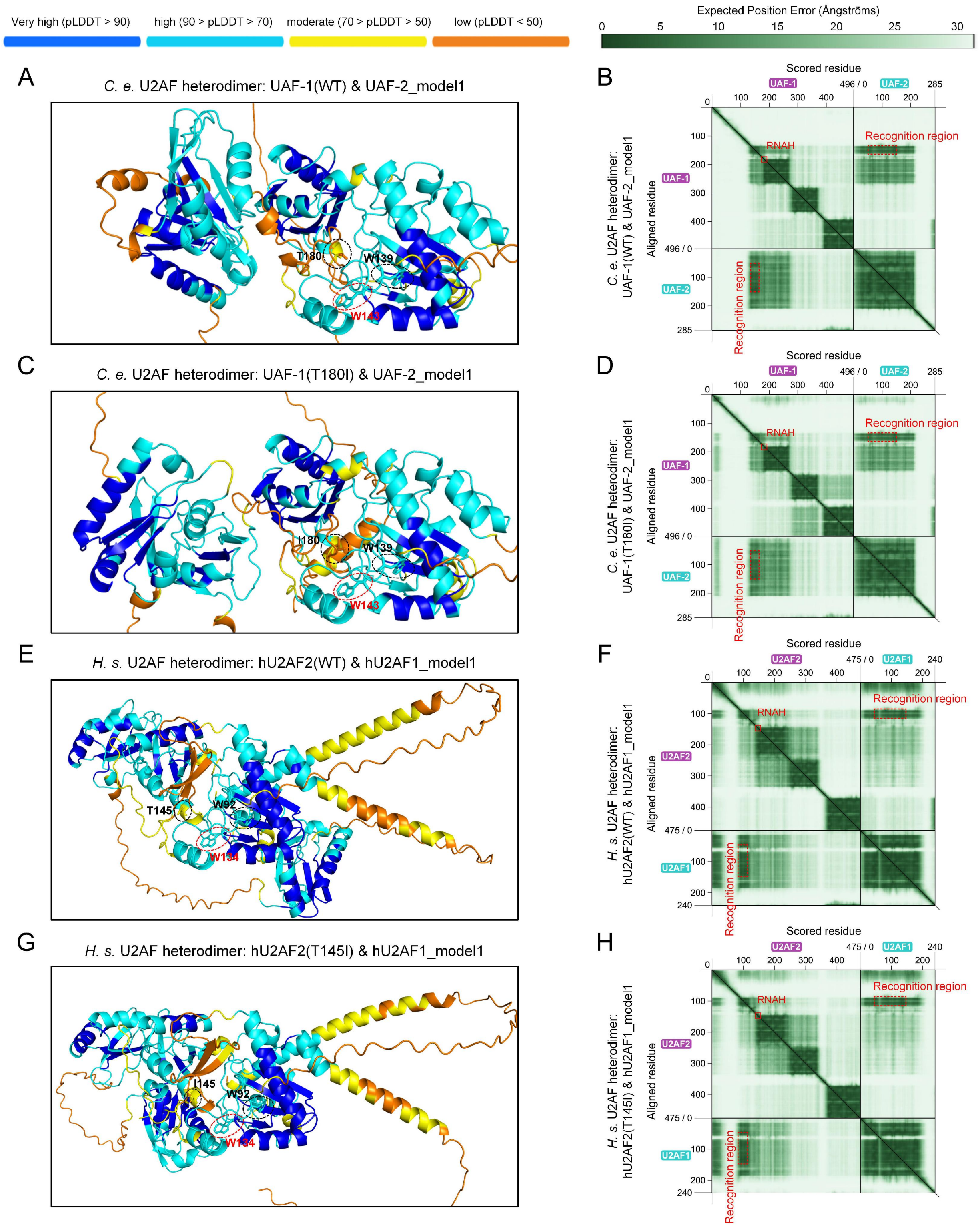

Figure S10. pLDDT scores and PAE plots of predicted U2AF heterodimer structures in *C. elegans* and human.

Only model 1 of each heterodimer was shown here. Models 2-5 are presented in Raw Data: AlphaFold Predictions. pLDDT scores (left panels) of residues are color coded for confidence levels: very high (blue, pLDDT > 90), high (cyan, 90 > pLDDT > 70), moderate (yellow, 70 > pLDDT > 50), and low (orange, pLDDT < 50). The PAE plot corresponding to each structure is shown in the right panels. Red solid squares indicate the RNAH motifs, and red dashed squares indicate the reciprocal tryptophan recognition regions of the heterodimer.

(A) *C. elegans* UAF-1(WT)/UAF-2 heterodimer.

(B) The PAE plot corresponding to (A).

(C) *C. elegans* UAF-1(T180I)/UAF-2 heterodimer.

(D) The PAE plot corresponding to (C).

(E) hU2AF2(WT)/hU2AF1 heterodimer.

(F) The PAE plot corresponding to (E).

(G) hU2AF2(T145I)/hU2AF1 heterodimer.

(H) The PAE plot corresponding to (G).

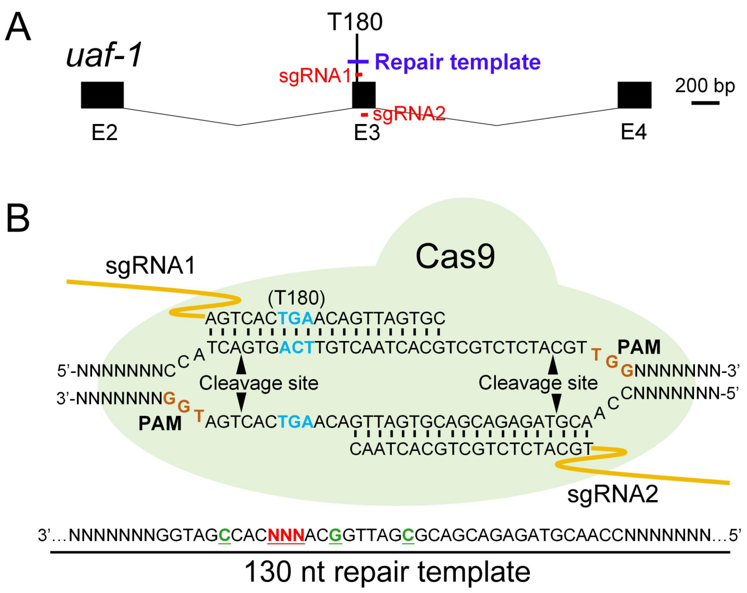

Figure S11. Generation of *uaf-1(T180X)* knockin mutations using CRISPR/Cas9.

(A) Exon-intron structures of the *uaf-1* gene (designed using the Exon-Intron Graphic Maker software at www.wormweb.org). The third exon, in which T180 is encoded, was targeted. sgRNAs are shown as red bars. The repair template is shown as the blue line above exon 3.

(B) Key components of the CRISPR/Cas9-mediated *T180X* mutagenesis. *sgRNA1* base pairs with the plus strand of *uaf-1* exon 3, in which ACT (blue) encodes T180, while *sgRNA2* base pairs with the minus strand. The PAM sequences are colored in brown and the expected cleavage sites are indicated with arrowheads. The central sequence of the 130 nt synthesized single-strand repair template is shown at the bottom. NNN (red) represents codons for different amino acids, and three synonymous mutations (green) are introduced to the template to minimize secondary cleavage.

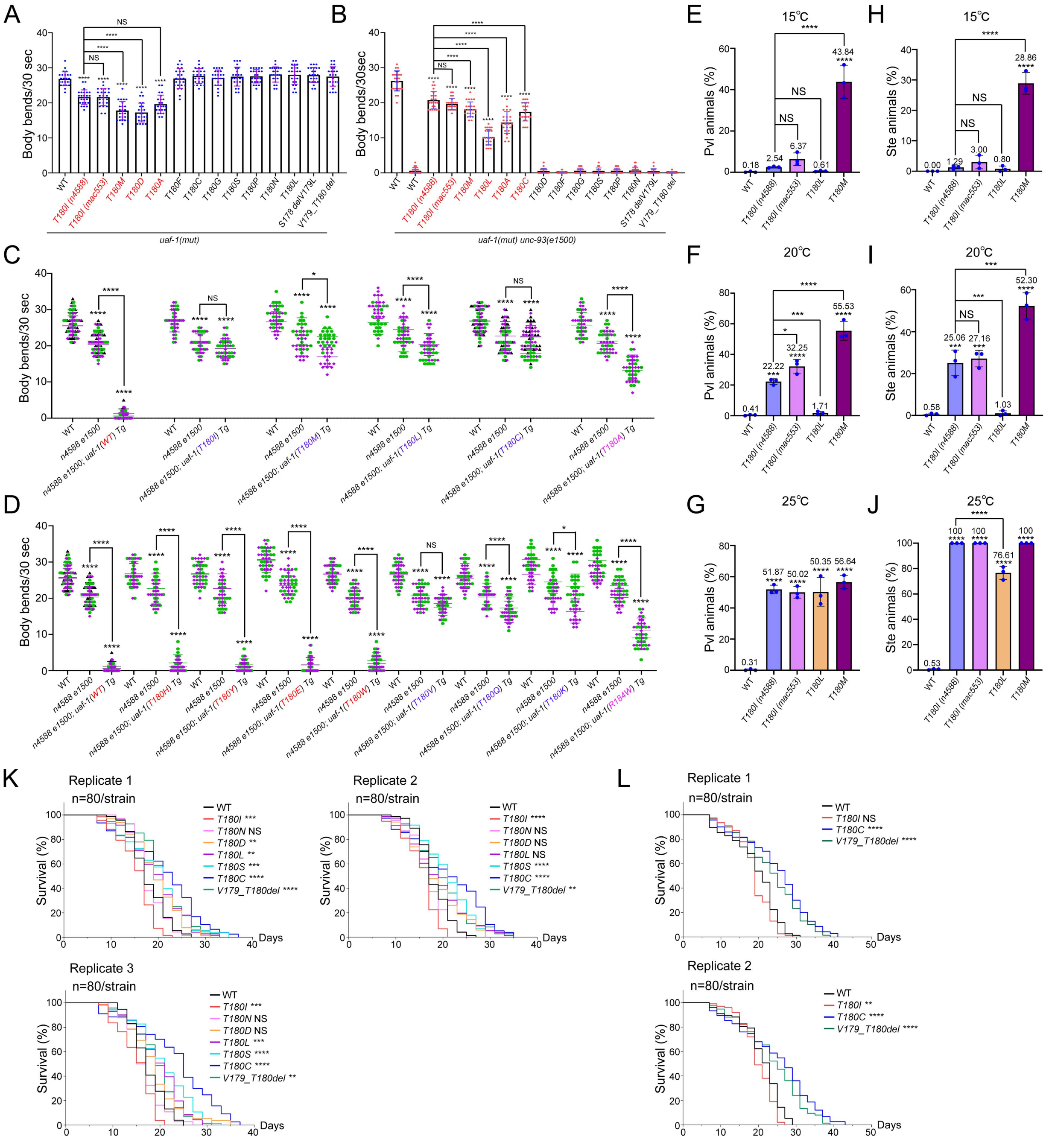

Strain		WT	*T180I (n4588)*	*T180N*	*T180D*	*T180L*	*T180S*	*T180C*	*V179_T180del*
Median survival days	Replicate 1	17	**17**	17	**19**	**21**	**21**	**23**	**21**
	Replicate 2	17	**17**	17	17	19	**21**	**21**	**19**
	Replicate 3	17	**17**	16	19	**21**	**21**	**25**	**19**

Strain		WT	*T180I (n4588)*	*T180C*	*V179_T180del*
Median survival days	Replicate 1	21	19	**27**	**25**
	Replicate 2	23	**19**	**27**	**25**

Figure S12. *uaf-1(T180X)* mutations cause distinct phenotypes in *C. elegans*.

(A) Locomotion (body bends/30 sec) of viable *uaf-1(T180X)* mutants (n=24 for each strain). Statistics: Tukey's multiple comparison test with one-way ANOVA. ****: p < 0.0001. NS: not significant.

(B) Locomotion of *uaf-1(T180X) unc-93(e1500)* double mutants (n=24 for each strain). Statistics: Tukey's multiple comparison test with one-way ANOVA. ****: $p < 0.0001$. NS: not significant.

(C) *uaf-1* transgene rescue of the suppression of *unc-93(e1500)* locomotion defect by *uaf-1(n4588)*. Two or three independent lines for each transgene were generated and 20 transgenic animals of each line or non-transgenic animals in replicates (colored dots in each column) of the indicated genotypes were analyzed. Statistics: Tukey's multiple comparison test with one-way ANOVA. *: $p < 0.05$; ****: $p < 0.0001$. NS: not significant.

(D) *uaf-1(T180X)* (knockin mutants of which unavailable) and *uaf-1(R184W)* transgene rescue of the suppression of *unc-93(e1500)* locomotion defect by *uaf-1(n4588)*. Two or three independent lines for each transgene were generated and 20 transgenic animals of each line or non-transgenic animals in replicates (colored dots in each column) of the indicated genotypes were analyzed. Statistics: Tukey's multiple comparison test with one-way ANOVA. *: $p < 0.05$; ****: $p < 0.0001$. NS: not significant.

(E-G) Synchronized L1 larva were grown to adults at 15°C, 20°C, and 25°C and their phenotypes were analyzed. The percentages of Pvl animals were indicated at the top.

(H-J) The same adults in (E-G) were assessed for the Ste phenotype. Results were based on three biological replicates and ~100 animals were analyzed in each replicate. Statistics: Tukey's multiple comparison with one-way ANOVA. *: $p < 0.05$; ***: $p < 0.001$; ****: $p < 0.0001$. Error bars: standard deviation.

(K) Lifespans of the wild type and seven *uaf-1(T180X)* mutants. Survived animals were quantified every two days starting on day three of synchronized L4 animals. Three biological replicates (n=80 per strain) were performed and the median lifespans are shown at the bottom.

(L) Replicate lifespan assays on the wild type and three *uaf-1(T180X)* mutants. Statistics: log-rank test. **: $p < 0.01$; ***: $p < 0.001$; ****: $p < 0.0001$. NS: not significant.

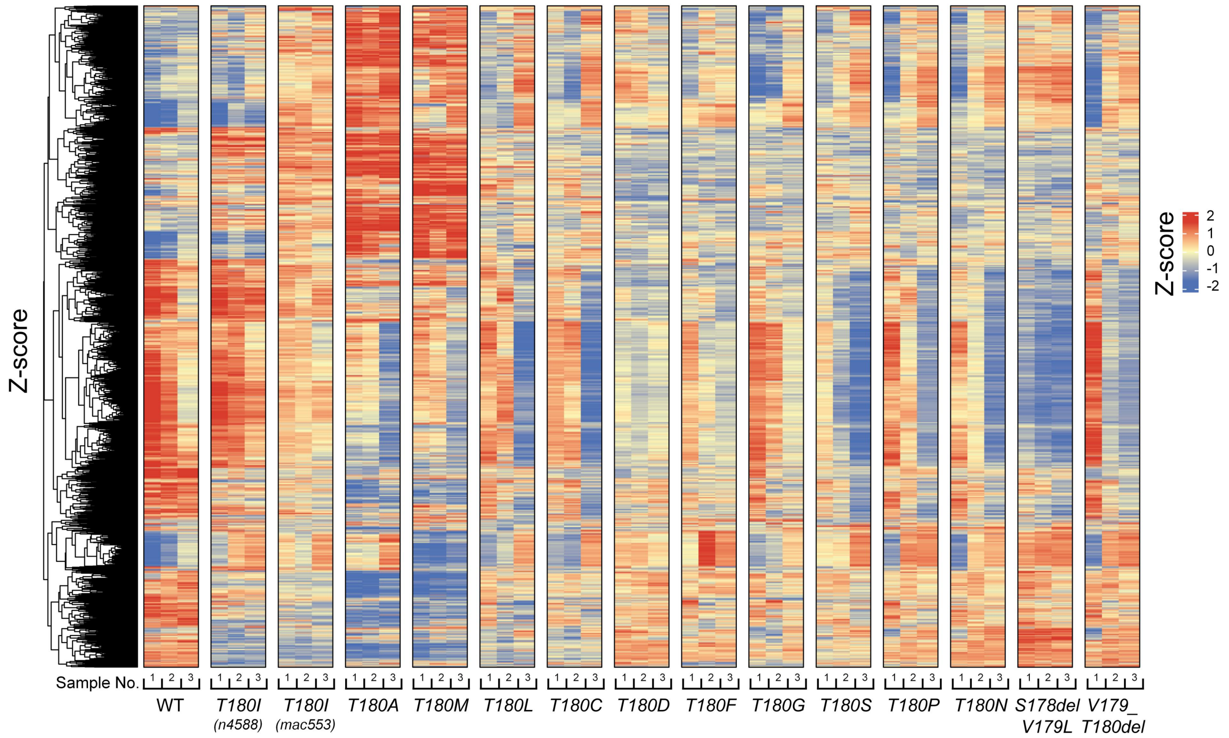

Figure S13. Heat map of differentially expressed genes (DEGs) in *uaf-1(T180X)* mutants at the L4 stage.

Compared to the wild type, the numbers of DEGs in the mutants fitting the criteria of $p < 0.05$, $q < 0.01$ and |log2FoldChange| > 1 were 256 (*T180I)(n4588*), 286 (*T180I)(mac553),* 884 (*T180A*), 1170 (*T180M*), 57 (*T180L*), 65 (*T180C*), 268 (*T180D*), 159 (*T180F*), 42 (*T180G*), 113 (*T180S*), 39 (*T180P*), 28 (*T180X*), 1027 (*S178delV179L*), and 28 (*V179_T180del*).

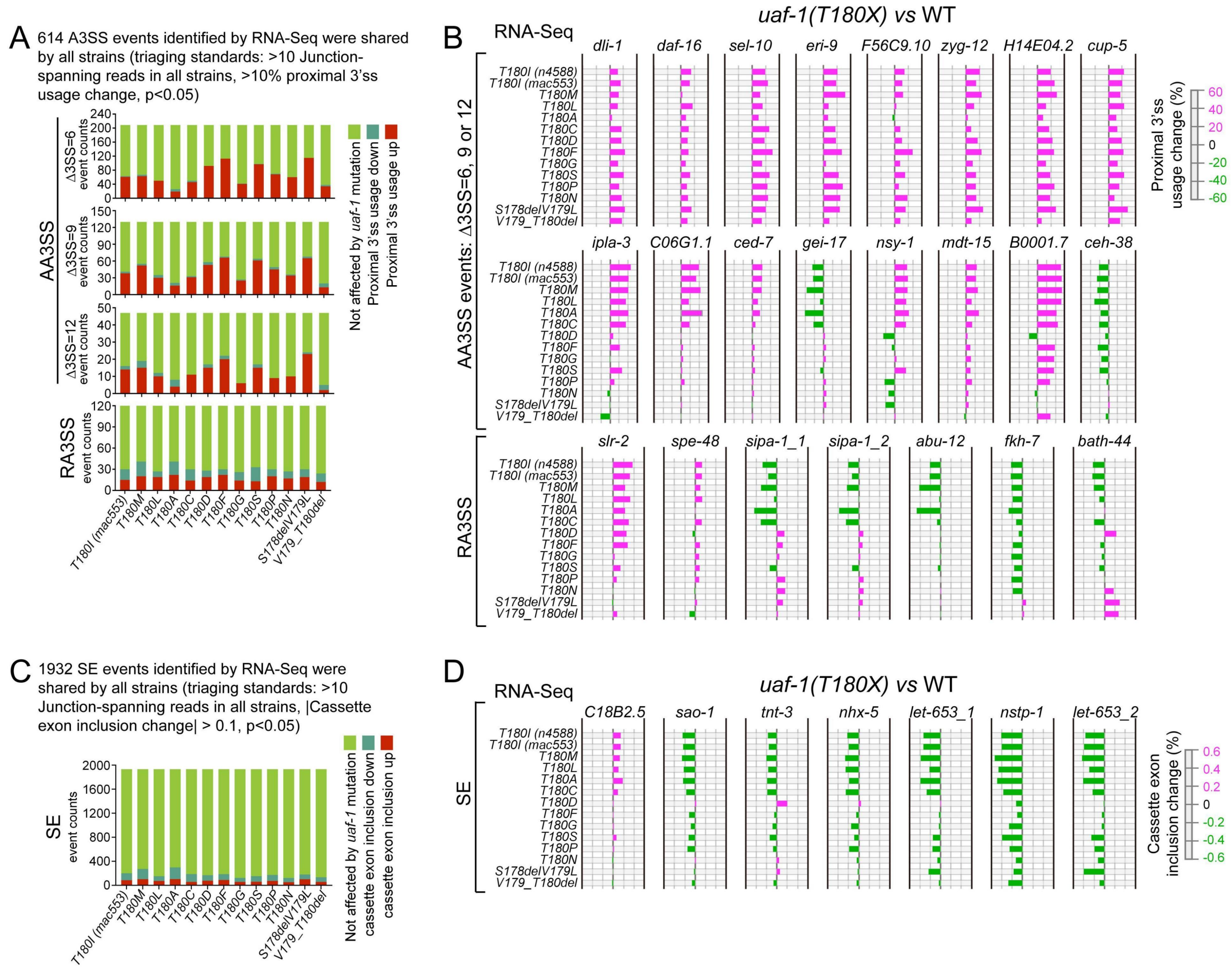

Figure S14. *uaf-1(T180X)* mutations differentially regulate distinct alternative splicing events.

(A) The number of A3SS events affected by *uaf-1(T180X)* mutations (>10% proximal 3'ss usage change, $p < 0.05$). Results were based on 614 shared events that have more than 10 junction-spanning reads.

(B) Representative A3SS events showing the diverse effects of *T180X* mutations on the proximal 3'ss usage using the wild type as reference.

(C) The numbers of SE events affected by *uaf-1(T180X)* mutations (|Cassette exon inclusion change| > 0.1, $p < 0.05$). Results were based on 1932 shared events with more than 10 junction-spanning reads.

(D) Representative SE events showing the diverse effects of *T180X* mutations on the cassette exon inclusion using the wild type as reference.

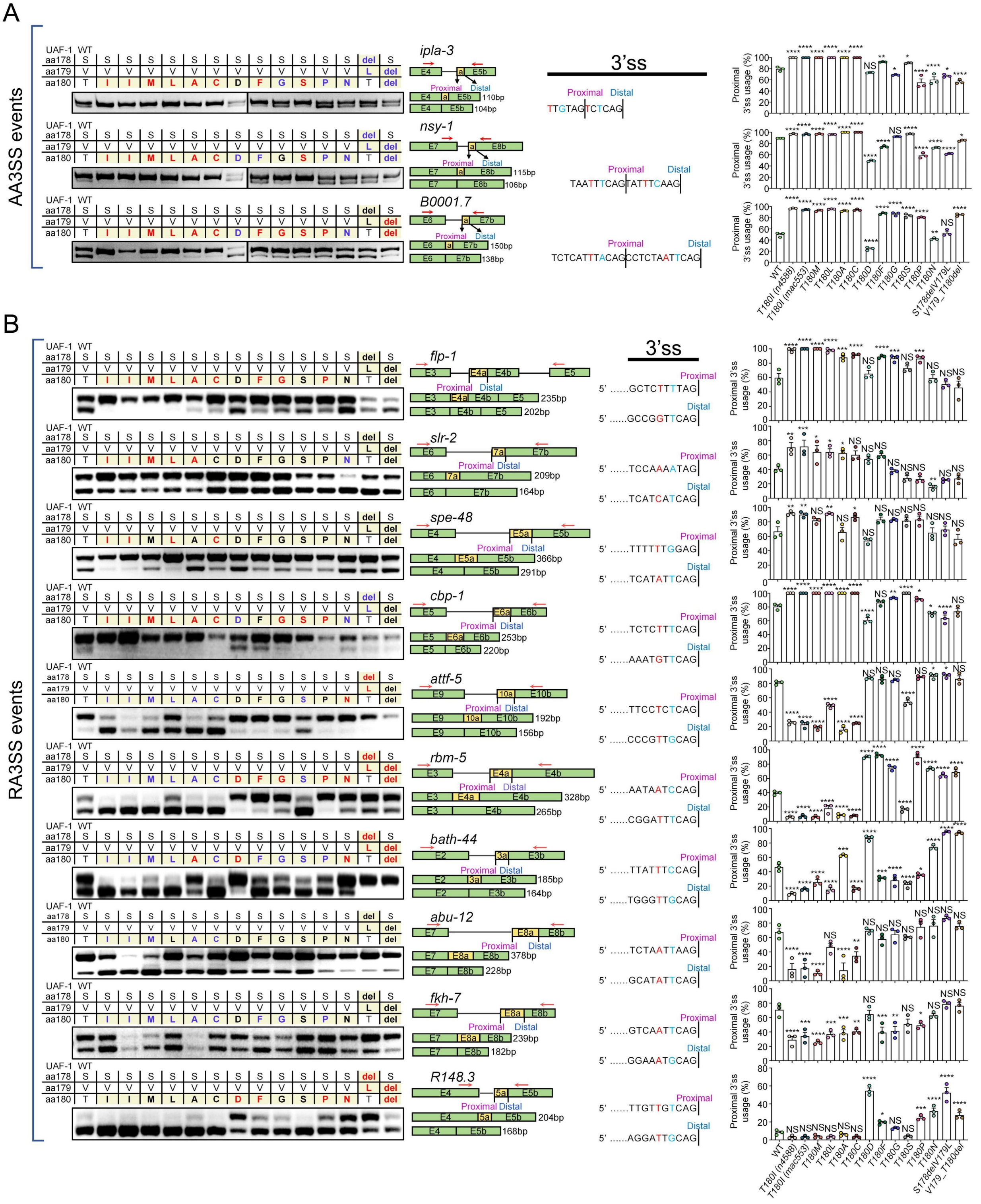

Figure S15. RT-PCR validation of representative AA3SS and RA3SS events affected by *uaf-1(T180X)* mutations at the L4 stage.

(A) For AA3SS events, PCR products were separated on a 20% non-denaturing polyacrylamide gel.

(B) For RA3SS events, PCR products were separated on a 2.5% agarose gel.

AA3SS and RA3SS event groups are indicated on the left. Gel pictures are shown on the left and the corresponding UAF-1 amino acid changes are indicated on the top. Mutations causing increased proximal 3’ss usage are highlighted in red while those causing decreased usage are in blue. Splice isoform structures and 3’ss sequences are shown in the middle with positions -6 and -4 marked in red and blue. Quantifications of proximal 3’ss usage are shown on the right. Results were based on three biological replicates. Statistics: Dunnett's multiple comparison test with one-way ANOVA. *: $p < 0.05$; **: $p < 0.01$; ***: $p < 0.001$; ****: $p < 0.0001$. NS: not significant.

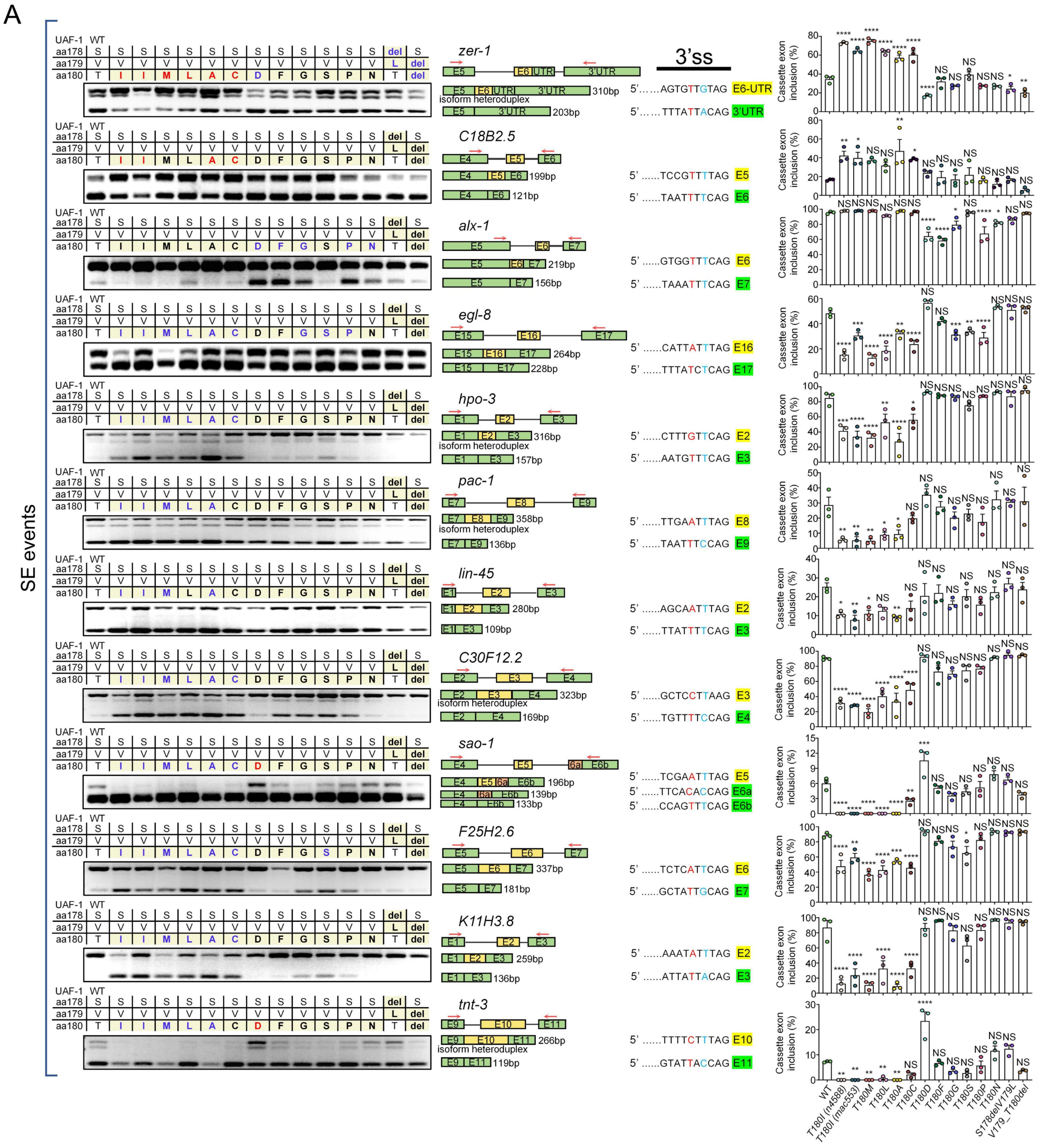
A
SE events
3'ss
zer-1
C18B2.5
alx-1
egl-8
hpo-3
pac-1
lin-45
C30F12.2
sao-1
F25H2.6
K11H3.8
tnt-3
UAF-1 WT
aa178
aa179
aa180
isoform heteroduplex
Cassette exon inclusion (%)
WT
T180I (n4588)
T180I (mac553)
T180M
T180L
T180A
T180C
T180D
T180F
T180G
T180S
T180P
T180N
S178delV179L
V179_T180del

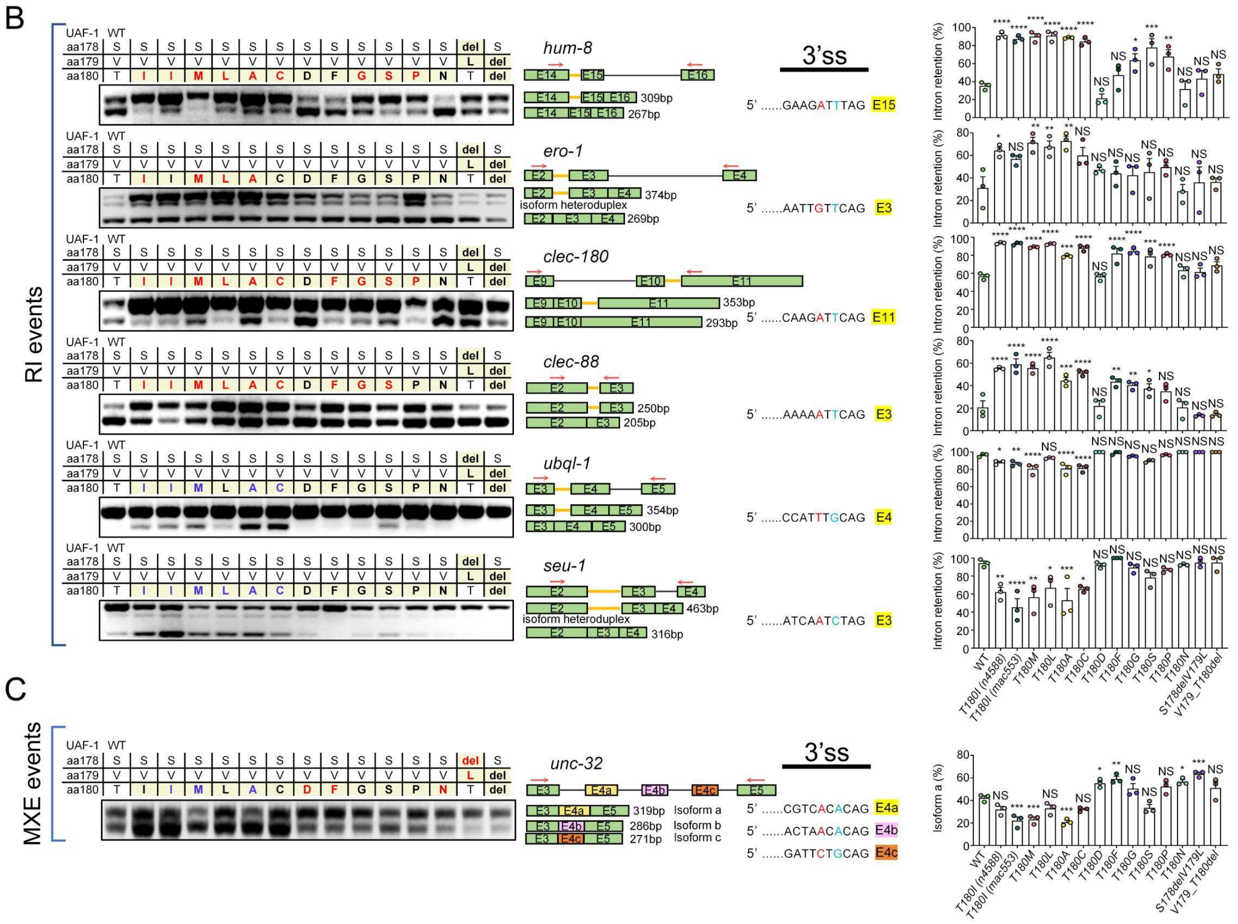

Figure S16. RT-PCR validation of representative SE, RI and MXE events affected by *uaf-1(T180X)* mutations at the L4 stage.

(A) SE, (B) RI and (C) MXE events are indicated on the left.

PCR products were separated on a 2.5% agarose gel. Gel pictures are shown on the left and the corresponding UAF-1 amino acid changes are indicated on the top. Mutations causing increased cassette exon inclusion, increased intron retention or the inclusion of *unc-32a* isoform are highlighted in red while those causing opposite effects are in blue. Splice isoform structures and 3’ss sequences are shown in the middle with positions -6 and -4 marked in red and blue. Quantifications of cassette exon inclusions, intron retention or the ratio of *unc-32a* splice isoform are shown on the right. Results were based on three biological replicates. Statistics: Dunnett's multiple comparison test with one-way ANOVA. *: $p < 0.05$; **: $p < 0.01$; ***: $p < 0.001$; ****: $p < 0.0001$. NS: not significant.

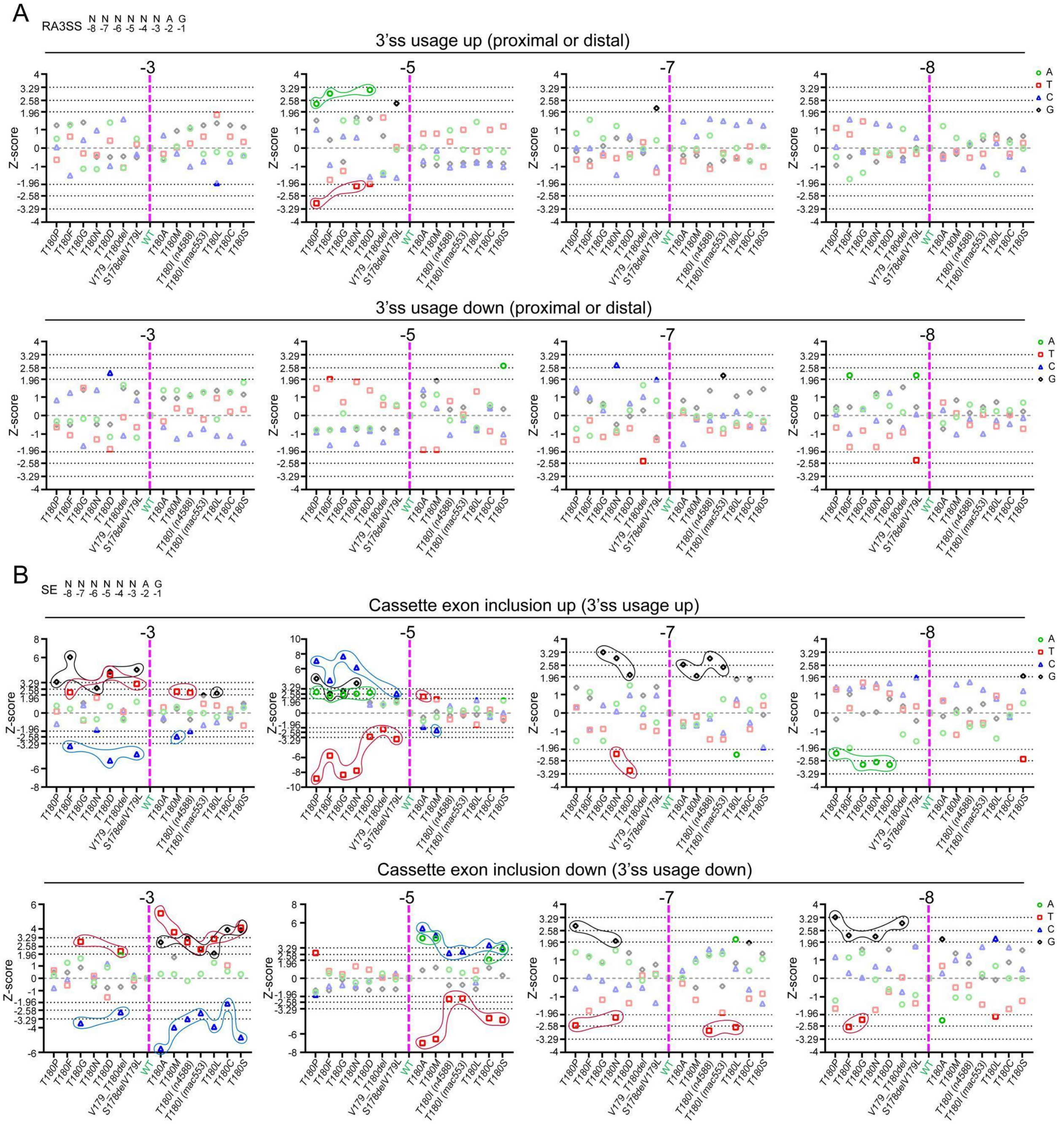

Figure S17: Significance of PPT nucleotide frequencies in RA3SS and SE events affected by *uaf-1(T180X)* mutations.

(A) Frequency significance of each nucleotide at positions -3, -5, -7 and -8 of the 3'ss in the affected RA3SS events.

(B) Frequency significance of each nucleotide at positions -3, -5, -7 and -8 of the 3'ss preceding the affected SE cassette exons.

Statistics: two-tailed Z-test. $|Z| > 1.96$: $p < 0.05$; $|Z| > 2.58$: $p < 0.01$; $|Z| > 3.29$: $p < 0.001$.

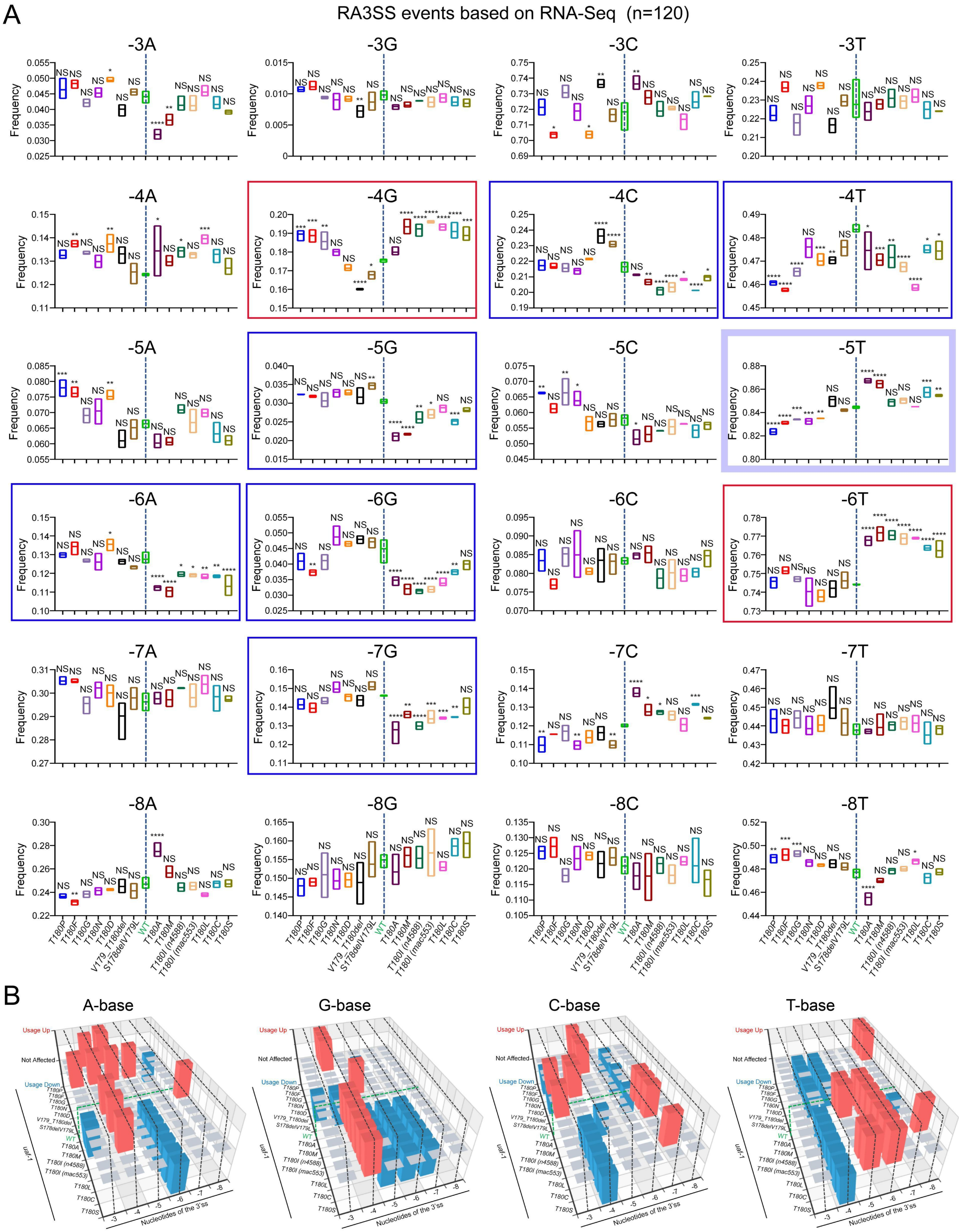
A
RA3SS events based on RNA-Seq (n=120)
-3A
-3G
-3C
-3T
-4A
-4G
-4C
-4T
-5A
-5G
-5C
-5T
-6A
-6G
-6C
-6T
-7A
-7G
-7C
-7T
-8A
-8G
-8C
-8T
Frequency
T180P
T180F
T180G
T180N
T180D
V179_T180del
S178delV179L
WT
T180A
T180M
T180I (n4588)
T180I (mac553)
T180L
T180C
T180S
B
A-base
G-base
C-base
T-base
Usage Up
Not Affected
Usage Down
uaf-1
Nucleotides of the 3'ss

Figure S18. Weighted frequencies of nucleotides at positions -3 to -8 of the 3’ss in RA3SS events and 3D graphs showing bidirectional associations of the frequencies with *uaf-1(T180X)* mutations.

(A) Nucleotides at positions -4, -5, -6 and -7 associated with increased or decreased proximal 3’ss usage in *uaf-1(T180I-like)* mutants are enclosed in red or blue boxes. Interestingly, -5T appeared to be associated with decreased proximal 3’ss usage in *uaf-1(T180D-like)* mutants (enclosed in thick blue line)

(B) Statistical significance of the weighted frequency of each nucleotide at positions -3 to -8 was plotted as red (increased frequency) or blue (decreased frequency) bars. Non-significant frequency is indicated with a gray plane.

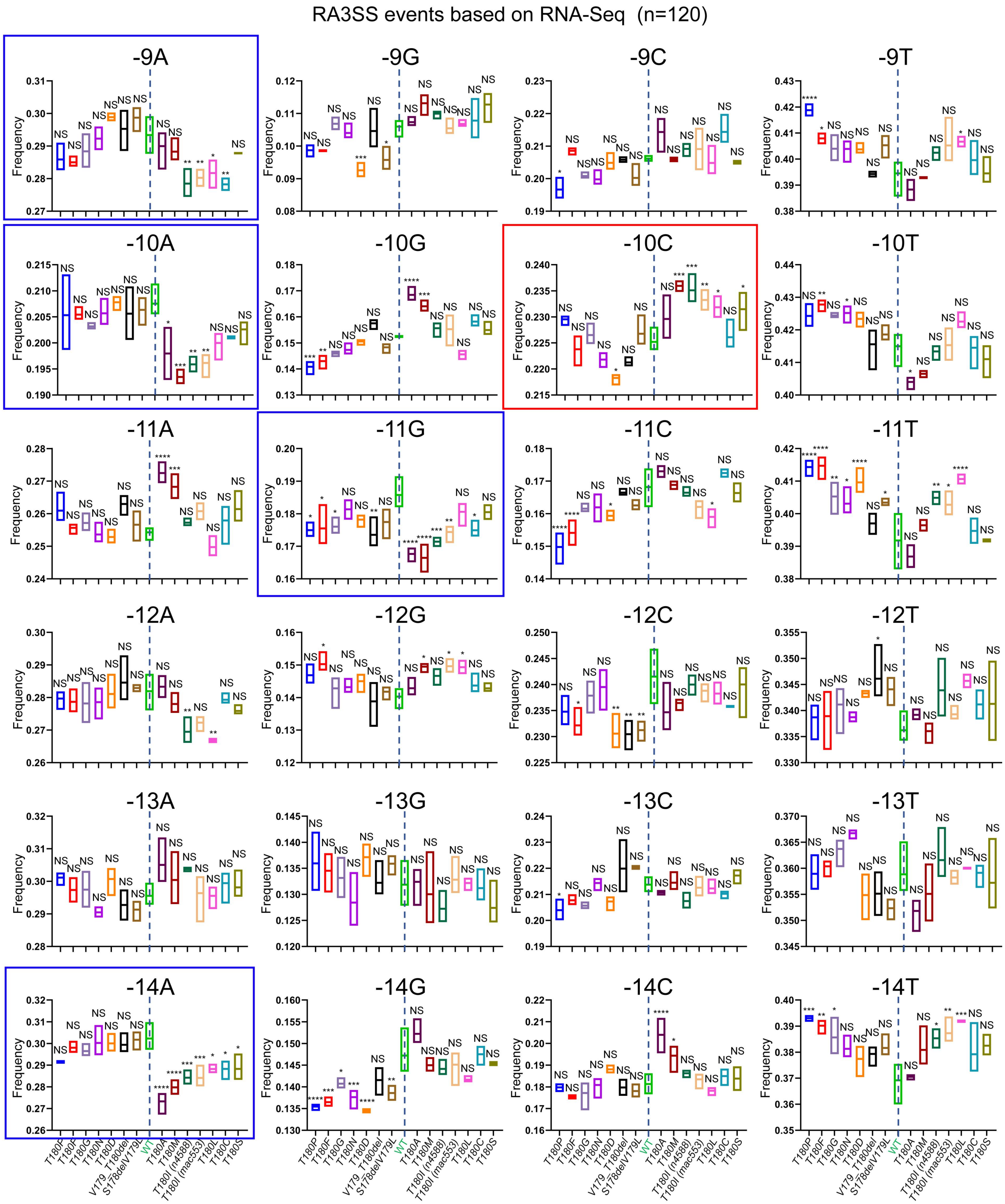

Figure S19. Weighted frequencies of nucleotides at positions -9 to -14 of the 3'ss in RA3SS events in *uaf-1(T180X)* mutations.

Nucleotides associated with increased or decreased proximal 3'ss usage in *uaf-1(T180I-like)* mutants are enclosed in red or in blue boxes.

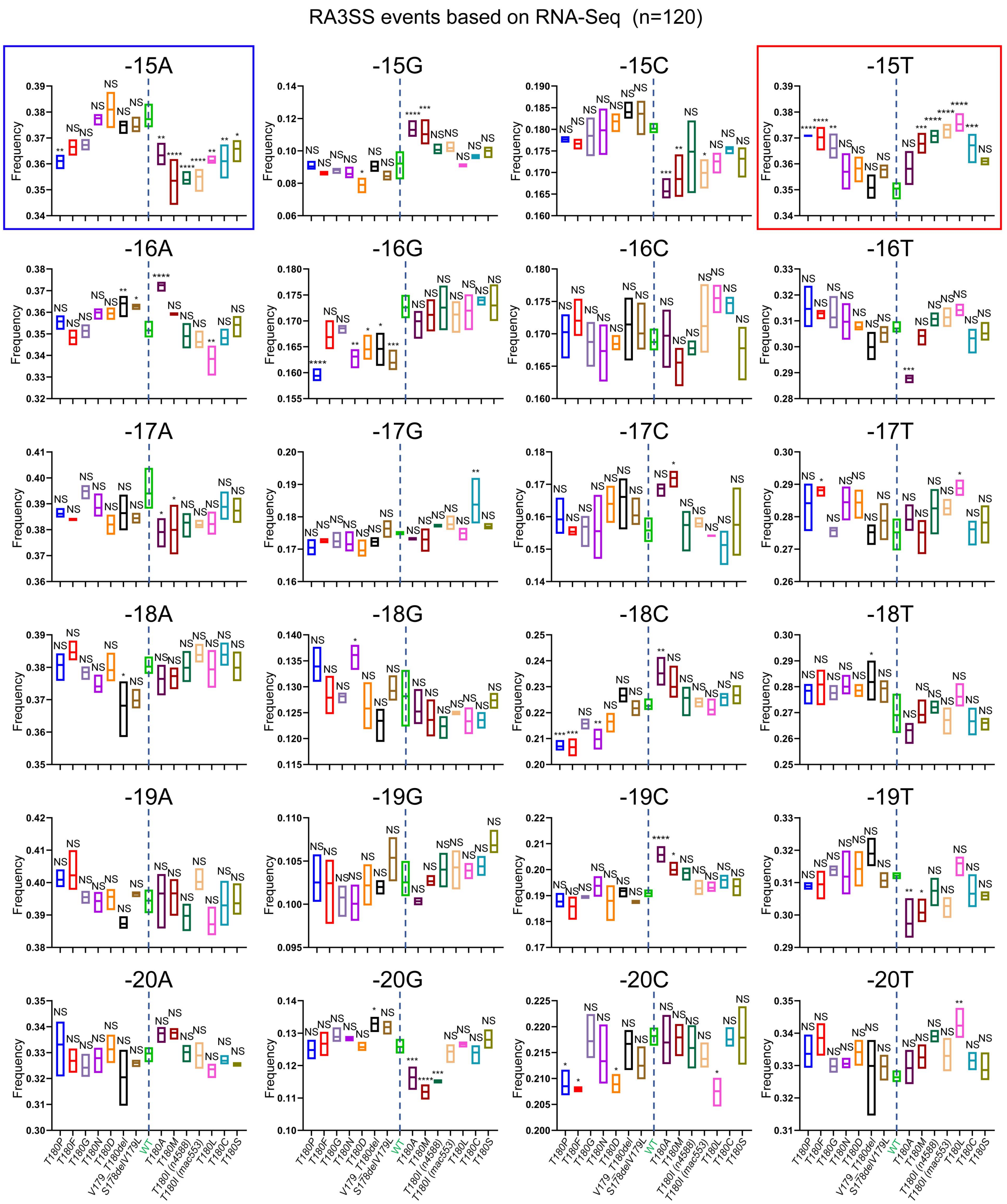

Figure S20. Weighted frequencies of nucleotides at positions -15 to -20 of the 3'ss in RA3SS events in *uaf-1(T180X)* mutations.

Nucleotides associated with increased or decreased proximal 3'ss usage in *uaf-1(T180I-like)* mutants are enclosed in red or in blue boxes.

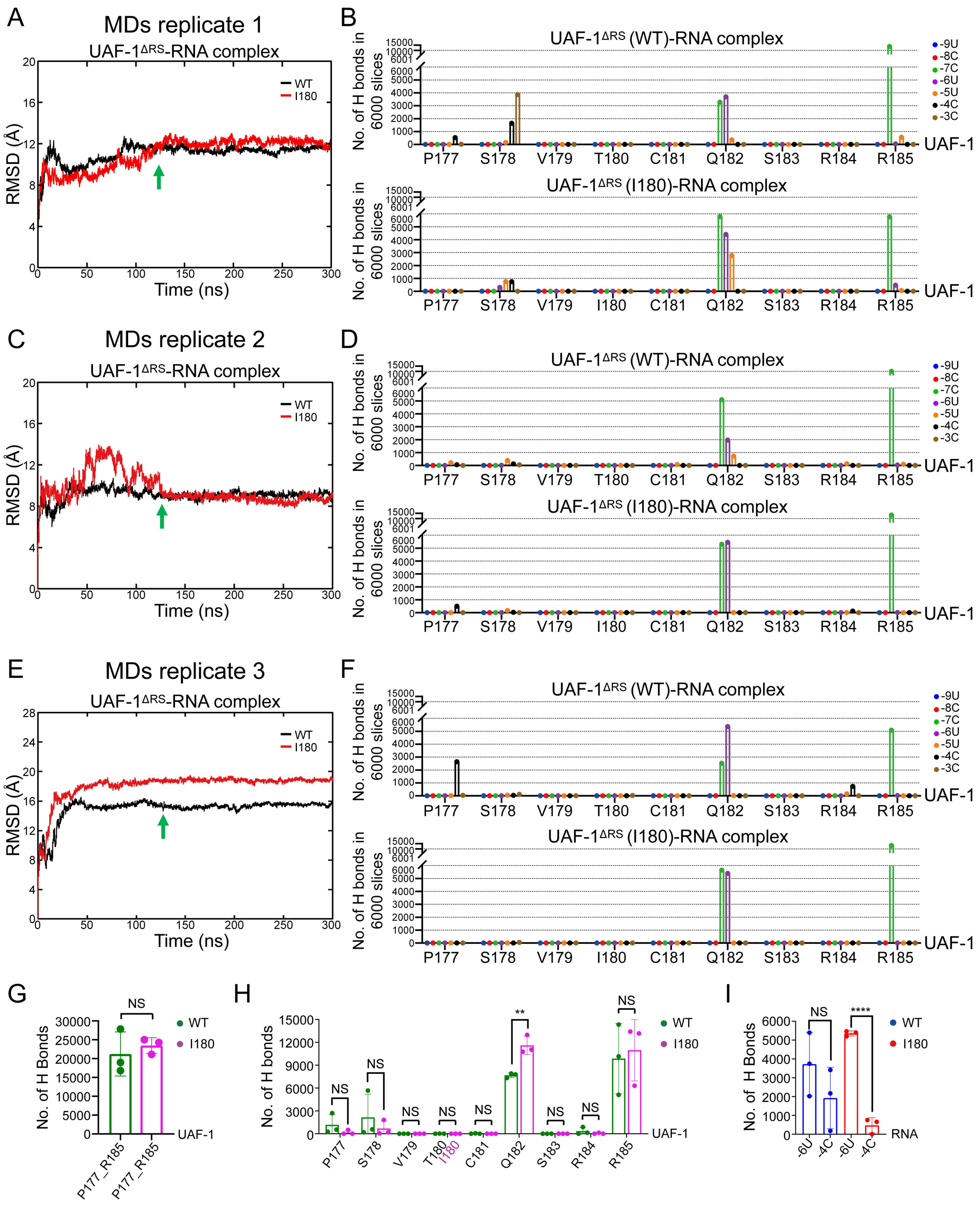

Figure S21. Molecular dynamics simulations predicts H bond formation between RNAH and the 3’ss RNA molecule.

(A, B) MDs replicate 1, showing that UAF-1$^{\Delta RS}$(WT)-RNA complex and UAF-1$^{\Delta RS}$(I180)-RNA complex reached similar conformations at ~125 ns of the simulation (A, green

arrow). The number of H bonds formed between residues of RNAH and the RNA molecule was obviously altered by the UAF-1 T180I mutation (B).

(C, D) MDs replicate 2, showing that UAF-$1^{\Delta RS}$(WT)-RNA complex and UAF-$1^{\Delta RS}$(I180)-RNA complex also reached similar conformations at ~125 ns of the simulation (C, green arrow). UAF-1 I180 caused obvious changes in H bond formation between RNAH and the RNA molecule (D).

(E, F) MDs replicate 3, showing that UAF-$1^{\Delta RS}$(WT)-RNA complex and UAF-$1^{\Delta RS}$(I180)-RNA complex exhibited divergent structures (E) that were stabilized at ~125 ns of the simulation. Still UAF-1 I180 caused obvious changes in H bond formation between RNAH and the RNA molecule (F).

(G) Average numbers of H bonds formed by RNAH residues of wildtype or mutant UAF-$1^{\Delta RS}$ and RNA molecule across the three simulations.

(H) The number of H bonds formed by each RNAH residue with the RNA molecule.

(I) The number of total H bonds formed by -4C and -6U of the RNA molecule with RNAH residues.

Results are based on three MDs replicates. Statistics: Student's t-test. **: $p < 0.01$. ****: $p < 0.0001$. NS: not significant.

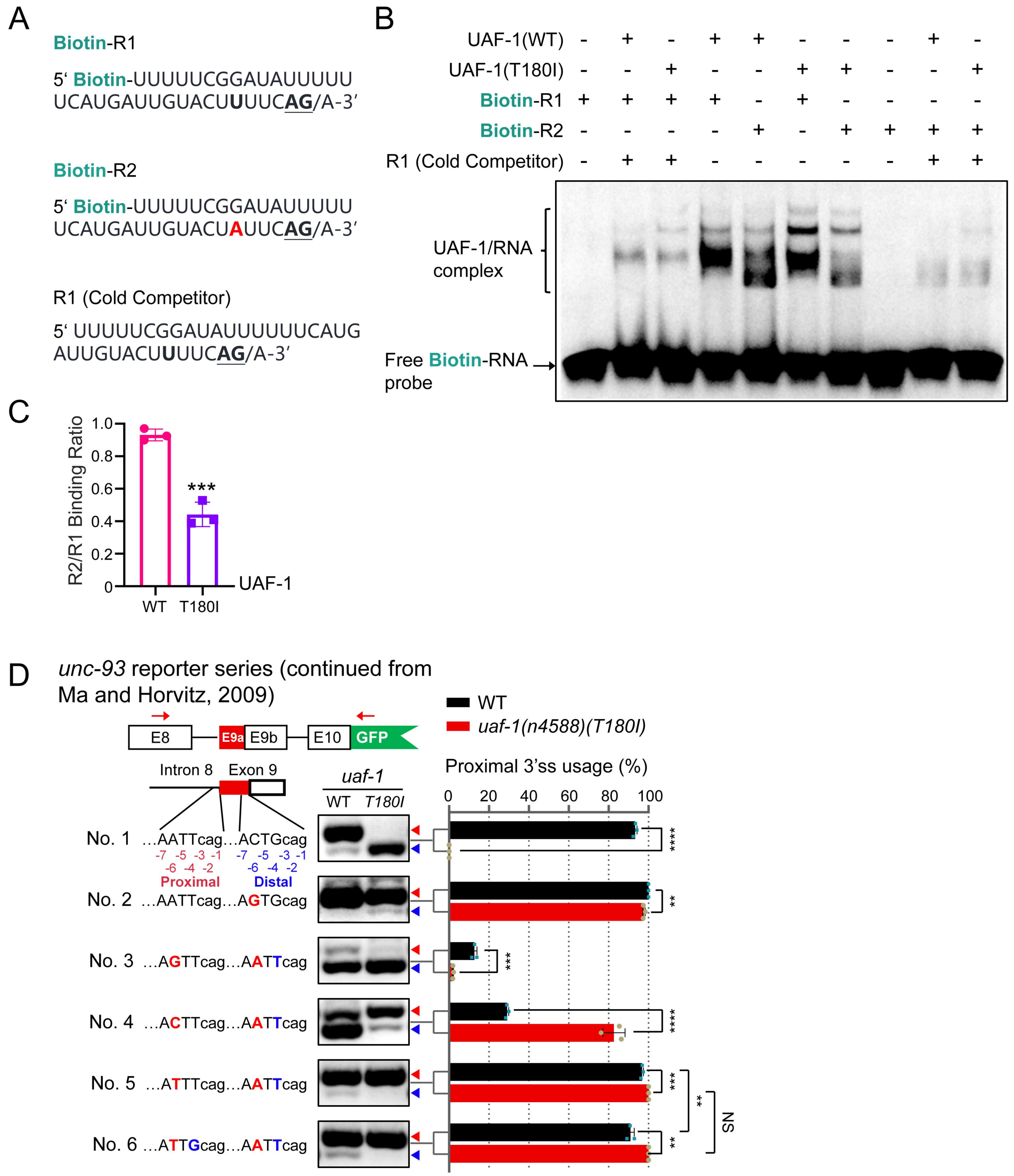

Figure S22: UAF-1 T180I reduces the binding to a non-consensus 3’ss RNA and affects alternative splicing involving nucleotides at positions -6 and/or -4.

(A) Sequences of 3'ss RNA probes for RNA electrophoretic mobility shift assay (EMSA, or gel shift). Biotin-R1 (consensus) and Biotin-R2 (non-consensus) probes are labeled

with 5' biotin. The R1 probe (consensus) is used as cold competition. The position of each nucleotide is indicated.

(B) Gel shift showing UAF-1 binding to the RNA probes, with or without the cold competitor. Components in each mixture indicated. The UAF-1-RNA complexes are marked.

(C) Intensity ratios between the UAF-1-R1 and UAF-1-R2 complex (R2/R1 ratio). Results are based on three replicate experiments. Statistics: Student's t-test. ***: $p < 0.001$.

(D) Exon-intron structure of a previously described *unc-93* splicing reporter (top left panel) (4). PCR primers for detecting splicing isoforms are shown as red arrows. PPT mutations are shown on the left in the corresponding positions. RT-PCR products of the reporters were analyzed on a 2.5% agarose gel, and representative results are shown in the middle panel (Red arrowhead: proximal 3'ss splice isoform; blue arrowhead: distal 3'ss splice isoform). Quantifications of proximal 3'ss usage are shown in the right panel. Results are based on three biological replicates. Statistics: Student's t-test. **: $p < 0.01$; ***: $p < 0.001$; ****: $p < 0.0001$; NS: not significant.

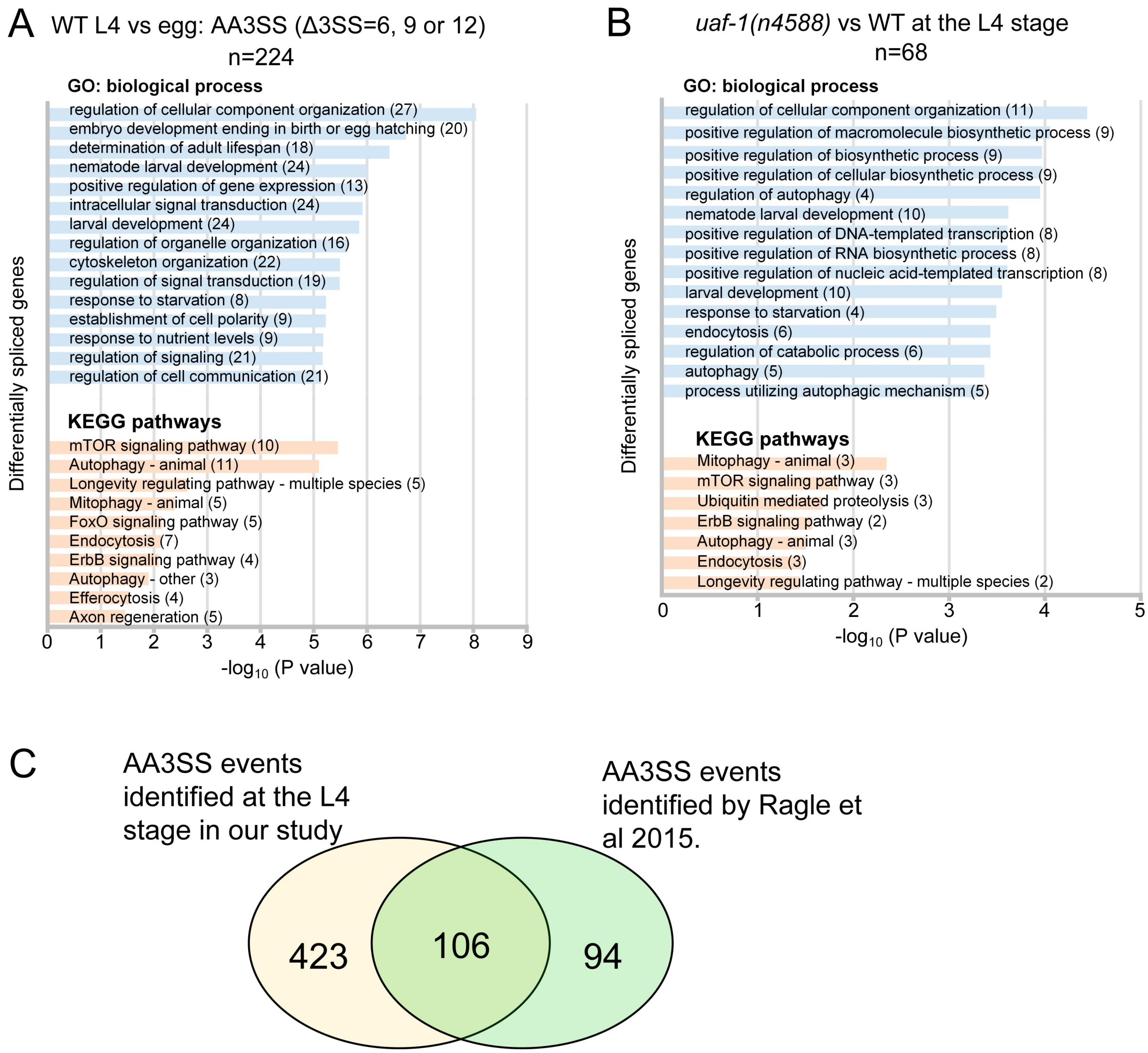

Figure S23: GO and KEGG enrichment of AA3SS events with embryo-to-L4 changes in proximal 3'ss usage.

(A) Genes encoding 224 AA3SS events (> 10% change in proximal 3'ss usage, $p < 0.05$) exhibiting embryo-to-L4 changes in proximal 3'ss usage are enriched in specific GO processes involved in cellular component organization, birth or egg hatching, and lifespan determination (top), *etc*. KEGG pathways such as mTOR signaling, autophagy and longevity are also significantly associated with these genes (bottom).

(B) 68 of the 224 AA3SS events were further affected by *uaf-1(n4588)* at the L4 larval stage (> 10% change in proximal 3'ss usage, $p < 0.05$). Genes encoding these events were significantly associated with GO processes (top) involved in cellular component

organization and biosynthetic process, *etc*. These genes are also significantly associated with KEGG pathways (bottom) such as mitophagy, mTOR signaling and ubiquitin mediated proteolysis.

(C) A Venn diagram showing a significant number of AA3SS events identified in this study were previously described by Ragle et al. (1).

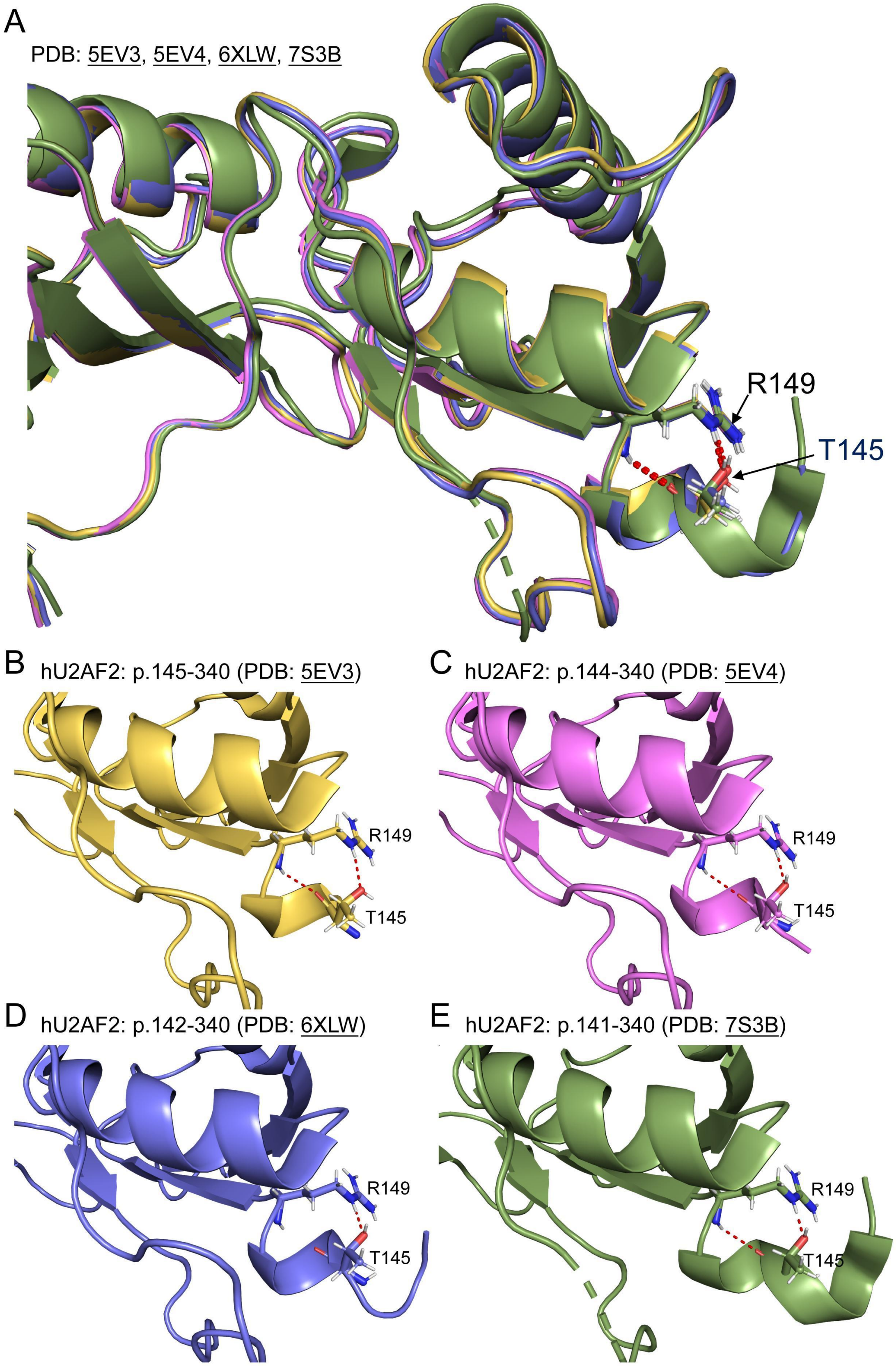
A
PDB: 5EV3, 5EV4, 6XLW, 7S3B
R149
T145
B
hU2AF2: p.145-340 (PDB: 5EV3)
R149
T145
C
hU2AF2: p.144-340 (PDB: 5EV4)
R149
T145
D
hU2AF2: p.142-340 (PDB: 6XLW)
R149
T145
E
hU2AF2: p.141-340 (PDB: 7S3B)
R149
T145

Figure S24. Previously resolved hU2AF2 crystal structures showing H bonds formed by the sidechains of T145 and R149.

(A) Alignment of four hU2AF2 1,2L crystal structures (PDB code: 5EV3, 5EV4, 6XLW, 7S3B) (3, 5). The sidechains T145 and R149 are shown. H bonds are represented by red dashed lines.

(B, C, D, E) Individual crystal structures of four hU2AF2 1,2L proteins. Portions of hU2AF2 used for the recombinant proteins are indicated. T145 and R149 are labeled. H bonds are represented by red dashed lines.

Table S1: List of *uaf-1* mutations generated via the CRISPR/Cas9 method.

***uaf-1* alleles**	**Mutation**	**Effect**
mac489	TCAGTGACTTGTCAATCA → TCGGTGGATTGCCAATCG	T180D
mac538	TCAGTGACTTGTCAATCA → TCGGTGTTTTGCCAATCG	T180F
mac539	TCAGTGACTTGTCAATCA → TCGGTGTGTTGTCAATCA	T180C
mac540	TCAGTGACTTGTCAATCA → TCGGTGGGATGCCAATCG	T180G
mac541	TCAGTGACTTGTCAATCA → TCGGTGGCCTGCCAATCA	T180A
mac542	TCAGTGACTTGTCAATCA → TCGGTGAGATGCCAATCG	T180R
mac543	TCAGTGACTTGTCAATCA → TCGGTGAGTTGTCAATCA	T180S
mac544	TCAGTGACTTGTCAATCA → TCGGTGCCATGCCAATCG	T180P
mac545	TCAGTGACTTGTCAATCA → TCGGTGAATTGTCAATCA	T180N
mac551	TCAGTGACTTGTCAATCA → TCGGTGATGTGCCAATCG	T180M
mac552	TCAGTGACTTGTCAATCA → TCGGTGTTGTGCCAATCA	T180L
mac553	TCAGTGACTTGTCAATCA → TCGGTGATCTGCCAATCA	T180I
mac548	3 bp del	S178delV179L
mac550	6 bp del	V179_T180del
mac546	1 bp ins	G194Rfs*197
mac547	47 bp del	V179Mfs*181
mac549	3 bp del	Y187del

In the mutation column, wildtype sequences are shown on the left and mutant sequences are on the right. Mutated nucleotides are highlighted in red. Synonymous nucleotides for minimizing second cleavage are in blue.

Table S2: The survival phenotype of *uaf-1* mutants at different temperatures.

uaf-1 alleles	Survival			
	15 °C	20 °C	22.5 °C	25 °C
WT	Yes	Yes	Yes	Yes
T180I (n4588)	Yes	Partial lethality	Lethality	Lethality
T180I (mac553)	Yes	Partial lethality	Lethality	Lethality
T180M	Partial lethality	Partial lethality	Lethality	Lethality
T180L	Yes	Yes	Partial lethality	Partial lethality
T180A	Sterility	Sterility	Sterility	Sterility
T180R	L1~L3 arrest	L1~L3 arrest	L1~L3 arrest	L1~L3 arrest
Y187del	L1 arrest	L1 arrest	L1 arrest	L1 arrest
*G194Rfs*197*	L1 arrest	L1 arrest	L1 arrest	L1 arrest
*V179Mfs*181*	L1 arrest	L1 arrest	L1 arrest	L1 arrest
T180C	Yes	Yes	Yes	Yes
T180F	Yes	Yes	Yes	Yes
T180D	Yes	Yes	Yes	Yes
T180G	Yes	Yes	Yes	Yes
T180S	Yes	Yes	Yes	Yes
T180P	Yes	Yes	Yes	Yes
T180N	Yes	Yes	Yes	Yes
S178delV179L	Yes	Yes	Yes	Yes
V179_T180del	Yes	Yes	Yes	Yes

Table S3: Effects of *uaf-1* mutations and transgenes on the “rubberband” phenotype of *unc-93(e1500)* mutants.

Genotype	Transgene	Tg lines	Rubberband phenotype
unc-93(e1500)	-	-	None
uaf-1(T180D) unc-93(e1500)	-	-	Strong
uaf-1(T180F) unc-93(e1500)	-	-	Strong
uaf-1(T180G) unc-93(e1500)	-	-	Strong
uaf-1(T180S) unc-93(e1500)	-	-	Strong
uaf-1(T180P) unc-93(e1500)	-	-	Strong
uaf-1(T180N) unc-93(e1500)	-	-	Strong
uaf-1(S178delV179L) unc-93(e1500)	-	-	Strong
uaf-1(V179_T180 del) unc-93(e1500)	-	-	Strong
uaf-1(T180I) (n4588) unc-93(e1500)	-	-	None
uaf-1(T180I) (mac553) unc-93(e1500)	-	-	None
uaf-1(T180M) unc-93(e1500)	-	-	None
uaf-1(T180L) unc-93(e1500)	-	-	None
uaf-1(T180C) unc-93(e1500)	-	-	None
uaf-1(T180A) unc-93(e1500)	-	-	None
uaf-1(n4588) unc-93(e1500)	*Pmyo-3::uaf-1a cDNA(**WT**)*	3	Strong (3/3)
	*Pmyo-3::uaf-1a cDNA(**T180I**)*	2	None (2/2)
	*Pmyo-3::uaf-1a cDNA(**T180M**)*	2	None (2/2)
	*Pmyo-3::uaf-1a cDNA(**T180L**)*	2	None (2/2)
	*Pmyo-3::uaf-1a cDNA(**T180C**)*	3	None (3/3)
	*Pmyo-3::uaf-1a cDNA(**T180A**)*	2	None (2/2)
	*Pmyo-3::uaf-1a cDNA(**T180H**)*	2	Strong (2/2)
	*Pmyo-3::uaf-1a cDNA(**T180Y**)*	2	Strong (2/2)
	*Pmyo-3::uaf-1a cDNA(**T180E**)*	2	Strong (2/2)
	*Pmyo-3::uaf-1a cDNA(**T180W**)*	2	Strong (2/2)
	*Pmyo-3::uaf-1a cDNA(**T180V**)*	2	None (2/2)
	*Pmyo-3::uaf-1a cDNA(**T180Q**)*	2	None (2/2)
	*Pmyo-3::uaf-1a cDNA(**T180K**)*	2	None (2/2)
	*Pmyo-3::uaf-1a cDNA(**R184W**)*	2	weak (2/2)

For *uaf-1(T180X)* mutations whose knockin mutants were not available, respective *uaf-1(T180X)* mutant transgenes were examined for rescuing the suppression of *unc-93(e1500)* phenotype by *uaf-1(n4588)*.

Table S4. ***rbm-5*** **mutations partially suppress the** ***ts*****-lethality of** ***uaf-1(T180I-like)*** **mutants.**

Genotype	Survival			
	**15 °C**	**20 °C**	**22.5 °C**	**25 °C**
WT	Yes	Yes	Yes	Yes
rbm-5(n5119lf)	Yes	Yes	Yes	Yes
rbm-5(n5132lf)	Yes	Yes	Yes	Yes
uaf-1(n4588) **T180I**	Yes	Partial Lethality	Lethality	Lethality
rbm-5(n5119lf); uaf-1(n4588)	Yes	Yes	Yes	Lethality
rbm-5(n5132lf); uaf-1(n4588)	Yes	Yes	Yes	Lethality
uaf-1(mac553) **T180I**	Yes	Partial Lethality	Lethality	Lethality
rbm-5(n5119lf); uaf-1(mac553)	Yes	Yes	Yes	Lethality
rbm-5(n5132lf); uaf-1(mac553)	Yes	Yes	Yes	Lethality
uaf-1(mac551) **T180M**	Partial Lethality	Partial Lethality	Lethality	Lethality
rbm-5(n5119lf); uaf-1(mac551)	Partial Lethality	Partial Lethality	Partial Lethality	Lethality
rbm-5(n5132lf); uaf-1(mac551)	Partial Lethality	Partial Lethality	Partial Lethality	Lethality
uaf-1(mac552) **T180L**	Yes	Yes	Partial Lethality	Partial Lethality
rbm-5(n5119lf); uaf-1(mac552)	Yes	Yes	Yes	Partial Lethality
rbm-5(n5132lf); uaf-1(mac552)	Yes	Yes	Yes	Partial Lethality

Table S5: Primers used for plasmid construction and CRISPR/Cas9-based mutagenesis.

Plasmids	Primer Name	Sequence (5'→3')
Pmyo-3::uaf-1a_cDNA(wt)::GFP	*BamHI-uaf-1* Forward	CGCGGATCCATGAGTGATCACCAGGATGG
	AgeI-uaf-1 Reverse	TCTACCGGTTGGAATTGACGATTGTGGTACTTG
Pmyo-3::uaf-1a_cDNA(T180X)::GFP	*uaf-1(T180X)* Forward	GCCCATCAGTG*NNN*TGTCAATCACGTCGTCTCTACG
	uaf-1(T180X) Reverse	CGTGATTGACA*NNN*CACTGATGGGCCAACAACGG
Pmyo-3::uaf-1a_cDNA(R184W)::GFP	*uaf-1(R184W)* Forward	GTCAATCA*TGG*CGTCTCTACGTTGGAAATATTCCG
	uaf-1(R184W) Reverse	CCAACGTAGAGACG*CCA*TGATTGACAAGTCACTGATGGGC
PCMV::hU2AF2_cDNA(wt)::FLAG	*BamHI-U2AF2* Forward	CGCGGATCCATGTCGGACTTCGACGAGTTC
	XhoI-U2AF2 Reverse	CCGCTCGAGCTACTTGTCATCGTCGTCCTTGTAATCCCAGAAGTCCCGGCGGTGATAAG
PCMV::hU2AF2_cDNA(T145I)::FLAG	*U2AF2(T145I)* Forward	GAGCCAGATG*ATT*AGACAAGCCCGGCGCCTCTACG
	U2AF2(T145I) Reverse	GGCTTGTCT*AAT*CATCTGGCTCCCGACCACGGGCAC
PCMV::hU2AF2_cDNA(R149W)::FLAG	*U2AF2(R149W)* Forward	CAGACAAGCC*TGG*CGCCTCTACGTGGGCAACATCC
	U2AF2(R149W) Reverse	TAGAGGCG*CCA*GGCTTGTCTGGTCATCTGGCTCCC
PCMV::hU2AF1_cDNA(wt)::HA	*BamHI-U2AF1* Forward	CGCGGATCCATGGCGGAGTATCTGGCCTCCATC
	XhoI-U2AF1 Reverse	CCGCTCGAGTTAAGCGTAGTCTGGGACGTCGTATGGGTAGAATCGCCCAGATCTTTCACG
PCMV::uaf-2_cDNA(wt)::HA	*BamHI-uaf-2* Forward	CGCGGATCCATGTCGTATGGTGACGGGC
	XhoI-uaf-2 Reverse	CCGCTCGAGTTAAGCGTAGTCTGGGACGTCGTATGGGTAATATCGTCTTCGATCACGGC
pET-28a(+) *His::uaf-1a_cDNA(wt)::FLAG*	*pET-uaf-1* Forward	ATGAGTGATCACCAGGATGG
	pET-uaf-1 Reverse	TCATCGTCGTCCTTGTAATCGAATTGACGATTGTGGTACTTGTCG
	uaf-1-pET Forward	GATTACAAGGACGACGATGACAAGTAAAAGCTTGCGGCCGCAC
	uaf-1-pET Reverse	CCATCCTGGTGATCACTCATATGGCTGCCGCGCGGCAC
pET-28a(+) *His::uaf-1a_cDNA(T180I)::FLAG*	*uaf-1(T180I)* Forward	GCCCATCAGTG*ATT*TGTCAATCACGTCGTCTCTACG
	uaf-1(T180I) Reverse	CGTGATTGACA*AAT*CACTGATGGGCCAACAACGG
pGint *GFP-hNFRKB(I5-E6-I6)*	*BamHI-hNFRKB* Forward	CGCGGATCCTGGTAGCCATTTATCTCCCCAGC
	SalI-hNFRKB Reverse	ACGCGTCGACAAGGCAGGCCTATCTCCAGATG
Pmyo-3::attf-5(E9-I9-E10)	*BamHI-attf-5* Forward	CGCGGATCCATGCACACCACCACCAGTCAAAAAAAC
	AgeI-attf-5 Reverse	TCTACCGGTCTTATCTTGTTCCCTATCCATAGC
Pmyo-3::attf-5(E9-I9-E10 mut)	*attf-5(mut)* Forward	GATTCCCG*N*T*N*CAGAAACTAAACTGAACCC
	attf-5(mut) Reverse	TAGTTTCTG*N*A*N*CGGGAATCAGTGCACC
Punc-93::unc-93(E8-I8-E9-I9-E10 proximal mut)	*unc-93(proximal mut)* Forward	TTAATCAAGCCA*NN*T*N*CAGATTTTTCGG
	unc-93(proximal mut) Reverse	CCGAAAAATCTG*N*A*NN*TGGCTTGATTAA
Punc-93::unc-93(E8-I8-E9-I9-E10 distal mut)	*unc-93(distal mut)* Forward	GATTGTAC*NN*T*N*CAGACAAGTCGTCGGAAATA
	unc-93(distal mut) Reverse	CTTGTCTG*N*A*NN*GTACAATCATGAAAAAATATCCG
**CRISPR related**	**Sequences**	
uaf-1 guideRNA1	CGTGATTGACAAGTCACTGA	
uaf-1 guideRNA2	CAATCACGTCGTCTCTACGT	
uaf-1 repair 130 mer oligo	ttgttgattgaaaaagtcgagcatagcttcttcattgcatccgaacggaatatttccaacgtagagacgacg**C**gattg**G**ca***NNN***cac**C**gatgggcctaaaaaatgagaaaaatttgaaaatttgggcagc	

Table S6. RT-PCR primer sequences for validating A3SS events.

	RT-PCR primers for detecting alternative splicing		3'ss sequences	
**Gene Name**	**Forward primers**	**Reverse primers**	**Proximal**	**Distal**
	**AA3SS (∆3SS=6)**			
daf-16	GCAATGCTTCATACTCCAGATG	TATCGTCTGGCGATTCGGAC	CGA AAG	TTT CAG
dli-1	CTGAGAACTGTGGAGTCACG	GTTGAATGCTGATCTTCGCG	CAA AAG	TTT TAG
sel-10	GTACAGCAATGGCTCTTCTTCC	CGGATTGAGGTCGTTGTTTCG	TAC CAG	TTC CAG
eri-9	AATCCTCAGGAAACTGGAACTG	TTGAACTGCAAATCTGGAGCC	GAC AAG	TTT CAG
ipla-3	TGAGCTCACCGATAATGAGG	ACAGAGTAATCGCCAGATGG	TTG TAG	TCT CAG
C06G1.1	AGCATCGTGGAAACTCATTTGG	GCAGTTGGAACTTGAAGTGGAG	CTT AAG	GTT CAG
ced-7	CAAGAATCAACGAACAGGTGAG	ATTTGGAGGGTTCAACGATGTC	CTG AAG	ATT CAG
gei-17	TCCCGAACGGAAATTATGAG	ACCAAGTGATTACTAGCTCC	TGT CAG	ATG CAG
	**AA3SS (∆3SS=9)**			
F56C9.10	GGATTTCAGCTCTTCGGATAAG	ATGTTGGAGGAGACGTTGTG	TTA ATT GAG	AAT GTG AAG
zyg-12	GAAGCTGTCACTCCACGATC	GCTCTTGAATACGCTCCGTTAG	GCC GCC TAG	ACC TTT CAG
H14E04.2	TATTGGAGGTGGCTCGTAC	GTACACTGAATGATAGGGAAAC	TTC TCC CAG	TTT TTT CAG
nsy-1	CCAGAAGCATGTGATCGTCC	TGGCAGGTTTCTTGTTGATCG	TAA TTT CAG	TAT TTC AAG
mdt-15	GCCTTCCGTGTTACAACCATC	AGCTCGATATCCGGGTGTTC	TTC AAT TAG	CTC TAT TAG
ceh-38	CAAGTGCTTCGCTTCTTTCATC	GCCGTGAATCTACATGAAGTG	TTT ATT CAG	AGA TTA CAG
	**AA3SS (∆3SS=12)**			
cup-5	CAAGGCATACAGGACAGGTTC	AAAGTGTGTCAAATGACCATCC	ATC TCG AGT TAG	GTC AAG TTT CAG
B0001.7	AGGCAATTTCCCAACGAGAC	GACGCTGCTCCTGATAATGG	TCT CAT TTA CAG	CCT CTA ATT CAG
	**RA3SS**			
slr-2	AAGTGATAAAATCGAAGAAGGTG	TCACTACTCCCTGAATTTTCGG	TTATATTTTTTCCAAAATAG	CTCATATTCGTCATCATCAG
sipa-1	TCCAGCTGTAAAGGGAATCG	ATATTGTTGGCTCGCTGTTC	GTTTTCATCGCGTGTGGCAG<br>TTCATCGCGTGTGGCAGCAG	TTCGACGAACGCACTTTCAG
fkh-7	TGTGAAAGGAGCTGTATGGACTG	CAAGAAGCGACAAAACACTGTC	ATTCGCACTTGTCAATTCAG	GCTCATTTTTGGAAATGCAG
spe-48	TGTCCAGTAAACTCCAACCCATC	AAGCCGTTTTCTCAGCTCTTCG	TACATTTTTTTTTTTTTGGAG	TATCGATTAATCATATTCAG
abu-12	GAGAATCAGCGTAAAGAGCAGAG	GTTGTTATACGGTTGTTGTGGTTG	GTTGCATGAGTCTAATTAAG	AATCAGCAAAGCATATTCAG
bath-44	ATGTGGACAATTCGTGGCTTC	ATTGCCTTGTGGATGTAATCG	ACGTAACTCCTTATTTCCAG	AAGATGAAAGTGGGTTGCAG
flp-1	GAAAACTGCGATGGTGATGCC	CAAATCTCAAGAAGTTAGGGTCAC	ACAACTTGGTGCTCTTTTAG	GGGAAAGAAAGCCGGTTCAG
cbp-1	TTTCGCTGCGAAGAATCAACC	TGGAGTATCTTGTATCCTTTTGAGTGG	CTGTTTGAAATCTCTTTCAG	AACTACAAAAAAATGTTCAG
attf-5	ACCAGTCAAAAAAACTCGAGGAC	TGCAGTAATGCTTTTCTCTCTTCC	TAAATATGTTTTCCTCTCAG	TGCACTGATTCCCGTTGCAG
rbm-5	TCACAACACCACTGGAAGACCTG	TGATCTGCTTCTGCTTCTACCTCC	TTAAATTAAAAATAATCCAG	CCAGTTTTTCCGGATTTCAG
R148.3	AAAGTTGAAGAGCCCGAGCC	TTCTTCTGGAGCCGGAGTTG	TGTGACGTTATTGTTGTCAG	ACGACTGTCAAGGATTGCAG
	**Minigene reporter**			
unc-93	GGAGCCAAATGCGCGTACATTACAG	CAACAAGAATTGGGACAACTCCAGTG	TTTAATCAAGCCA*NNNN*CAG	TTCATGATTGTAC*NNNN*CAG
attf-5	ACCAGTCAAAAAAACTCGAGGAC	CAACAAGAATTGGGACAACTCCAGTG	TAAATATGTTTTCCTCTCAG	TGCACTGATTCCC*N*T*N*GCAG

Table S7: RT-PCR primer sequences for validating SE, RI and MXE events.

Gene Name	RT-PCR primers for detecting alternative splicing		3'ss sequences		
	**Forward primers**	**Reverse primers**			
	**SE**		**Cassette exon**	**Downstream exon**	
C18B2.5	CTCGCACAGAAATCAGTATGC	CTCGTCTTCATAATCCGGCTG	GGTAAGTTGGTCCGTTTTA	AGTAAGTTCGTAATTTTCA	
nhx-5	CCATGCGACGCCATCTTTG	TGAAGGGCTCTGCACTGAC	ATGAACCGAAATCTATTCA	CAATTTTTATATATTTTCAG	
let-653	CCTGAAGATTTGACATACCAGG	TTGTAGCTGTCTCTGTAGTGG	GATTAGTTAAATATGTTTAG	AACGTTTTTTGTTGTTTCAG	
sao-1	ATTCTCAGCCACCATCGGAG	TGCATCTGTGGGATGTGCTG	ATCCGGCTATTCGAATTTA	ACAGTTCACACCAGTTTCA	
nstp-1	GTCTAAACCTTATCACATTCGCATC	TGTAACTACCACAACTGCACTG	TTACTGATTTTCTCATTCAG	ATTTTGAATCAAACATTCA	
tnt-3	AGTTCGAACGTCAACGCGTTC	CGCAGCTGGAATTGAAAGTCG	CAATAAACAATTTTCTTTAG	AACAAAATAAGTATTACCA	
zer-1	TTCCAACGCAATCACTACCAC	GAACCTCTTCATCTGCCTCAAC	TAACAAAAACAGTGTTGTA	AATCTGATGTTTTATTACAG	
alx-1	AGTGATTTTGCGTTTGCTCGG	TGGAGATTCTACACTTGGAGCAG	TTAGATTTTCGTGGTTTCAG	AGTTATTTTTTAAATTTCAG	
egl-8	ACGATTTACGACGAGACAAAGC	TTCCAGATGCACTTCCACATCC	TAATCTCGATCATTATTTAG	CTGAACTTGTTTTATCTCA	
hpo-3	GGATGTGATGGACAGTTTACACC	CAAATGCAAGCACGGAGATTGG	CTAATAAAATCTTTGTTCAG	TAATTTTGATAATGTTTCAG	
pac-1	AAAGCTGCGAATGGGAACTG	AACATCTTCGTTCATTGATGCC	GTTTTCACGTTTGAATTTAG	ATAAAACAATTAATTTCCAG	
lin-45	TGGAATGCTCTCGCGAAACG	GCGGAATGATGTCCAGATAACG	AATCACTAACAGCAATTTA	TAACTCATAATTATTTTCAG	
C30F12.2	CATATCAACCAACGAACAAGTC	CGTCGTTTCCATAATTTCATCG	AAATAATTGTGCTCCTTAAG	TATCGAAATATGTTTTCCA	
F25H2.6	TCTTCTTCTGTTCTTCGTCGTG	CGATTGTGGTGTAAAGAGAGTG	TTTGCATATTTCTCATTCAG	TAAAACATGTGCTATTGCA	
K11H3.8	TAGACAAAGTACAAGCGCAGTG	GTCAGCCTTTTCTCATCACTGG	TAATATTTCAAAATATTTAG	TATTCTCAAAATTATTACAG	
	**RI**		**3'ss in the intron**		
hum-8	ATCAATCGCAACTCCGTCAG	TGACTCTCAAATTCGTCTTCCG	AATAAAATGTGAAGATTTAG		
ero-1	CACAAATCAATGCGGAATCGC	AGTGCATACCATCTGACTCG	AAATTGGGGTAATTGTTCAG		
clec-180	GAAGGATCAAATGAGCACTG	CCGTAGACTTCTTATCGTCC	ACAATGTGATCAAGATTCAG		
clec-88	TTCTATCGATGAGTGGGAGG	ACAGTTGGCAAGTTGTGACC	TTTCAAAGAAAAAAATTCAG		
ubql-1	ACACCGTCCACTTGGTTATTAG	TCTCCATAGTTTGACGCATTAC	TACCAATAATCCATTTGCAG		
seu-1	GCTCATTATCCACAACGCAGAG	TGGCAGCAATGTTGTTTGAACG	GACGAATCTCATCAATCTAG		
	**MXE**		**Exon a**	**Exon b**	**Exon c**
unc-32	AAGAACGAGGAAACGCTGAAG	CATTACCACGACACGCTCTC	CTATTCGGTTCG TCACACAG	AATCTCCTTCACTA ACACAG	GTGTTTGTTTGA TTCTGCAG

References

1. Ragle,J.M., Katzman,S., Akers,T.F., Barberan-Soler,S. and Zahler,A.M. (2015) Coordinated tissue-specific regulation of adjacent alternative 3' splice sites *in C. elegans*. *Genome Res*, **25**, 982–994.

2. Ito,T., Muto,Y., Green,M.R. and Yokoyama,S. (1999) Solution structures of the first and second RNA-binding domains of human U2 small nuclear ribonucleoprotein particle auxiliary factor (U2AF(65)). *EMBO J*, **18**, 4523–4534.

3. Agrawal,A.A., Salsi,E., Chatrikhi,R., Henderson,S., Jenkins,J.L., Green,M.R., Ermolenko,D.N. and Kielkopf,C.L. (2016) An extended U2AF65–RNA-binding domain recognizes the 3′ splice site signal. *Nature Communications*, **7**, 10950.

4. Ma,L. and Horvitz,H.R. (2009) Mutations in the *Caenorhabditis elegans* U2AF large subunit UAF-1 alter the choice of a 3' splice site *in vivo*. *PLoS Genet*, **5**, e1000708.

5. Maji,D., Glasser,E., Henderson,S., Galardi,J., Pulvino,M.J., Jenkins,J.L. and Kielkopf,C.L. (2020) Representative cancer-associated U2AF2 mutations alter RNA interactions and splicing. *J Biol Chem*, **295**, 17148–17157.
